# A Mussel‐Inspired Bioadhesive Patch to Selectively Kill Glioblastoma Cells

**DOI:** 10.1002/advs.202510658

**Published:** 2026-01-27

**Authors:** Jose Bolaños‐Cardet, Sara Pugliese, Jordi Bruna, Daniel Ruiz‐Molina, Salvio Suárez‐García, Victor J. Yuste

**Affiliations:** ^1^ Catalan Institute of Nanoscience and Nanotechnology (ICN2) CSIC and BIST Bellaterra Spain; ^2^ Cell Death Senescence and Survival Group Departament De Bioquímica i Biologia Molecular and Institut de Neurociències Facultat de Medicina Campus De Bellaterra Universitat Autònoma de Barcelona Bellaterra Spain; ^3^ Unit of Neuro‐Oncology Hospital Universitari de Bellvitge‐Institut Català Oncologia Bellvitge Institute for Biomedical Research (IDIBELL) L'Hospitalet De Llobregat Spain; ^4^ Department of Cell Biology Physiology and Immunology Universitat Autònoma de Barcelona Bellaterra Spain

**Keywords:** antimicrobial, brain, glioblastoma, membrane, mussel‐inspired, polyphenols

## Abstract

Glioblastoma, the most prevalent and aggressive brain tumor, presents significant challenge due to its rapid proliferation, invasive nature, and resistance to conventional therapies. Current treatments, including surgery, radiation, and chemotherapy, frequently lead to recurrence, underscoring the urgent need for innovative solutions. This work develops and evaluates bioinspired adhesive membranes designed as novel strategy to address glioblastoma recurrence post‐surgery. Inspired by mussel adhesion, these membranes exhibit strong bioadhesion in wet environments and incorporate various phenolic‐based compounds. Among tested combinations, a membrane with catechin demonstrates specific cytotoxic effect on human glioblastoma cells. This effect is investigated through in vitro assays using glioblastoma cell lines, including primary cell cultures. Exposure to this membrane induces changes in cell morphology and internal structures, and alterations in cell adhesion and migration. Additionally, the use of glioblastoma spheroids and ex vivo tissues allow us to mimic glioblastoma microenvironment and assess the membrane efficacy. Reactive oxygen species are suggested to play a main role in the cytotoxic effect, counteracted by the antioxidant N‐acetylcysteine. Finally, a comprehensive proteomic study elucidates biological mechanisms underlying the membrane performance. This research highlights the potential of mussel‐inspired advanced scaffolds as a localized approach in glioblastoma therapy, suggesting a path for effective anticancer strategies.

## Graphical abstract

An innovative mussel‐inspired bioadhesive patch has been developed for post‐surgical glioblastoma treatment. The patch, which adheres strongly in biological environments, releases a localized treatment. This treatment, acting via reactive oxygen species, shows specific toxicity to glioblastoma cells. The mechanism was successfully investigated in various tumor cell models, demonstrating significant potential for preventing tumor recurrence after surgery.

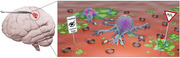

## 1 Introduction

The development of advanced materials as functional scaffolds has emerged as a promising solution in the fields of biomedical engineering and regenerative medicine [1, 2, 3, 4]. These materials are designed to provide structural support and enhance the biological functions of damaged tissues or organs. The need for such materials is driven by the increasing demand for effective treatments for various medical conditions, including chronic wounds, tissue degeneration, and cancer.

Cancer remains one of the most formidable challenges in modern medicine, with glioblastoma being the most common and aggressive primary malignant brain tumor. Glioblastoma is characterized by its rapid growth and infiltration into surrounding brain tissue. It represents 15% of all central nervous system (CNS) tumors and approximately 50% of CNS tumors [5]. Glioblastoma cells exhibit a high degree of cellular heterogeneity, expressing a variety of molecular markers and exhibiting diverse biological behaviors [6]. This heterogeneity allows glioblastoma cells to hijack normal brain signaling pathways, promoting tumor survival and resistance to conventional therapies.

The standard of care for glioblastoma involves a multimodality approach, combining surgery, radiation therapy, and chemotherapy [7]. Surgical resection is often performed to remove as much of the tumor as possible, followed by radiation therapy and concurrent and adjuvant temozolomide (TMZ) chemotherapy. However, even with this aggressive treatment regimen, recurrence is the norm, generally within one year of initial therapy [8, 9, 10, 11, 12]. The hallmark feature of glioblastoma is its infiltrative growth pattern, which makes complete surgical resection challenging and often leads to recurrence [13]. Notably, even after seemingly complete resection of the tumor, most recurrences occur in the surgical bed [14]. This observation has significant implications for the management and research of glioblastoma, suggesting that the surgical site, despite meticulous efforts to remove the tumor completely, retains a considerable risk of harboring residual tumor cells.

In this context, reactive oxygen species (ROS) play a significant role in the biology of glioblastoma. Glioblastoma cells often exhibit elevated ROS levels, which are involved in signaling pathways that promote tumor growth, survival, and resistance to therapy [15, 16, 17, 18]. ROS also contribute to the resistance of glioblastoma cells to conventional therapies, such as chemotherapy and radiation, by activating survival pathways and repair mechanisms. Modulating ROS levels can be a therapeutic strategy; for instance, increasing ROS beyond a certain threshold can induce cell death in glioblastoma cells [19]. Interestingly, combining ROS‐inducing agents with other treatments, such as TMZ, has shown enhanced cytotoxic effects compared to TMZ alone [20, 21]. Therefore, by carefully modulating ROS levels, it is possible to exploit the balance between ROS's role in promoting tumor survival and its potential to induce tumor cell death, offering a promising approach for developing more effective treatments for glioblastoma. Nevertheless, most of the treatments rely on the systemic administration of chemotherapeutics, which face additional obstacles in terms of i) effectiveness, ii) difficulty in crossing the blood‐brain barrier, and iii) side effects, among others [22, 23, 24].

In this scenario, innovative approaches are urgently needed to address this devastating disease and fulfil the significant unmet clinical need. One disruptive approach could be the development of specific scaffolds, such as membranes or bioadhesive patches, designed for application during the “window of opportunity” immediately following tumor resection. By placing these scaffolds in the surgical bed before the wound is closed, surgeons can exploit this critical timeframe to establish a platform for localized delivery. This intervention aims to target residual microscopic disease and prevent glioblastoma recurrence [25]. Such membranes must demonstrate bioadhesion, biocompatibility, and the capacity to selectively eliminate cancer cells, for example, by modulating ROS production. Catechol and polyphenolic‐based compounds are well known as ROS modulators and key players in natural‐based adhesive structures [26, 27, 28, 29, 30]. With these features, these molecules emerge as excellent candidates for their use as constituents in advanced scaffolds [31].

Up to now, only two examples have reported the synthesis of isolable phenol‐based membranes. In both cases, the authors reported the use of an amine‐rich ligand (polyethyleneimine, PEI). In the first case, Lee et al. described the formation of a membrane in the air‐liquid interface by reacting dopamine and PEI [32]. Briefly, dopamine self‐polymerizes (PDA), forming a brittle film with many cracks. By adding PEI, the robustness of PDA is enhanced, obtaining a membrane. In the second case, Zhi‐Kang et al. also reported the formation of a membrane by polymezising dopamine in the presence of PEI at the air‐liquid interface [33]. The authors described the formation of an asymmetric structure due to the assembly of PDA/PEI aggregates growing in the solution. As can be noticed in both cases, the membranes are only suitable when the PDA is formed previously and PEI is added as a crosslinking agent for the generation of an in situ substrate, enhancing the cohesion. The integration of PEI allowed for the fabrication of robust membranes; however, the resulting membranes are highly restricted to other catechol‐based compounds and show several limitations in terms of thickness, biodegradability, adhesion and biocompatibility, thus their potential application in medicine are completely hindered. For these reasons, finding a new strategy that allows for forming a wide range of catechol‐based membranes in an easy, eco‐friendly and reproducible way is a pressing concern. Therefore, the final product obtained should be easy to handle (robust and flexible) without the need for substrate or side molecules (e.g., PEI or chitosan) [34].

**SCHEME 1:**
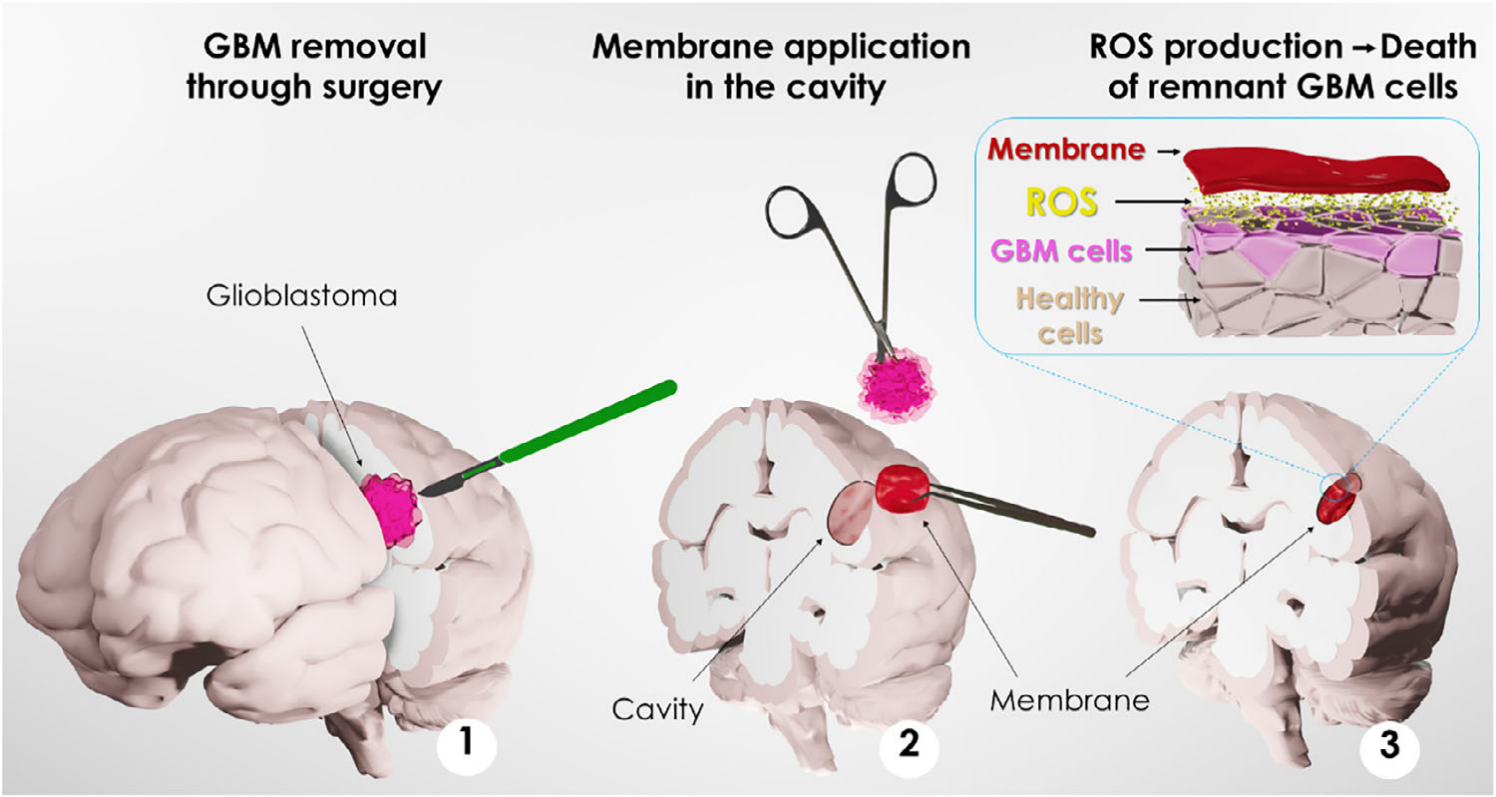
Representation of the applicability of the **CATH‐M** membrane in glioblastoma treatment.

In addition to addressing the risks of infections during surgical procedures and the growing concern of antimicrobial resistance, it is imperative to endow these advanced materials with antimicrobial properties [35, 36]. Recently we have shown that polyphenols, particularly when polymerized into catecholamine‐based coatings, can significantly reduce microbial populations [37]. However, the challenge remains in developing these materials into ready‐to‐use membranes that actively treat tissue‐related diseases by incorporating antimicrobial, anticancer and bioadhesive properties [38, 39]. An ideal treatment approach is shown in Scheme 1.

To overcome these challenges, we have developed a straightforward process to produce bioinspired adhesive membranes that could potentially be evaluated as a patch to address glioblastoma recurrence after a surgery (Scheme 1). This family of bioinspired membranes is generally bioadhesive in wet environments, thanks to their structure inspired by mussel foot proteins. Additionally, using this approach it is possible to incorporate different catechol and phenolic‐based compounds, opening a broad range of combinations to select the most suitable one in terms of adhesion and ROS modulation capacity. Interestingly, the developed membranes demonstrated intrinsic antimicrobial activity, offering effective protection against infections during the implementation into the surgical bed and during the entire process. For the formation of the membranes, we have used a broad range of catechol and phenolic‐based molecules, from the simplest one (e.g., pyrocatechol) to those with higher OH‐content (e.g., catechin), with different ROS generation behaviors for study and comparison. Among the different combinations, one specifically showed promising application for glioblastoma treatment. Following the stablished protocol, it was possible to incorporate catechin (well‐known for its anticancer properties) [40, 41, 42, 43, 44], as the catechol/polyphenol‐based molecule, for the formation of a promising membrane that was fully evaluated. The comprehensive analysis includes in vitro experiments using various glioblastoma cell lines, including primary cell cultures and ex vivo tissues and spheroids to mimic relevant environments. Additionally, proteomic studies have been conducted to elucidate and understand the mechanisms behind.

## 2 Results and Discussion

### 2.1 Synthesis of the Bioinspired Polyphenol‐Based Membranes

**FIGURE 1:**
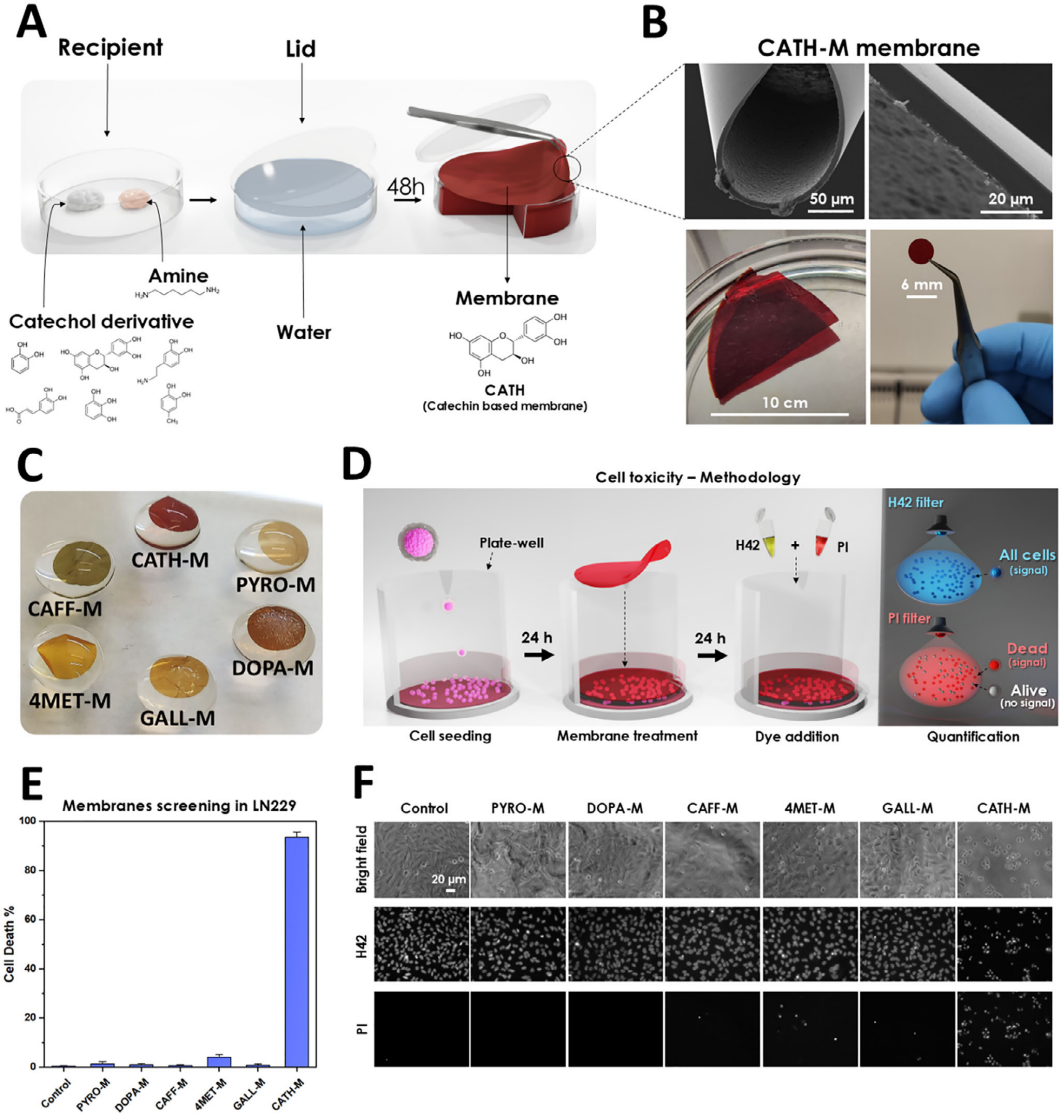
Catechol‐based membranes. (A) Synthesis scheme of the catechol and polyphenol‐based membranes, following straightforward procedure under mild conditions. (B) Scanning electron microscopy (SEM) and optical images of the catechin‐based membrane (**CATH‐M**) selected in this study for its singular properties, highlighting its manipulability. (C) Macroscopic look of the six different HMDA‐based membranes floating in a drop of water. (D) Methodology used to study the percentage of cell death in a cell monolayer, in which the membrane was in close contact (not touching the cells but near them). Cell death was measured after the treatment with a Hoechst33342 (H42) and propidium iodide (PI) staining. (E) Comparison of the cytotoxicity between different catechol‐based membranes in the LN229 cell line, where **CATH‐M** was the only one achieving a considerable cell death percentage. (F) Cytotoxicity analysis of each membrane under fluorescence microscopy using the dyes H42, (which can pass through intact cell membranes) and PI (which can only enter cells that have suffered membrane damage). Data has been represented as the mean ± standard deviation (N = 5).

Bioinspired membranes were developed after the combination of hexamethylenediamine (HMDA) with different phenolic derivatives: pyrocatechol (PYRO), caffeic acid (CAFF), pyrogallol (GALL), dopamine (DOPA), 4‐methylcatechol (4MET) and catechin (CATH). The resulting six synthetized membranes were named as **PYRO‐M**, **CAFF‐M**, **GALL‐M**, **DOPA‐M**, **4MET‐M** and **CATH‐M**, respectively. Briefly, specific phenolic derivatives and HMDA were weighted separately and mixed in ultrapure water (Figure 1A; Figure S1A and Video S1). After a 48 h of incubation period, floating membranes were formed at the air‐water interface, allowing for easy manipulation and shaping (Figure 1B; Figure S1B and Video S2).

#### 2.1.1 Physicochemical and Morphological Characterisation

The polymerization of the membranes was thoroughly investigated using various techniques. Initially, FTIR analysis was conducted to identify the primary bonding structures. The results clearly indicated the presence of both amine and catechol/quinonic moieties in all membranes (Figure S2). The spectra consistently exhibited similar features, including a characteristic broad band around 3250 cm^−^^1^, attributed to the stretching vibrations of hydroxyl (–OH) and amine (–NH_2_) groups from phenols and amines, respectively. Additionally, the peak around 2850 cm^−^^1^ was associated with aliphatic carbons (C–H), mainly present in the HMDA ligand. Notably, the peak at 1260 cm^−^^1^ could be assigned to a secondary amine binding to an alkyl or aromatic ring, indicating successful catecholamine polymerization. Finally, the peaks at 1700 cm^−^^1^ and around 1500 cm^−^^1^ were attributed to quinones (C = O) and C = C–H and C = C vibrations from the catecholic/quinonic rings, respectively. Furthermore, other specific groups were identified. For instance, in **DOPA‐M**, a peak at 1000 cm^−^^1^ could be associated to free amine (–NH_2_) groups, present in both HMDA and DOPA. On the other hand, the carboxylic groups (–COOH) present in **CAFF‐M** were confirmed by a distinct band at 1270 cm^−^^1^. Notably, in the case of **CATH‐M**, the intensity of the peak corresponding to –OH groups were significantly higher compared to the other membranes (Figure S2). This indicated a greater abundance of free hydroxyl groups incorporated by the catechin molecules. Such an increase in hydroxyl groups could lead to higher reactivity in the presence of oxygen and wet environments, directly impacting the production of ROS and its biological implications.

Once the polymerization process was confirmed, the most superficial composition was characterized to determine the exposed functional groups. This is relevant as it can be determinant to establish different interactions in the biointerface between the membrane, the tissue, and the cells. To achieve this, high‐resolution X‐ray photoelectron spectroscopy (XPS) curve‐fitting was performed (Figure S3). In all the synthesized membranes, C1s spectra showed three different chemical environments. Apart from the aliphatic component, the existence of the other two contributions confirmed that catechol coexists in its two forms: fully reduced (C–OH) and quinonic (C = O). In the case of **CATH‐M** the C = O band was significantly higher, thus confirming its potential for ROS production by undergoing further oxidation in atmospheric environments. O1s spectra support this statement, as the same contributions appear, including a third one corresponding to the semiquinone form (C–O). In the case of N1s spectra, it showed free (R–NH_2_) and aliphatic (R–N–R) amine‐related contributions, associating the first one to unreacted amine tail ends of the HMDA and the second one to the result of cross‐linking. The presence of hydroxyl and quinones groups is of special interest due to: i) offering interaction and strong adhesion and ii) be further oxidized, thus producing ROS during the process.

After identifying the exposed functional groups, elemental analysis was conducted to determine the percentage of CHON of the synthesized membranes, in order to provide fundamental insights into their chemical composition (Table S1). Interestingly, despite the reactions start with an excess of amine, the average ratio found in the membranes was of 0.8 HMDA molecules per catechol. Notably, these results underscored reproducibility, as measurements from three different batches displayed low error rates.

The choice of the catechol derivative affected not only the chromophoric traits and chemical composition, but also the physical properties of the resulting membranes (Figure 1C; Figure S4A). For instance, PYRO was characterized for inducing the most homogeneous and flexible membranes. While GALL‐based membranes have a considerable heterogenicity, seem to have an enhanced surface adhesion. The combination of HMDA with the catechol derivatives DOPA or CAFF, produced membranes with an easy manipulability and flexibility, respectively. In the case of 4MET, the resulting membranes was the most brittle, and by using CATH allowed for the formation of the thickest and most manipulable membrane. From a morphological point of view, scanning electron microscopy (SEM) showed that the six different membranes shared some patterns in their topography and a Janus‐like morphology (Figure S4A). Whereas the air‐face in all the cases was characterized by a flat or mostly flat surface, the water‐face yielded a particle‐embedded face, observing in this case a higher variability between membranes (being **PYRO‐M** exhibiting the smoothest surface and the **CATH‐M** the highest roughness). In terms of thickness, most of the membranes measured between 1 and 3 µm. **PYRO‐M** was the thinnest, ranging from 0.7 to 1 µm, whereas **CATH‐M** was significantly thicker, measuring between 10 and 12 µm (Figure S4A). Although the membranes were initially hydrophobic when completely dried, they were able to absorb water and become turgid, thereby turning hydrophilic. This suggests a permeable nature, despite the absence of a porous structure in the cross‐section analysis (Figure S4B).

#### 2.1.2 Cytotoxicity Activity Screening Against Glioblastoma Cells

To comprehensively evaluate the cytotoxic activity against glioblastoma, the six synthesized membranes were subjected to a screening assessment against the human glioblastoma cell line LN229 (Figure 1D–F). A standardized experimental protocol was followed, involving the incubation of 2 × 10^4^ LN229 cells with individual membrane discs for 24 h. Following this incubation period, the percentage of cell death was quantified using a combination of Hoechst 33342 (H42) and propidium iodide (PI) staining. H42 staining enabled the enumeration of total cells, while PI staining specifically targeted dead cells.

The results of this cytotoxicity assay were both intriguing and remarkable. While most of the polyphenol‐based membranes exhibited minimal cytotoxicity, the **CATH‐M** membrane demonstrated a remarkably anti‐tumor effect in vitro. The **CATH‐M** membrane induced cell death in approximately 90% of the LN229 cells, far surpassing the cytotoxicity levels observed with the other five membranes. In contrast, the remaining membranes failed to induce substantial cell death, with cytotoxicity rates remaining below 5%.

### 2.2 CATH‐M and Its Suitability for Glioblastoma Post‐Surgical Treatment

Given the exceptional cytotoxic activity displayed by the **CATH‐M** membrane, it was identified as a highly promising platform for further mechanistic investigation. Conversely, the **PYRO‐M** membrane, exhibiting negligible cytotoxicity, was selected as an appropriate negative control for comparison purposes (Figure 1D). The selection of **PYRO‐M** as a negative control, besides its interesting physical properties, provided a valuable benchmark against which to compare the cytotoxic effects of the **CATH‐M**, ensuring a robust and reliable assessment of their anti‐tumor potential. Subsequent studies were conducted equally for both membranes.

#### 2.2.1 Physiological Degradation and Stability

To evaluate the biodegradability of the selected membrane in different media, **CATH‐M** and **PYRO‐M** were stored for up to 8 months at 37°C in water, 100% ethanol, pH 4 solution, pH 10 solution, phosphate‐buffered saline (PBS), culture media, or trypsin (0.25% trypsin and 1 mM EDTA). The evolution of the membranes was monitored over time (Figure S5) and characterized using SEM (Figure S6A). While the **PYRO‐M** showed no consistent changes after 8 months, its macroscopic shape was altered when immersed in pH 10 solution. In contrast, the **CATH‐M** displayed more noticeable changes under basic conditions, becoming more transparent within the first month and causing the medium to change color. Ultimately, the **CATH‐M** almost fully degraded after 8 months under these conditions (Figure S6C). These observations suggest that the change in pH may induce changes in the bonding structure, leading to membrane weakening and highlighting its reactivity. Additionally, subtle differences were observed on the surface of the **CATH‐M** membrane after 8 months when comparing water, culture media, and trypsin (Figure S6B). In cell culture media, the **CATH‐M** surface became smoother, with nearly complete closure of the pores on the air‐facing side and a more even appearance on the water‐facing side. This was likely attributed to interactions with proteins and amino acids present in the culture media. Conversely, exposure to trypsin resulted in a slightly eroded surface with more rugosities and membrane protuberances, particularly on the air‐face side. This effect is probably due to the protease activity of trypsin, which degrade overtime.

To evaluate the in‐depth stability of the **CATH‐M** membrane, a sample was subjected to humid conditions and analyzed using various techniques. After 30 days of storage in this environment, XPS analysis revealed no significant variations in the membrane's surface chemistry (Figure S7A). Similarly, elemental analysis demonstrated the chemical invariability of the membrane (Figure S7B). These findings, coupled with observations from SEM imaging confirming morphological stability (Figure S7C), collectively indicate the robust chemical and morphological stability of the **CATH‐M** membranes. These findings highlight the **CATH‐M** potential for its application in the human body, thanks to both its stability and suggested biodegradability profile. The membrane remains stable during the necessary period for the expected treatment, ensuring its functionality and effectiveness. Over time, it progressively degrades, minimizing the risk of long‐term complications.

#### 2.2.2 Antibacterial Activity

Considering the cytotoxic potential of **CATH‐M**, in addition to the antimicrobial activity found in coatings with a similar formulation,^20^ the antibacterial properties were also evaluated. Methicillin‐Resistant *S. aureus* (MRSA) and *E. coli*, common microorganisms found in post‐craniotomy infections, were selected as representative Gram‐positive and Gram‐negative bacteria, respectively [45, 46]. After the **CATH‐M** remained inside a dense bacterial suspension for 24 h, **CATH‐M** demonstrated exceptional antibacterial activity, achieving a Colony Forming Units (CFU) reduction of over 99.999% for MRSA and 99.99% for *E. coli*. In contrast, **PYRO‐M** exhibited a slight reduction (approximately 50%) in Gram‐negative *E. coli* (Figure S8A,C). We also measured ROS generation in the bacterial solutions after the incubation time. The levels of ROS observed, for both membranes and bacteria aligned with the CFU reduction results, further supporting the antibacterial efficacy of **CATH‐M** (Figure S8B).

The importance of having intrinsic antimicrobial activity in a patch for potential glioblastoma post‐surgery treatment cannot be overstated. The objective is to reduce the risk of post‐operative infections, which are a major concern in neurosurgical procedures. By effectively eliminating pathogenic bacteria, these membranes can enhance patient outcomes, reduce the need for additional antibiotic treatments, and ultimately contribute to a more successful recovery process.

#### 2.2.3 Ex Vivo Adhesion and Brain Conformability of **CATH‐M**

The assessment of brain conformability and adhesion properties is crucial to ensure the suitability of **CATH‐M** for its use in brain and glioblastoma post‐surgical treatment, where both mechanical properties and tissue interaction are crucial.

**FIGURE 2:**
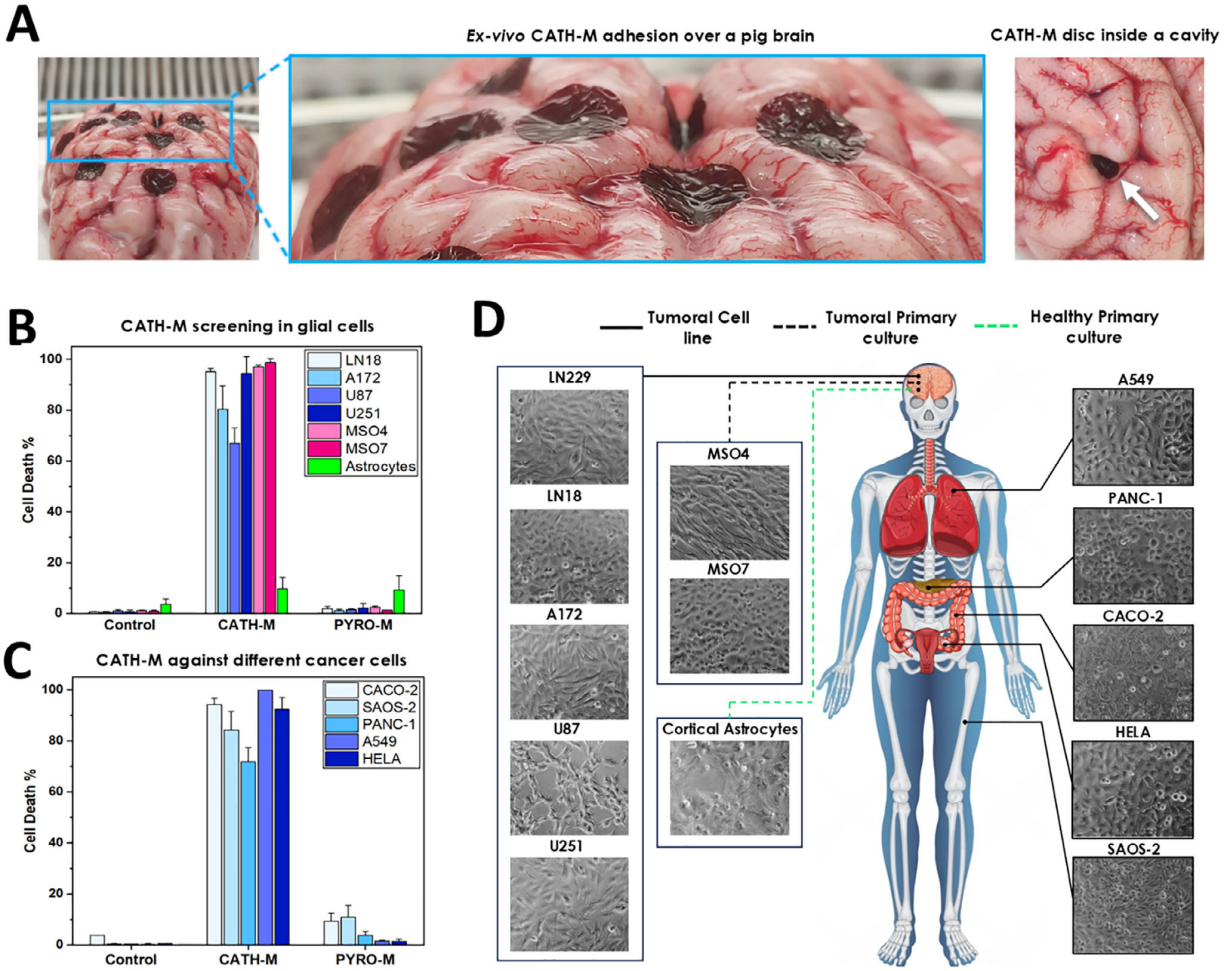
**CATH‐M** generates a strong and broad‐spectrum cytotoxicity in glioblastoma and cancer‐derived cells. (A) Representative images of pieces of **CATH‐M** in an ex vivo pig brain and its ability to adapt with high conformability and remain attached to the different morphologies of this organ. (B) Cell death screening of the **CATH‐M** and **PYRO‐M** membranes against 7 human‐derived glioblastoma cells (5 cell lines and 2 primary cultures), healthy rat astrocytes (primary culture) and (C) 5 human‐derived tumor cells (from different organs). In all the cases but for the astrocytes, **CATH‐M** achieved a percentage of cell death around 80%. (D) Schematic representation of all the cells tested against the **CATH‐M** membrane and their origin. A total of eleven different tumoral cells (five glioblastoma cell lines, two glioblastoma primary cultures and five different organ‐derived tumor cell lines) and healthy astrocytes isolated from rat were used. Data has been represented as the mean ± standard deviation (N = 5) from 3, 2 and 1 independent tests for Glioblastoma cells, astrocytes and organ‐derived cancer cells, respectively.

To evaluate these properties a range of qualitative and quantitative tests have been performed. Initially, to check the conformability, **CATH‐M** membranes were cut into round discs and placed as patches in different areas of an ex vivo pig brain (Figure 2A; Figure S9). These tests showed that **CATH‐M** conformed perfectly to the surface of the brain, adhering tightly to the gyral and sulcal regions regardless of spatial orientation. Besides, the membranes were easily applied or removed without breaking (Video S3), showcasing its flexibility and stability during use. Once positioned in the desired area, **CATH‐M** remained securely in place, maintaining its adhesion under high humidity conditions, closely mimicking the realistic environment in the surgical site. This ensures that **CATH‐M** can effectively stay in place and perform its intended function without displacement, which is crucial for a successful post‐surgical treatment.

To further evaluate the membrane's performance, several figures of merit have been analyzed. The first test involved measuring the shear adhesion force, where a **CATH‐M** membrane (with a contact area of 2 cm^2^) was placed on a rabbit brain. The membrane was subjected to increasing weights via a pulley system, which applied a controlled tensile force (Figure S10). As the weight gradually increased, the adhesion force was recorded until the membrane detached. The membrane remained securely adhered under weights up to 10 g, corresponding to a force per unit area of 49 mN/cm^2^.

Additionally, a tensile test was performed using a nanotensile setup to determine the elastic modulus of the material. The stress–strain curve revealed a linear behavior in the elastic region, with a modulus of approximately 10 MPa up to ∼0.05% strain, indicating elastic deformation (Figure S11A,B). As the strain increased, a gradual curvature marked the onset of plastic deformation, with material failure occurring at 57 MPa and 0.2% strain, signaling a transition to rupture (Figure S11A). These results demonstrate that **CATH‐M** exhibits a mechanical performance compatible with biological tissues, whose typical modulus is in the kPa range, ensuring its suitability for biological applications [47]. Lastly, a shear force test was performed using the same nanotensile setup to measure the adhesion between **CATH‐M** and a biological substrate (with a contact area of 0.8 cm^2^). The adhesion force profile showed a critical adhesion force of 16 mN/cm^2^ marking the point where the membrane began to resist tangential sliding (Figure S11C). Beyond this point, the system entered a steady‐state sliding regime, with a frictional force oscillating around 10 mN, indicating stable interfacial shear bonding (Figure S11C).

These combined results support **CATH‐M** as a mechanically suitable material for post‐surgical applications, demonstrating both excellent conformability to the brain surface and robust adhesion under realistic physiological conditions.

#### 2.2.4 Cell Death Spectrum of the **CATH‐M** Membrane

Focusing on the **CATH‐M** and **PYRO‐M** and following the previous promising results observed in LN229 cell line, we expanded our research to four additional stablished human glioblastoma‐derived cell lines (LN18, A172, U87, and U251), two primary cultures isolated from patients (MSO4 and MSO7) and astrocytes isolated from rat. Initial seeding densities were set at 1.8 × 10^4^, 1.8 × 10^4^, 1.5 × 10^4^, 1 × 10^4^, 2.4 × 10^4^, 2.6 × 10^4^ (96‐well plate) and 5 × 10^5^ (24‐well plate) cells/well respectively. After a 24‐h incubation period, all six glioblastoma‐derived cell lines exhibited a similar pattern of cell death as previously observed in LN229. Specifically, **PYRO‐M** remained innocuous, while **CATH‐M** consistently induced cell death rates exceeding 80% in most of the cases, reaffirming its broad cytotoxic spectrum against glioblastoma cells (Figure 2B; Figure S12). Interestingly, astrocytes exhibited a considerably lower cell death (less than 20%) when exposed to **CATH‐M**, which was similar to **PYRO‐M** (suggesting mechanical stress rather than cytotoxic effect) (Figure 2B; Figure S13). To further demonstrate the broad applicability of **CATH‐M**, the same experiment was performed with the human‐derived tumor cell lines A549, PANC‐1, CACO‐2, SAOS‐2 and HeLa, which are derived from lung carcinoma, pancreatic carcinoma, colorectal adenocarcinoma, osteosarcoma and cervical carcinoma, respectively. In this case, 1 × 10^4^, 2 × 10^4^, 2 × 10^4^, 2.5 × 10^4^ and 1 × 10^4^ cells/well were seeded. After 24 h of incubation, the cell death induced by **CATH‐M** remained also near the 80% in all the cases, with the only difference being that **PYRO‐M** exhibited some cytotoxicity in certain cases (Figure 2**C**; Figure **S13**). These results, aligned with previous findings, confirmed the broad spectrum of **CATH‐M** against different cancers, which highlights its potential application scope (Figure 2D).

#### 2.2.5 Cell Death Kinetics, Survivability and Viability

**FIGURE 3:**
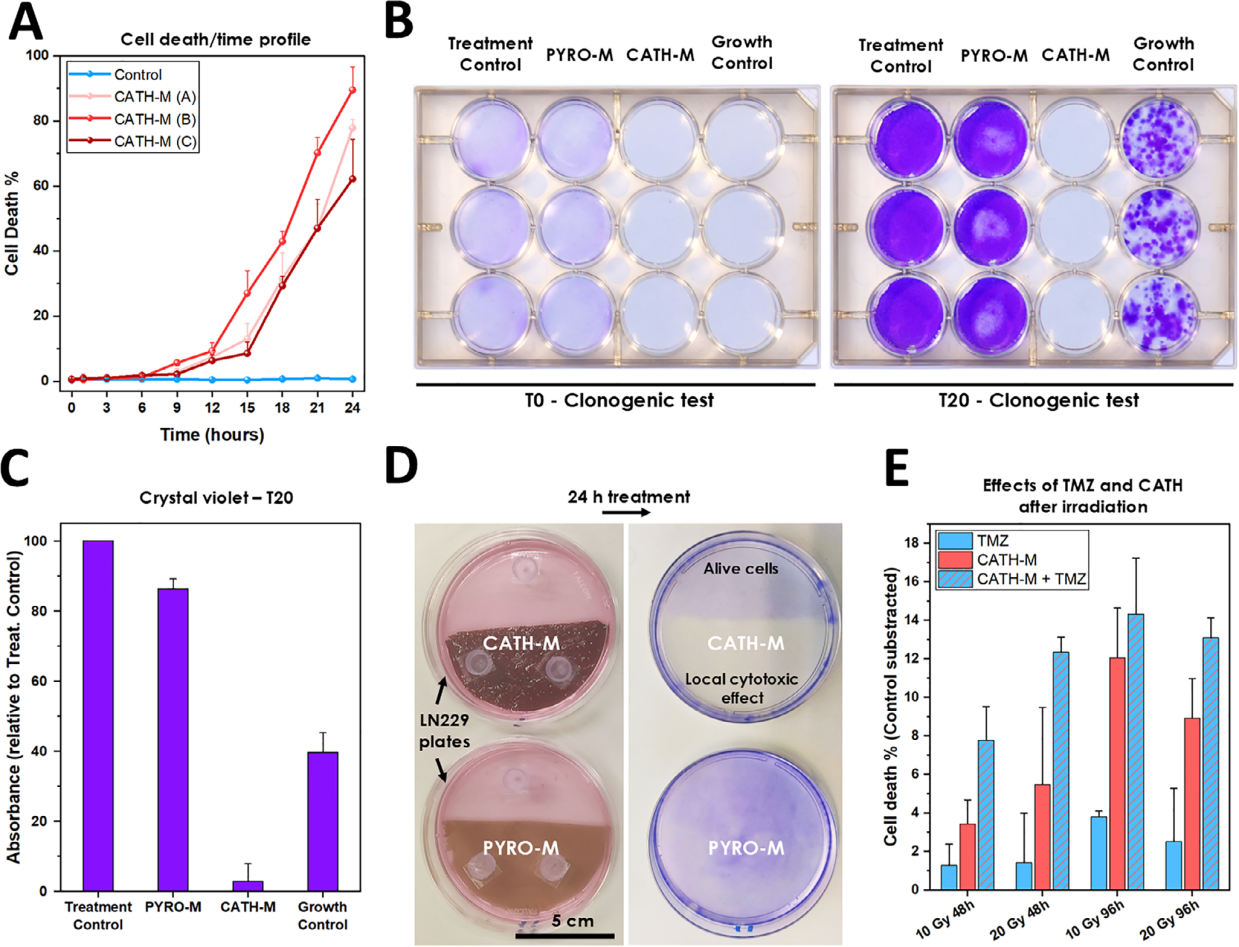
Characteristics of **CATH‐M**‐induced cytotoxicity. (A) LN229 cell death profile in function of the exposure time of three different **CATH‐M** membranes. Represented as the mean ± standard deviation (N = 5). (B) The clonogenic assay performed for 20 days confirms that LN229 cells should not be able to survive and multiply after being successfully treated with the **CATH‐M** membrane. (C) Quantification of the retained crystal violet by the cells in each condition (present in the 20 days plates) of the clonogenic test. Represented as the mean ± standard deviation relative to the control (N = 6). (D) **CATH‐M** induces a local cytotoxicity, only affecting the cells in close contact with the membrane (half of the plate), while **PYRO‐M** remains innocuous. (E) Comparison of the **CATH‐M** and temozolomide (TMZ) treatment over irradiated LN229 cells, together or separated, at different irradiation intensities (Gy) and treatment time. Represented as the mean of each condition subtracted from the control, ± standard deviation (N = 5).

To comprehensively evaluate the cytotoxic profile of **CATH‐M**, a time‐course study in LN229 cells was conducted. Our findings revealed a linear increase in cell death percentage within the first 24 h, suggesting a consistent rate of cell killing (Figure 3A; Figure S14). Next, in order to determine the survivability and viability of LN229 cells exposed to **CATH‐M**, a clonogenic assay was performed. A monolayer of 250 000 cells was treated with the membrane for 24 h, followed by its removal. We then assessed the ability of the damaged cells to recover and proliferate over a 20‐day period, comparing their growth to that of a control group of 1000 cells. While **PYRO‐M** treatment resulted in minimal changes beyond expected mechanical damage, a remarkable 9 out of 12 replicates of **CATH‐M**‐treated cells exhibited no signs of survival after the 20‐day incubation period (Figure 3B; Figure S15). The relative cell numbers in each condition were quantified by measuring crystal violet absorption following fixation (Figure 3C). Notably, the surviving clones in the **CATH‐M**‐treated group were predominantly located at the well edges, where direct contact with the membrane was limited, reinforcing the notion of its localized effect (Figure S15A). To precisely evaluate the localized effects of ROS generated by the developed membrane, a targeted experimental approach was conducted. LN229 cell plates were prepared, and either a piece of **CATH‐M** or **PYRO‐M** was strategically placed on only one half of each plate. After a 24‐h incubation period, distinct outcomes were observed. The **PYRO‐M** membrane showed no discernible difference in cell viability between the treated and untreated halves of the plate, indicating a lack of significant local effect. In contrast, the **CATH‐M** membrane successfully induced cell death exclusively in the region directly in contact with it, leaving cells on the untreated half of the same plate viable (Figure 3D; Figure S16). This compelling result unequivocally confirms the highly localized cytotoxic effect of the **CATH‐M**, highlighting its ability to deliver ROS‐mediated damage precisely where needed.

#### 2.2.6 Comparison With Standard of Care Treatments

To investigate the potential interactions between **CATH‐M** treatment and existing glioblastoma therapies, we conducted a comparative analysis of its cytotoxicity against TMZ. We also assessed their combined effects on LN229 cells (4.0 × 10^4^ cells/well) subjected to irradiation doses of 10 or 20 Gray (Gy), followed by 48 and 96 h incubation and then treated for 24 h with the membrane, TMZ or both. Our findings revealed that **CATH‐M** consistently exhibited superior cytotoxicity compared to TMZ (Figure 3E; Figure S17). Furthermore, the concurrent administration of **CATH‐M** and TMZ (0.25 mM) yielded in a higher effect across all exposure time. These results suggested that integrating **CATH‐M** with current glioblastoma treatment protocols (irradiation plus TMZ) could potentially enhance therapeutic outcomes without compromising the existing standard of care.

#### 2.2.7 Identification of the Cell Death Subroutine(s) Engaged by CATH‐M in GBM Cells

**FIGURE 4:**
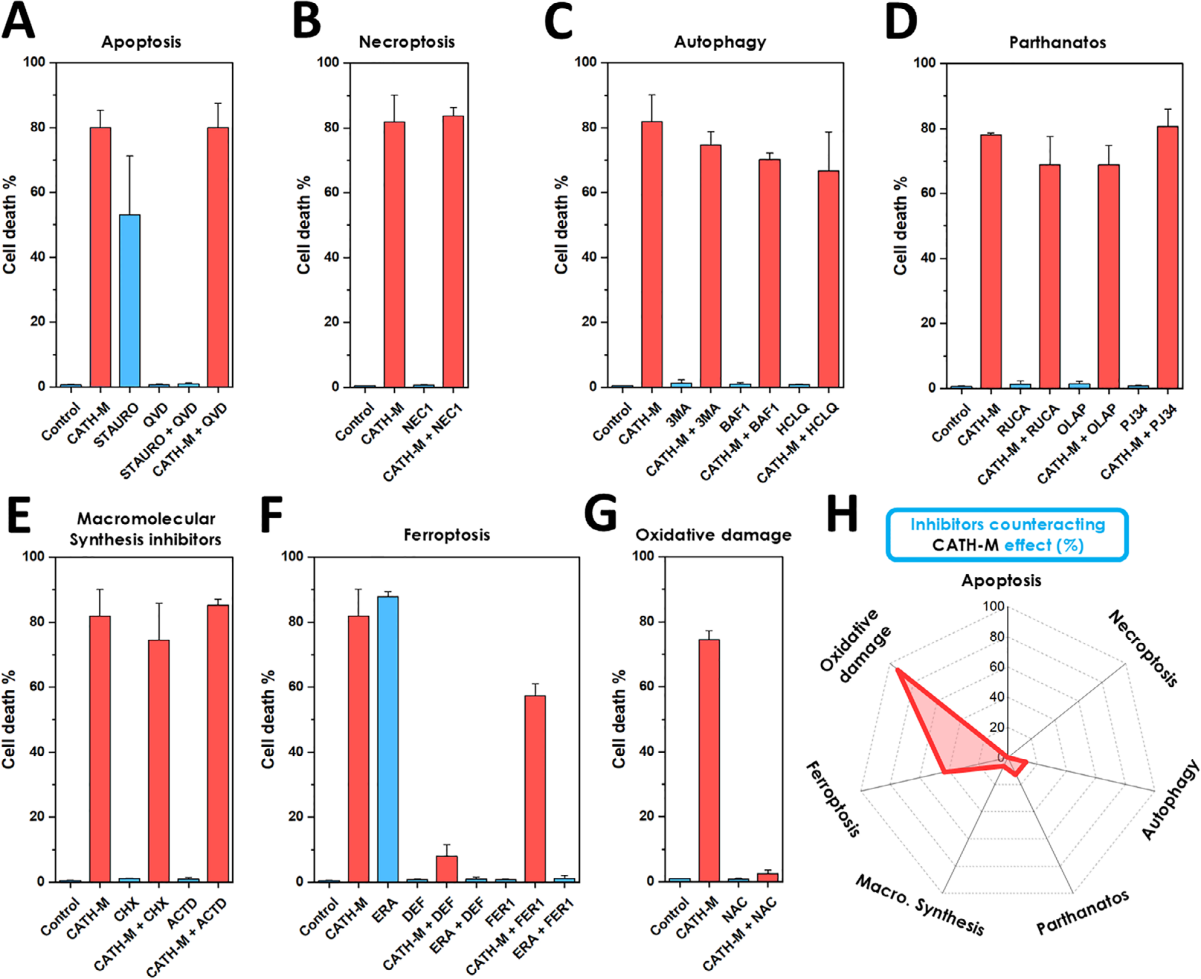
**CATH‐M**‐triggered cell death analysis. LN229 Cell death percentage when exposed to **CATH‐M** and simultaneously with inhibitors of (A) apoptosis, (B) necroptosis, (C) autophagy, (D) parthanatos, (E) macromolecular synthesis, (F) ferroptosis, and (G) oxidative damage. Blue and red bars correspond to the absence or presence of **CATH‐M** during the treatment, respectively. (H) From the inhibitors selected, only the chelating agent and ferroptosis inhibitor deferoxamine (DEF) and, specially, the antioxidant NAC showed a significant decrease in the percentage of cell death. Data has been represented as the mean ± standard deviation (N = 5).

To elucidate the underlying mechanism of cell death induced by **CATH‐M**, we tested various inhibitors targeting different pathways: A) apoptosis, B) necroptosis, C) autophagy, D) parthanatos (PARP inhibitors), E) macromolecular synthesis, F) ferroptosis and G) oxidative damage) in conjunction with the membrane. The specific concentrations and abbreviations of the compounds within each section were as follows: A) Q‐VD‐OPH (QVD) 50 µM, staurosporine (STAURO) 1 µM; B) necrostatin‐1 (NEC1) 50 µM; C) 3‐methyladenine (3MA) 10 mM, bafilomycin (BAFI) 100 nM, hydroxychloroquine (HCLQ) 20 µM; D) rucaparib (RUCA) 10 µM, olaparib (OLAP) 10 µM, PJ34 10 µM; E) cycloheximide (CHX) 10 µg/mL, actinomycin D (ACTD) 10 nM; F) ferrostatin‐1 (FER1) 10 µM, deferoxamine (DEF) 100 µM, erastine (ERA) 10 µM; G) n‐acetylcysteine (NAC) 5 mM (Table S2). While QVD effectively inhibited apoptosis induced by staurosporine, a well‐known apoptosis inducer [48], it failed to protect LN229 cells from **CATH‐M**‐induced cell death, suggesting a non‐apoptotic mechanism (Figure 4A; Figure S18). Similarly, the necroptosis inhibitor NEC1 did not provide protection (Figure 4B; Figure S19), indicating that necroptosis was also not the primary mechanism of cell death triggered by the **CATH‐M** membrane. Autophagy (Figure S20) and parthanatos (Figure S21) inhibitors both displayed marginal reductions in cell death (Figure 4C, D), suggesting that these pathways might play a minor role in the overall cell death process. Macromolecular synthesis inhibitors also showed no significant difference compared to the membrane alone (Figure 4E; Figure S22). While ferroptosis inhibitors successfully protected cells from the ferroptosis inducer erastine (ERA) [49], only the iron chelator deferoxamine (DEF) achieved a significant decrease in cell death, with a slight effect observed with ferrostatin 1 (FER1) (Figure 4F; Figure S23). However, N‐acetylcysteine (NAC), an inhibitor of oxidative damage, completely protected cells from **CATH‐M**‐induced cell death (Figure 4G; Figure S24). These findings strongly implicate oxidative stress as the primary driver of cell death induced by **CATH‐M**. Considering the lack of protection from apoptosis and necroptosis, coupled with the slightly protection observed by ferroptosis inhibitors and the complete protection afforded by NAC, it is proposed that the cell death induced by **CATH‐M** is predominantly a necrosis due to oxidative damage (Figure 4H).

### 2.3 Influence of CATH‐M in Cell Adhesion and Migration

#### 2.3.1 Cell Morphology and Tumor‐Like Environment

**FIGURE 5:**
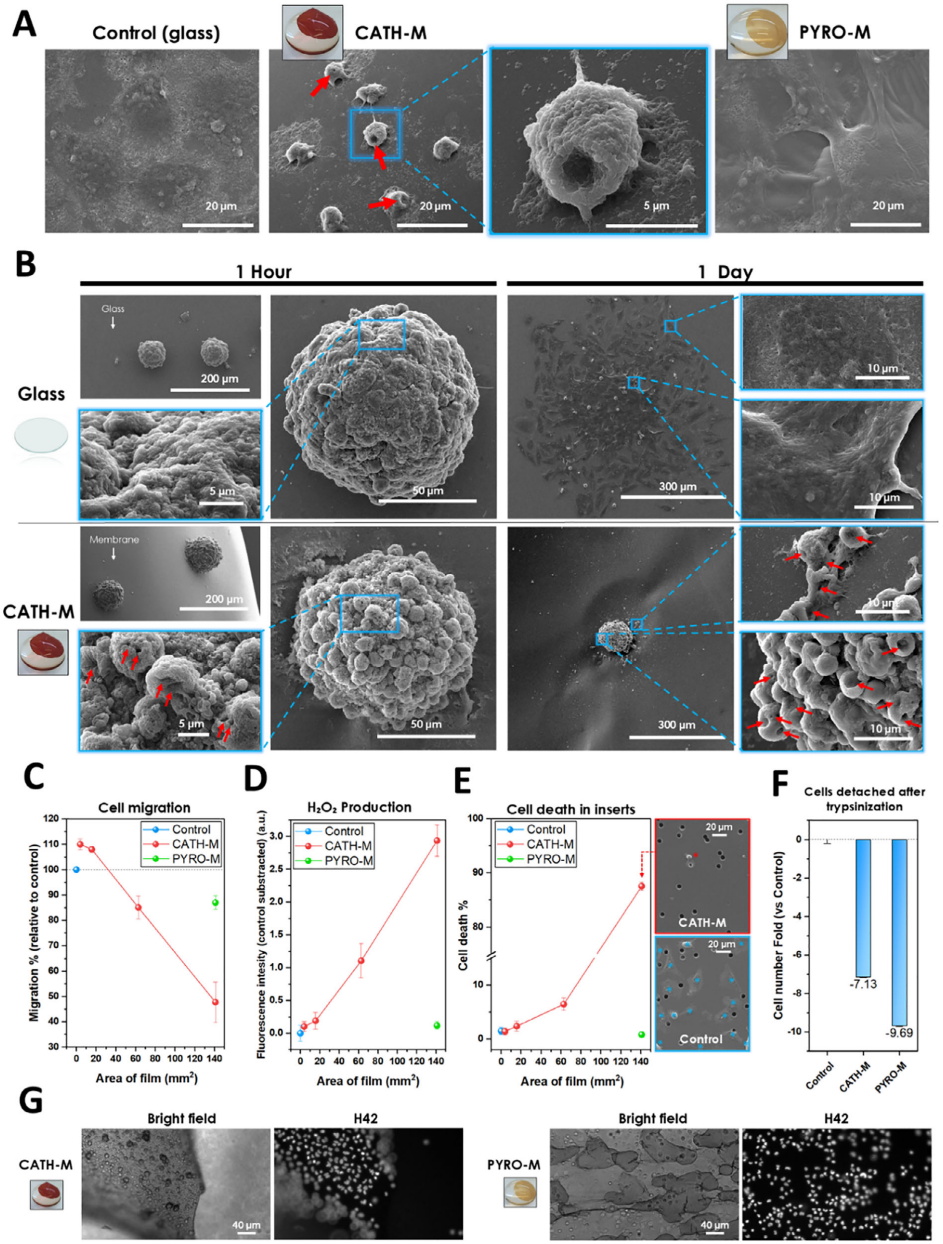
Glioblastoma cells adhesion and migration in the presence of **CATH‐M** membrane. (A) SEM micrographs of LN229 cells growing on glass substrates, **CATH‐M** or **PYRO‐M**. Considerable invaginations in the cell membrane can be observed in the case of **CATH‐M** (referenced with red arrows). (B) LN229 spheroids cultured on **CATH‐M** membrane, showing a substantial decrease in their invasiveness when compared to a glass substrate after 1 and 24 h, besides the change in the morphology of the cells that compose it (referenced with red arrows). (C) **CATH‐M** was capable to influence the LN229 cell migration in base of its amount (mm^2^) in the media, increasing it until a 110% or decreasing it below 50%. Data represented as the mean ± standard deviation, relative to control (3 independent tests with N = 3 each). (D) This decrease has shown to be inversely proportional to the H_2_O_2_ generated for the same amount of material (mean ± standard deviation, N = 3), and to be related with (E) the cell death % in the insert (mean ± standard deviation, N = 3). The SEM micrographs show the remaining cells in the bottom face of the inserts for the control and the highest amount of **CATH‐M**, marking each cell with a blue or red asterisk, respectively. (F) The trypsinisation of adhered LN229 cells to **CATH‐M** or **PYRO‐M**, compared to the bottom of a surface treated 96‐well plate, was considerably less efficient (approximately 7 and 9 times less, respectively). Indicated as mean ± standard deviation (N = 4) and expressed as fold change relative to the control. (G) Cells were able to remain homogeneously attached to the membrane after the trypsinisation. Error bars represent the standard deviation of a data set relative to the mean.

To investigate the effects of **CATH‐M** and **PYRO‐M** on LN229 cell behavior, we seeded 4 × 10^4^ cells/well (96‐well plate) onto glass (control) or the membranes, and incubated them for 24 h. Subsequent SEM analysis revealed that while LN229 cells exhibited normal morphology on control substrates and **PYRO‐M**, those seeded on **CATH‐M** displayed significant morphological alterations, characterized by invaginations, retraction, and signs of membrane damage (Figure 5A; Figure S25). Additionally, H42/PI staining confirmed that LN229 cells cultured on **CATH‐M** were dead after 24 h, while those on **PYRO‐M** remained viable (Figure S26).

For comparison, we analyzed the effect of **CATH‐M** on non‐tumoral healthy cells. For this, we cultured 5 × 10^5^ cells/well (24‐well plate) rat primary cortical astrocytes directly onto glass (control) or the membranes. Interestingly, SEM observations revealed that these cells were largely unaffected by **CATH‐M** and **PYRO‐M**, maintaining a morphology like the control (Figure S27). Additionally, H42/PI staining confirmed that astrocytes on **CATH‐M** and **PYRO‐M** remained viable after 24 h, (Figure S28). These results highlighted that **CATH‐M** has a specific targeting cytotoxic effect for tumoral cells, whereas healthy cells are unaffected, being able to further grow and proliferate.

To mimic a tumor‐like environment, both **CATH‐M** and **PYRO‐M** were tested in LN229 spheroids. For this, the spheroids were seeded on glass (control) or membranes and their evolution was monitored over 7 days using SEM and optical microscopy. Within the first hour, spheroids cultured on **CATH‐M** exhibited early signs of membrane damage, while those on **PYRO‐M** and glass remained largely unaffected (Figure 5B; Figure S29A). After 24 h, spheroids on glass and **PYRO‐M** demonstrated vigorous and uniform migration without signs of cell death (Figure S30). In contrast, spheroids on **CATH‐M** exhibited subtle, irregular migration, and a damaged spherical morphology, particularly in peripheral cells (Figure S29B). These differences became more pronounced after 3 days (Figure S31A). After 7 days, spheroids on glass and **PYRO‐M** had grown significantly, with **PYRO‐M** inducing a distinctive star‐like shape in the membrane (Figure S31B). Interestingly, spheroids on **CATH‐M** remained partially or completely intact, with limited migration and damaged cells at their edges. These findings indicated that **CATH‐M** significantly reduces the invasiveness of glioblastoma spheroids by inhibiting migration and inducing cell damage. This potent effect within a tumor‐like environment suggests a promising therapeutic mechanism for limiting disease progression.

#### 2.3.2 Cell Migration

Cell migration is one of the main mechanisms for tumor progression. To quantitatively assess the effect of **CATH‐M** on cell migration, we used cell culture inserts with LN229 cells seeded in the upper chamber and varying amounts of **CATH‐M** or **PYRO‐M** membranes introduced into the lower chamber as potential chemotactic stimulus (Figure S32). Interestingly, after 16 h of incubation, we observed that by increasing the amount of **CATH‐M** the migration decreased, while for lower concentrations enhanced the migration by up to 10% compared to the control, suggesting a chemoattractant effect (Figure 5C; Figure S33A). This chemoattractant effect could play an essential role as cell trap.

Given the hypothesis that **CATH‐M** might induce ROS production and considering the role of hydrogen peroxide (H_2_O_2_) in tumoral cell migration [50], H_2_O_2_ levels present in the media were measured after 16 h of incubation with **CATH‐M** membrane in the lower chamber. Interestingly, we observed a linear relationship between H_2_O_2_ generation and **CATH‐M** concentration, inversely correlated with migration, suggesting a potential link between the two (Figure 5D).

Finally, cell death in LN229 cells within the inserts after 16 h of incubation was quantified. While **PYRO‐M** did not induce cell death, **CATH‐M**, particularly at higher concentrations, significantly increased cell death, with a near 90% mortality rate at the highest membrane concentration (Figure 5E; Figure S33B). SEM analysis revealed that the majority of LN229 cells exposed to high amounts of **CATH‐M** exhibited a damaged spherical morphology and were predominantly located in the upper chamber of the insert, supporting the migration and viability results (Figure S34).

Overall, these observations suggest that, whereas **CATH‐M** can enhance LN229 cell migration under specific conditions, the observed decrease in migration at higher concentrations is likely due to increased cell death, where the generation of H_2_O_2_ seems to potentially contribute. The relationship between **CATH‐M** concentration and H_2_O_2_ production, coupled with cell migration, underscores the complex interplay between chemotactic stimuli and cellular responses. These findings highlight the dual role of **CATH‐M** in modulating cell behavior, where it can function as a chemoattractant at lower concentrations but induces cytotoxicity at higher levels. This duality is crucial for understanding the mechanisms underlying cell migration and developing therapeutic strategies targeting tumoral cell movement.

#### 2.3.3 Cell Adhesion

Most of the previous observed results could be influenced by the adhesion capacity of the developed membranes. Therefore, we compared the adhesion of LN229 cells to the bottom of a treated cell‐culture plate (control) with both **CATH‐M** and **PYRO‐M**. For this, cells were seeded and subsequently incubated for 3 h over the treated plastic (96‐well plate bottom) or membranes. After trypsinisation for 30 min, we quantified the detached cells in each condition (Figure 5F; Figure S35). Notably, the number of cells detached from membranes were at least seven times lower than from the control, even after protease treatment, demonstrating strong cell‐membrane adhesion (Figure 5G; Video S4). These results highlighted that both **CATH‐M** and **PYRO‐M** membranes, had significantly enhanced cell adhesion compared to the control. This strong adhesion is likely due to the broad interaction range of polyphenols and their exposed functional groups with the surrounding environment, making them highly effective in promoting cell‐membrane interactions.

### 2.4 Cell Energetic Metabolism Alterations

Mitochondrial damage plays a crucial role in this scenario, as it contributes to the generation of ROS, such as hydrogen peroxide. This oxidative stress can significantly impact cell migration and viability. Understanding mitochondrial damage is essential for elucidating the mechanisms underlying these cellular responses.

#### 2.4.1 Ultrastructural Analysis

**FIGURE 6:**
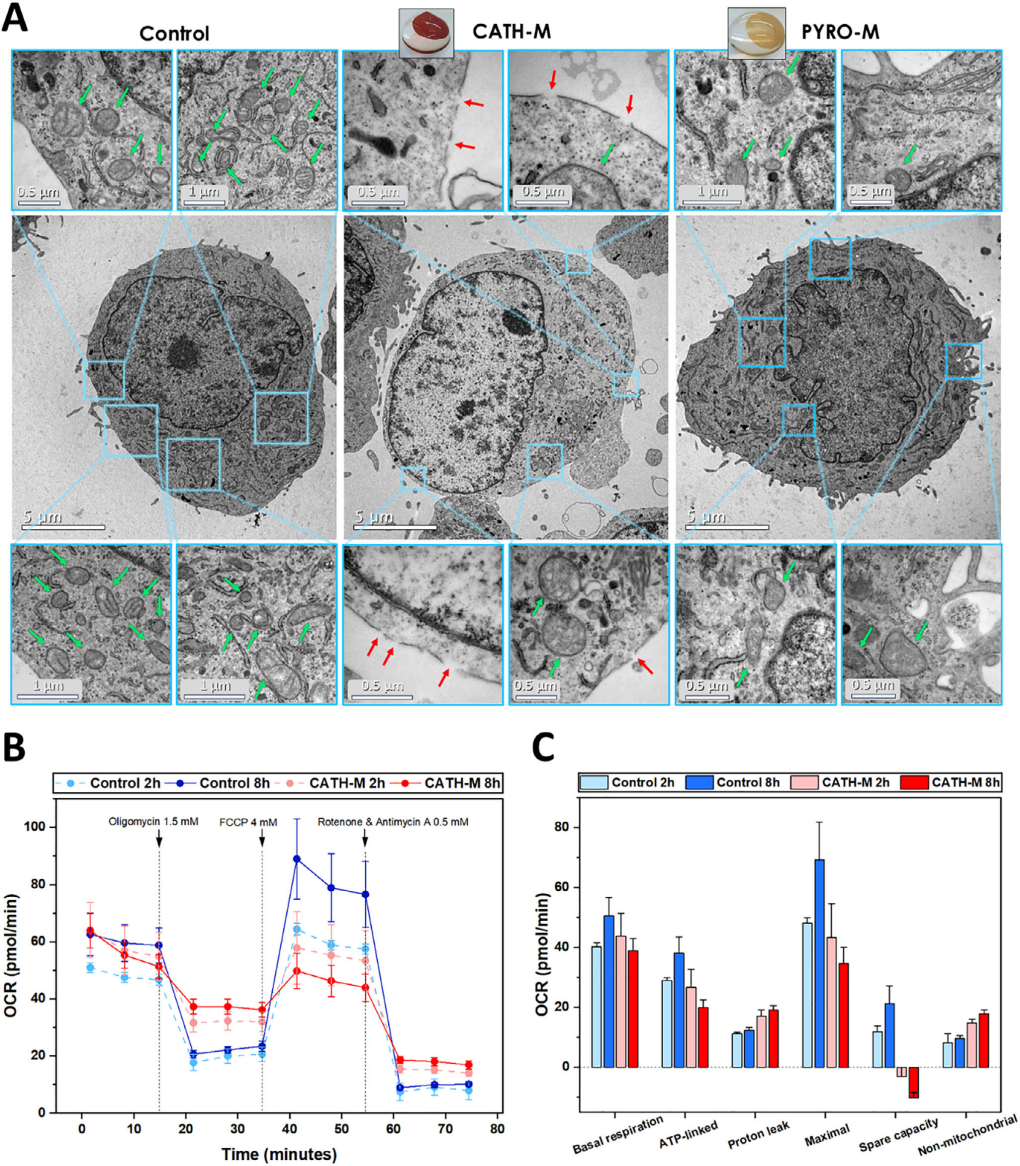
**CATH‐M** damages the mitochondria and its metabolic capacity. (A) Representative transmission electron microscopy (TEM) images of LN229 cells exposed to **CATH‐M** and **PYRO‐M** membrane. Green and red arrows indicate mitochondria and damage in the cell membrane (pores), respectively. (B) Oxygen consumption rate (OCR) profile of LN229 after being exposed to **CATH‐M** membrane for 2 and 8 h. (C) Individual respiration parameters obtained from the OCR profiles, where a decrease of the ATP‐linked respiration can be observed, suggesting a severe damage in the mitochondrial function. Error bars represent the standard deviation of a data set relative to the mean. Data was represented as the mean ± standard deviation for each condition, with the blank automatically subtracted (N = 3).

Transmission electron microscopy (TEM) analysis of LN229 cells treated with **CATH‐M** demonstrated severe mitochondrial damage, characterized by complete loss of cristae and decreased electron density, indicative of compromised mitochondrial matrix and intermembrane space integrity (Figure 6A). Besides, a decrease in the mitochondrial number, loss cytoplasmic material and permeabilization of the cell membrane (presence of pores) have been observed (Figure S36). While **PYRO‐M** treatment induced only minor mitochondrial damage, both **CATH‐M** and **PYRO‐M** treatments significantly upregulated autophagocytosis, as evidenced by increased vacuole and autophagosome formation (Figure 6A).

#### 2.4.2 Mitochondrial Function

To evaluate potential mitochondrial damage, a Seahorse assay was conducted to analyse the Oxygen Consumption Rate (OCR) and Extracellular Acidification Rate (ECAR) profiles of LN229 cells following exposure to **CATH‐M** for 2 and 8 h (Figure 6B). Whereas the control group exhibited an overall increase in OCR and ECAR from 2 to 8 h, indicative of cell growth, **CATH‐M** exposure resulted in a more linear profile, suggesting mitochondrial dysfunction. To accurately compare both conditions, individual respiration parameters derived from the OCR profiles were obtained (Figure 6C; Figure S37A).

In this sense, a crucial parameter related to mitochondrial activity is the ATP‐linked respiration. This parameter increased in the control group but decreased in **CATH‐M**‐exposed cells, particularly after 8 h, thus indicating mitochondrial dysfunction and impaired oxidative phosphorylation. Proton leak also moderately increased with **CATH‐M** exposure, reaching its higher value at 8 h, possibly due to mitochondrial membrane damage or as a protective mechanism against elevated ROS levels. Notably, in **CATH‐M**‐exposed cells, inhibition with oligomycin followed by carbonyl cyanide 4‐(trifluoromethoxy)phenylhydrazone (FCCP) failed to restore the OCR level of basal respiration (whereas the control group reached their maximum peaks), resulting in negative values of spare respiration, which suggested damage in the mitochondrial machinery. The increase in non‐mitochondrial respiration in **CATH‐M**‐exposed cells could be attributed to energy compensation mechanisms or non‐energetic processes such as stress‐related metabolism, inflammatory responses, or ROS‐mitigating enzyme activities like peroxidase.

Furthermore, ECAR analysis revealed a substantial decrease in **CATH‐M‐**exposed cells, indicating reduced glycolytic activity (Figure S37B). Additionally, the lower ECAR increase in **CATH‐M**‐exposed cells after oligomycin addition compared to the control suggests that the cells cannot compensate for energy deficiencies through enhanced glycolysis. Despite the absence of significant morphological changes in **CATH‐M**‐exposed cells after 8 h (Figure S37C), the observed decreases in OCR and ECAR parameters suggests that the cells may be losing their viability and energy synthesis capacity, potentially leading to eventual cell death when these parameters decline to a critical point.

### 2.5 ROS Production and Oxidative Damage

Given the redox properties of polyphenols, the presence of hydroxyl groups in the **CATH‐M**, and our previous findings, we hypothesized that ROS production plays a leading role in **CATH‐M** cytotoxicity. To assess this hypothesis, we conducted a comprehensive study of ROS generation.

#### 2.5.1 Antioxidant Protection and ROS Modulation

**FIGURE 7:**
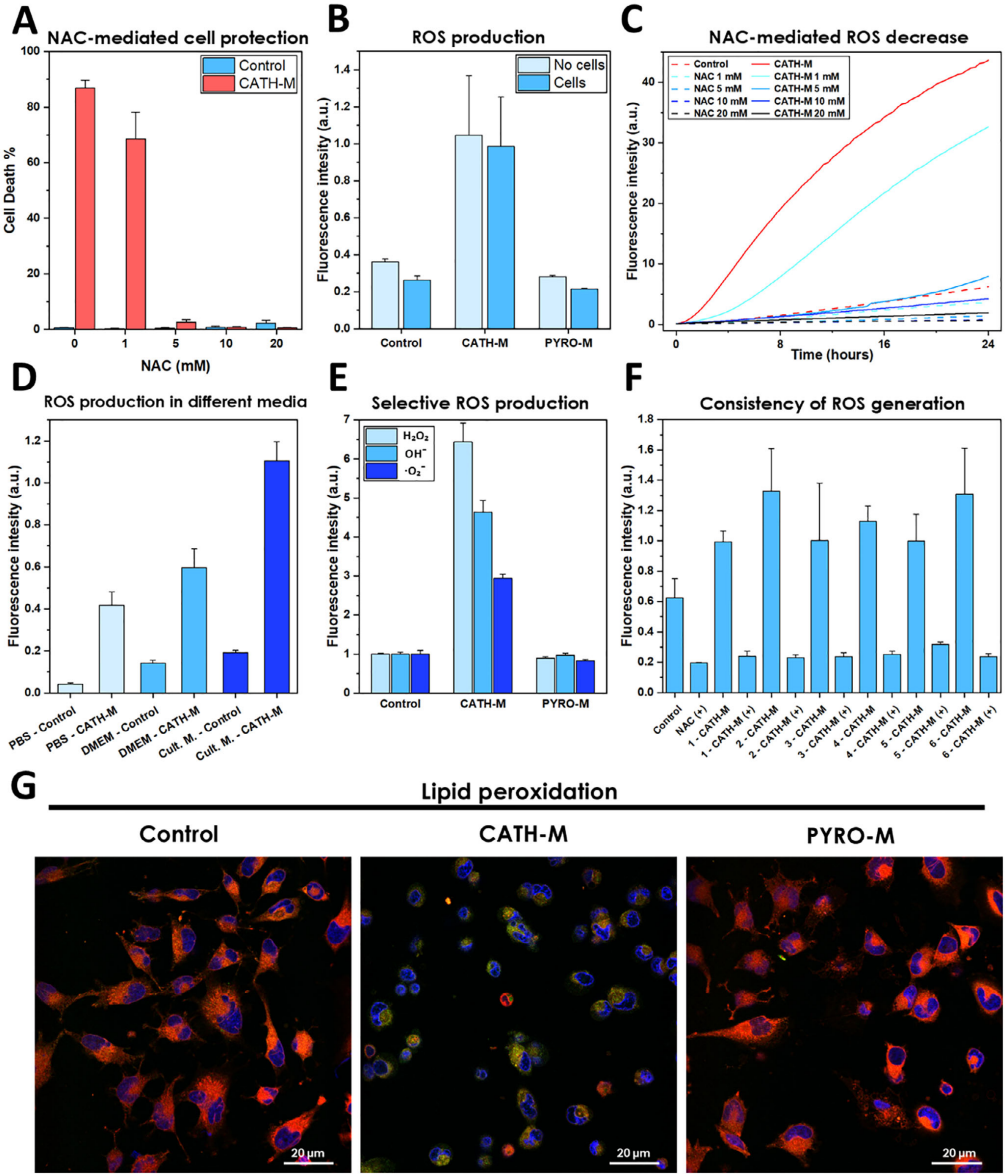
**CATH‐M** induces oxidative stress due to reactive oxygen species (ROS) production. (A) The antioxidant N‐acetylcysteine (NAC) shows a clear protective effect against **CATH‐M**, being capable to decrease completely the cell death induced by the membrane. Represented as the mean ± standard deviation (N = 5). (B) ROS production of **CATH‐M** after 24 h, in presence or not of LN229 cells. (C) The **CATH‐M** ROS generation in the media can be modulated in base of the concentration of NAC. (D) Changes in the ROS production of **CATH‐M** between phosphate‐buffered saline (PBS), Dulbecco's modified Eagle's medium (DMEM), and culture media. (E) Selective determination of ROS induced by **CATH‐M** and **PYRO‐M** membrane. Data was represented as mean ± standard deviation (N = 5), blank subtracted and expressed as fold change relative to the control. (F) Consistency in **CATH‐M** ROS generation and NAC inactivation between different synthesis. (G) Confocal images showing the lipid peroxidation of the plasmatic membrane induced by **CATH‐M** exposure (Blue = nuclei, RED = dye reduced‐state, Green = dye oxidized‐state). General ROS data was represented as mean ± standard deviation (N≥3).

To investigate the relationship between NAC concentration and protection against **CATH‐M**‐induced cytotoxicity, we treated 2 × 10^4^ LN229 cells with increasing concentrations of NAC (1‐20 mM) in the presence of **CATH‐M**. After 24 h, we observed a slight decrease in cell death with 1 mM NAC, a minimal cell death with 5 mM, and cell viability reaching control levels at 10 mM (Figure 7A). These results demonstrate that the cytotoxic effects of **CATH‐M** membrane can be modulated by the antioxidant NAC (Figure S38A).

Based on these results, ROS production by the **CATH‐M** and **PYRO‐M** was tested, both in the presence and absence of LN229 cells after 24 h of incubation. Whereas **PYRO‐M** did not produce detectable ROS, **CATH‐M** generated a strong signal, supporting the theory of oxidative cell damage (Figure 7B). Going a step forward, the generation of ROS in culture media was then measured every 20 min for 24 h, while simultaneously adding NAC at concentrations ranging from 1 to 20 mM. The ROS‐induced signal profile showed that **CATH‐M** acted as a consistent and steady generator of these species, rather than an abrupt burst (Figure 7C).

Additionally, increasing NAC concentrations proportionally decreased ROS levels and cell death, reinforcing the correlation between these parameters (Figure 7C).

To compare the cytotoxic effects of **CATH‐M** and H_2_O_2_, we treated LN229 cells with increasing concentrations of H_2_O_2_, ranging from 0.1 to 2 mM. After 24 h incubation, we found that cell death induced by 2 mM H_2_O_2_ was comparable to that induced by **CATH‐M**. This observation could suggest that, whereas the concentration of H_2_O_2_ and the amount of **CATH‐M** membrane may not directly correlate, their cytotoxic potential is similar (Figure S38B,C).

#### 2.5.2 ROS Capacity and Identification

To ascertain the effects of different environments on ROS generation by **CATH‐M**, we incubated **CATH‐M** in PBS, Dulbecco's modified Eagle's medium (DMEM), and culture media, for 1 h in absence (Figure 7D) or in presence of 2 × 10^4^ LN229 cells (Figure S39A). In both cases, we observed higher ROS production in culture media, followed by DMEM and PBS. We then expanded this analysis to include seven additional media used in the previous degradation assay, along with DMEM and FBS. After 1 h of incubation, we observed elevated ROS levels in PBS, DMEM, FBS, pH 10 solution, and ethanol (Figure S39E). However, after 24 h, only pH 10 solution and trypsin (the only media in which **CATH‐M** showed signs of degradation) maintained high ROS levels (Figure S39F).

To identify and quantify the specific ROS species generated by **CATH‐M**, we used selective kits to detect hydrogen peroxide and hydroxyl, and superoxide radicals. After 1 h of incubation with **CATH‐M** or **PYRO‐M**, in the absence (Figure 7E) or presence (Figure S39B) of 2 × 10^4^ LN229 cells, we found that only **CATH‐M** generated significantly higher signals for all three ROS species compared to the control.

To assess the robustness and reproducibility of **CATH‐M** properties, we studied six different **CATH‐M** membranes simultaneously in the absence or presence of 2 × 10^4^ LN299 cells, with or without the presence of NAC (5 mM). We observed that the signals achieved by the membranes, as well as their shifts after NAC‐induced inactivation, were strongly homogeneous and consistent (Figure 7F; Figure S39C). Finally, SEM analysis revealed that slight topographic changes in the membranes did not affect their ROS generation capacity (Figure S39D).

#### 2.5.3 Lipid Peroxidation

To shed more light on the oxidative stress and damage mechanism in the plasma membrane, cells were incubated with **CATH‐M** or **PYRO‐M,** and lipid peroxidation was assessed using a redox‐sensitive dye that shifts from red (reduced state) to green (oxidized state). Control and **PYRO‐M** showed a minimal color change, remaining predominantly red. Conversely, **CATH‐M**‐exposed cells exhibited a clear shift to green, almost completely losing the red color in the plasma membrane. These data further support the previously observed ROS production (Figure 7G; Video S5 and Figure S40).

### 2.6 Protein Profile Analysis

Assessing the protein profile of a new advanced material intended for medical applications is crucial. This evaluation helps ensure biocompatibility, minimize immune responses, and identify potential interactions with biological systems. By understanding the protein adsorption and interaction patterns, we can predict the material's behavior in the body and efficacy for medical use.

#### 2.6.1 Protein Arrays

**FIGURE 8:**
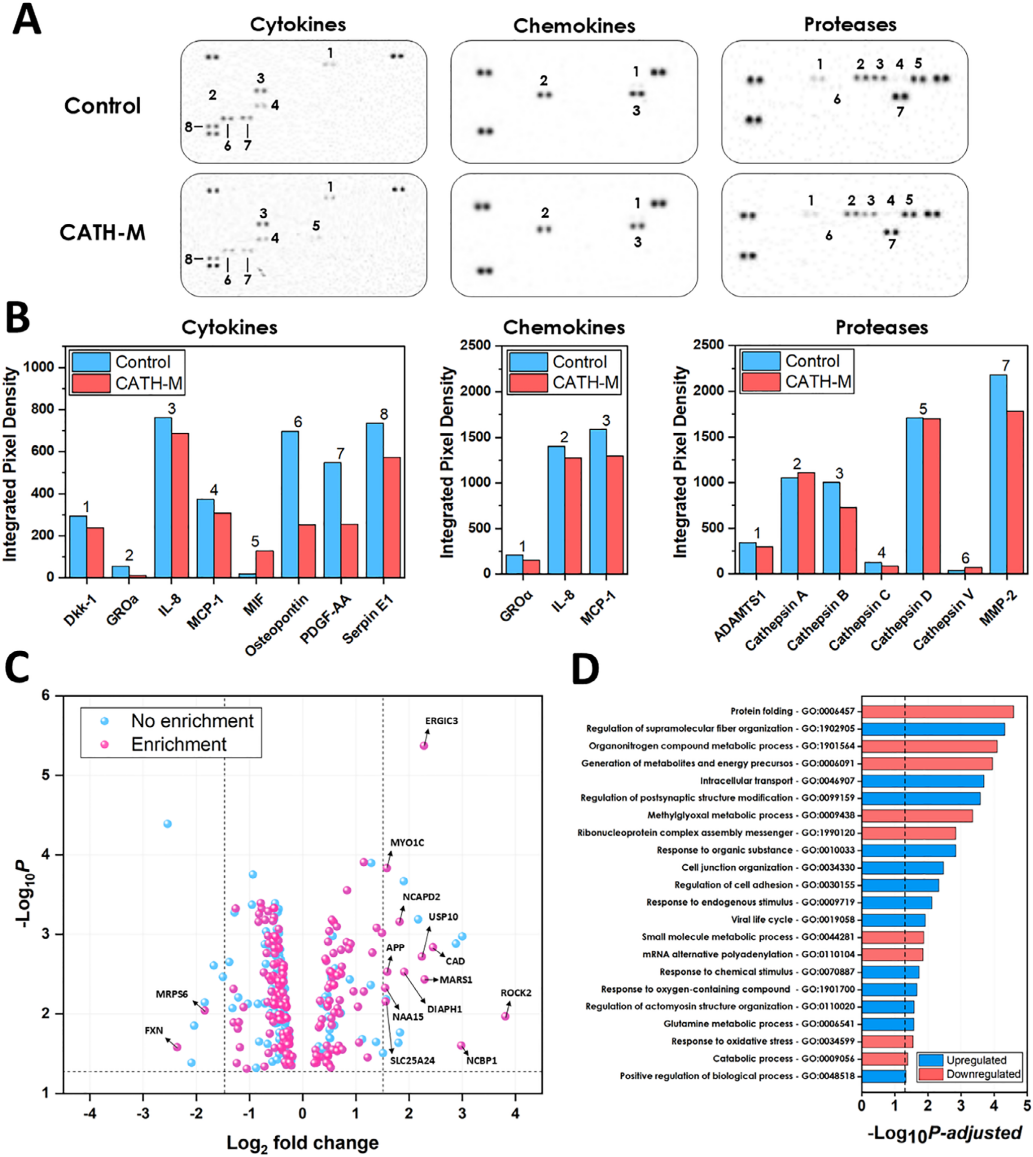
Alterations in the protein profile after **CATH‐M** exposure. (A) Cytokines, chemokines and proteases microarray signal obtained from the supernatant of LN299 cells after being exposed or not to **CATH‐M**. (B) Quantification of the mean signal obtained in each spot pair of each array for both conditions. Numbers above each spot pair are correlated with the shown over columns in each graph. (C) Volcano representation of the proteomic analysis results, showing the downregulated and upregulated genes after LN229 exposure to **CATH‐M**. Horizontal dashed lines represent a *p* value = 0.05. Blue and pink dots represent the genes observed before and after performing and enrichment analysis. (D) The different upregulated (blue) and downregulated (red) Biological Processes of the LN229 cells after being exposed to **CATH‐M**, showing their gen‐ontology (GO) number and their *P‐adjusted* value. Horizontal and vertical dashed line represent a *P‐adjusted* value = 0.05 and a fold change = 1.5, respectively.

To investigate the impact of **CATH‐M**‐induced cell death on human protein expression, a comprehensive proteomic analysis was conducted. Analysis of the supernatant revealed that cells exposed to **CATH‐M** did not express proteins that were absent under healthy conditions. However, **CATH‐M** exposure led to a reduction in the expression of several proteins involved in angiogenesis, including osteopontin, PDGF‐AA, and serpin E1 (Figure 8A,B). Conversely, proteins associated with cell survival under normal conditions were upregulated. Intracellular lysates analysis of **CATH‐M**‐exposed cells mirrored the general pattern observed in the supernatants, thus indicating that the membrane did not induce the expression of new proteins that were not previously expressed in healthy cells. Overall, **CATH‐M**‐treated cells responded by upregulating pre‐existing proteins involved in survival. This was evidenced by the protein arrays for cytokines, chemokines, proteases, ubiquitin‐involved proteins, and phospho‐immunoreceptors. Notably, protein expression in the ubiquitin array was the most prominent, whereas no signals were detected in the phospho‐immunoreceptor array for either the control or **CATH‐M** groups. (Figure S41).

#### 2.6.2 Proteomic Analysis

Exposure of LN229 cells to the **CATH‐M** membrane induced a series of responses compared to their respective controls, leading to a downregulation/upregulation of different genes (Figure 8C). These differentially expressed genes are involved in different biological processes, as categorized by Gene Ontology (GO) analysis (Figure 8D). Notably, we observed a downregulation of proteins involved in folding (GO:0006457), stress response (GO:1990120), and quality control mechanisms (GO:1901564). Additionally, it was also noticed a reduction in the cellular capacity to respond to oxidative stress (GO:0034599, GO:1901564), along with diminished protection against metabolic byproducts such as methylglyoxal (GO:0009438). Proteins encoded by genes essential for mitochondrial energy production and metabolic processes were also downregulated (GO:0006091, GO:1901564). Among all these findings, the downregulation of the protein encoded by the Frataxin gene was particularly notable (Figure 8C). This gene participates in the regulation of iron levels within mitochondria, preventing iron overload. Frataxin is also essential for the biosynthesis of iron‐sulphur clusters, which are important cofactors for various mitochondrial enzymes involved in metabolism and respiration. By regulating iron levels and contributing to iron‐sulphur cluster assembly, Frataxin helps to protect cells from oxidative damage.

On the other hand, an upregulation of the proteins involved in cytoskeleton regulation, lamellipodia formation, cell adhesion, migration and extracellular matrix formation, and remodelling was observed (GO:1902905, GO:0099159, GO:0034330, GO:0030155, GO:0110020). An increase of the proteins related to changes in cellular state in response to the presence of oxygen‐containing compounds was also identified (GO:1901700). Additionally, proteins related with the glutamine process (GO: 0006541) were also found upregulated. Nevertheless, their role as energy or intermediate metabolites providers for the tricarboxylic acid cycle, nucleotide, and amino acid biosynthesis, or contributing to redox homeostasis cannot be elucidated from this analysis. Finally, other upregulated proteins involved in membrane and vesicle trafficking, nuclear transport, protein sorting, and endocytosis, categorised under the intracellular transport (GO:0046907), were also identified. Among the upregulated proteins, the one encoded by ROCK2 was prominent (Figure 8C). ROCK2 regulates actin cytoskeleton dynamics, promoting actin filament stabilization and cell contractility, thereby affecting cell shape, migration, and morphology.

Overall, this proteomic analysis supports the **CATH‐M**‐induced changes in cellular shape, the responses to oxidative stress, and disruptions in mitochondrial function and energy production, as observed in the above experiments shown.

## 3 Conclusions

Our work introduces for the first time the use of novel bioinspired membranes designed as a potential patch platform for localized intervention in glioblastoma after surgery, representing a promising advancement in this field. Despite the rapid growth in mussel‐inspired research, this work is distinctly innovative. Unlike the common hydrogel and coatings focus, our solid, free‐standing bioadhesive membranes could offer a unique solution for glioblastoma treatment. Moreover, their selective toxicity against cancer cells, while sparing healthy astrocytes in vitro, highlights their potential for a novel and targeted application. Our bioinspired membrane addresses critical challenges such as adhesion in humid environments, infection prevention, and biocompatibility. The adhesive properties of the membrane ensure it stays in place, allowing for the sustained delivery of the localized cytotoxic effect to the affected area. This novel approach offers a localized and sustained therapeutic concept based on ROS modulation, that could complement the current glioblastoma treatment paradigm. However, the necessary next steps, establishing clinical protocols for implementing membranes and developing a corresponding reliable in vivo glioblastoma model, are essential and challenging prerequisites for in vivo testing of this novel material. Addressing these challenges will be the focus of future preclinical assays, following the development of a robust and reliable glioblastoma model in xenograft animals and the establishment of implementation and resection protocols.

## 4 Materials and Methods

All reagents, solvents, and kits were purchased and used without further purification from Sigma‐Aldrich (Merck, Madrid, Spain) unless otherwise specified. Type 1 ultrapure water from in‐house Milli‐Q filtration systems (Millipore, Burlington, MA) was used in all experiments, unless otherwise specified.

### 4.1 Synthesis of the Membranes

The synthesis of the membranes was performed after combining hexamethylenediamine (HMDA or ‐M) with different catechol derivatives: pyrocatechol (PYRO), caffeic acid (CAFF), pyrogallol (GALL), dopamine (DOPA), 4‐methylcatechol (4MET), or catechin (CATH), following the proportions (mM): **PYRO‐M** (50–75), **CAFF‐M** (75–150), **GALL‐M** (100‐200), **DOPA‐M** (50‐125), **4MET‐M** (100‐150) and **CATH‐M** (50‐125). The selected catechol derivative and its corresponding amount of HMDA were weighted separately and dissolved in a final volume of 50 mL of water. Both reagents where mixed and poured in a sterile 15 cm diameter polypropylene Petri dish. In the case of **CATH‐M** film, CATH was dissolved in 45 mL of sterile water, sonicated, and heated. An autoclave‐sterilized 15 cm glass dish was used for the membrane synthesis. The oxygenation of the reaction was regulated by opening holes in the corresponding lids: parafilm sealing and 1 hole in plastic lid (**PYRO‐M**, **CAFF‐M**, **GALL‐M**, and **4MET‐M**), parafilm sealing and 4 holes in the plastic lid (**DOPA‐M**), or no parafilm with a hole in a lid (**CATH‐M**). The synthesis was performed for 48 h at 25°C. Membranes were obtained the interface between the air/water interface. Afterwards, the membranes were removed from their plates, washed three times with 15 mL of sterile water (until water was clean), and then stored in 10 cm sterile Petri dish until use. In each case, membranes were washed and cut to the final size inside 15 mL phosphate buffered saline (PBS) or saline solution (NaCl 0.9%) right before exposing them to cells or bacteria, respectively. All procedures were performed under biosafety level 2 laminar flow cabins to ensure sterile conditions.

### 4.2 Fourier transformed Infra‐Red (FTIR)

Surface FTIR experiments have been performed with the Hyperion 2000 FTIR microspectrometer (Bruker Optik GmbH, Ettlingen, Germany) in reflection mode, equipped with a nitrogen‐cooled mercury cadmium telluride (MCT) detector (InfraRed Associates, Inc., Stuart, FL, USA), using a 15 × reflection objective, a gold mirror as a reference and scanning for 30 min with a resolution of 4 cm^−1^. The membranes were attached on a gold substrate. All the data was treated with OPUS version 7.0 (Bruker) and OriginPro (version 8.0988, OriginLab Corporation, Northampton, MA, USA) software.

### 4.3 X‐Ray Photoelectron Spectroscopy (XPS)

XPS measurements were performed with a Phoibos 150 analyser (SPECS EAS10P GmbH, Berlin, Germany) in ultra‐high vacuum conditions (based pressure 10^−10^ mbar, residual pressure around 10^−7^ mbar). Monochromatic Al Kα line was used as X‐ray source (1486.6 eV and 300 W). The electron energy analyser was operated with pass energy of 50 eV. The hemispherical analyser was located perpendicular to the sample surface. The data was collected every eV with a dwell time of 0.5 s. A flood gun of electrons, with energy lower than 20 eV, was used to compensate the charge. The reference was set for C‐C at 284.8 eV All the data was treated with CasaXPS version 2.3.17PR1.1 (Casa Software LTD, Teignmouth, UK) [51] and OriginPro software.

### 4.4 Elemental Analysis

The membrane composition was determined through elemental analysis conducted by the services at the Parque Científico Tecnológico of the Universidad de Burgos, using an EA Flash 2000 with a TCD detector and a Mettler Toledo XP6 microbalance. Carbon, hydrogen, oxygen and nitrogen molar percentages were acquired for each HMDA‐based membrane. Results were expressed as the mean ± standard deviation from 3 different membranes (N = 3).

### 4.5 Degradation Test

The stability of the **CATH‐M** and **PYRO‐M** membranes was evaluated in water, ethanol 100%, pH 4 solution (HCl/sodium acetate), pH 10 solution (Na_2_CO_3_/NaHCO_3_), PBS, cell culture media (see next section) or trypsin (0.25% trypsin and 1 mM EDTA). Five mL of each solution were added to sterile 20 mL glass vials containing 2 discs of **CATH‐M** or **PYRO‐M** membranes. The degradation tests were performed at 37°C for 1, 2, 3, 4, and 8 months. After each time point, the membranes were removed, washed in water and further characterized by SEM.

### 4.6 Scanning Electron Microscopy (SEM)

The morphology and thickness of the membranes was determined by scanning electron microscopy (SEM, FEI Quanta 650 FEG, Thermo Fisher Scientific, Eindhoven, The Netherlands) in secondary electron mode with a beam voltage of 10 kV. Samples were coated with a 5 nm layer of gold/palladium 60/40 layer (Emitech K550X Sputter Coater) before the observation. In the case of membranes and substrates containing cells, after fixation with 2% paraformaldehyde (PFA) in PBS for 1 h, samples were dehydrated through ethanol serial dilution series until 100%. Afterwards, samples were dried under cabin airflow and coated with a 5 nm layer of gold/palladium.

### 4.7 Nanotensile Monotone Traction Test

The mechanical properties of **CATH‐z** were evaluated using a monotone uniaxial tensile test. Testing was performed on an Agilent T150 Nanotensile System (Agilent Technologies, Santa Clara, CA) equipped with a 500 mN load cell at the Bio/NanoMechanics for Medical Sciences Laboratory, ATeN‐Center, Palermo (Italy). A monotone uniaxial tensile test was performed to determine the elastic modulus and elongation at break. The **CATH‐M** samples, with dimensions of 7 cm length, 1 cm width, and 2.1 µm thickness, were glued to a paper holder and mounted in the machine grips. Data was acquired for load (mN) vs. extension (mm), and time (s). The stress‐strain curve was analyzed to determine engineering stress (σ) and engineering strain (ε) defined as $\sigma =\frac{F}{S}$, where F is the applied force and S is the cross‐sectional area of the sample, and $\varepsilon \;=\frac{L-L_{0}}{L_{0}}\;$, where L0 is the original length (Nominal Gage Length input data) and L is the extended length of the sample. The shear adhesion force was assessed using a uniaxial shear/sliding configuration with the same nanotensile system. The contact area between the membrane and the biological substrate was 1 cm^2^ (1 cm × 1 cm). A displacement‐controlled sliding mode was applied, and data for load (mN) vs. extension (mm) and time (s) were recorded. The adhesion force profile was determined by applying increasing displacement for 30 min to observe the dynamic behavior. All tests were conducted in an isolated cabinet on an anti‐vibration table to ensure measurement accuracy.

### 4.8 Sample Preparation for TEM

Initially 3 × 10^6^ LN229 cells were homogenously seeded in a 100 mm Petri dish in a final volume of 10 mL of culture media. After 24 h incubation at 37°C, media was changed for 12 mL of fresh culture media and an 8 cm diameter disc of **CATH‐M**, **PYRO‐M** was introduced in the Petri dish, or not (control). After 8–10 h incubation, membranes were carefully removed and media removed. After following the trypsinisation protocol, a cell pellet was obtained and fixed with glutaraldehyde 2.5% and PFA 2% in PBS. Then, Sample Preparation Service of *Universitat Autònoma de Barcelona* Microscopy Unit obtained microtomes for each condition. Finally, samples were observed by TEM (Hitachi H‐7000 with CCD GATAN ES500W Camera).

### 4.9 Cell Culture

Glioblastoma cell lines were routinely cultured and maintained in 100 mm cell culture dishes (Falcon 353003, Fisher Scientific), using 10 mL of Dulbecco's modified Eagle's medium (DMEM), supplemented with 100 µg/mL streptomycin, 100 U/mL penicillin, and 10% heat‐inactivated fetal bovine serum (FBS) (Invitrogen), from now on culture media. Cells were incubated at 37°C in a saturating humidity atmosphere composed of 95% air and 5% CO_2_ (Fisherbrand CO_2_ Incubator Isotemp). After achieving 85–90% of confluence, cells were rinsed with PBS (100 mM, pH 7.4) and incubated at 37°C for 3 min with trypsin (0.25% trypsin and 1 mM EDTA) until dissociated. Afterwards, culture media was added to neutralize trypsin and the resulting suspension was centrifuged at 200 g for 5 min. Finally, the pellet was resuspended in culture media and, unless otherwise indicated, the cell density was adequately adjusted to conduct each experiment. The LN229 huma glioblastoma‐derived cells (ATCC CRL‐2611, 2010) were tested during entire study. Besides, four commercial cell lines (LN18 (ATCC CRL‐2610, 2010)), A172 (ATCC CRL‐1620, 2010), U87‐MG (ATCC HTB‐14, 2010) and U251‐MG (ECACC 09063001, 2013)), two primary cultures (MSO4 and MSO7) and rat primary cortical astrocytes. The patient‐derived glioblastoma cells MSO4 and MSO7 were isolated from tumor samples provided by Hospital Universitari de Bellvitge‐ICO Duran i Reynals as previously reported [52, 53]. They are crucial because established commercial glioblastoma cell lines often differ significantly from in vivo tumors in terms of genetic mutations, epigenetic profiles and drug sensitivity. Primary cultures maintain more of the heterogeneity and specific characteristics of the original patient tumor, making them more physiologically relevant for studying glioblastoma biology, drug response and developing patient‐specific therapies. Their use allows for the study of inter‐patient variability.

Primary cultures of cortical astrocytes were prepared from 1‐day‐old (P1) Sprague–Dawley rats (mixed sex) following a previously established protocol [54]. All animal procedures adhered to institutional guidelines and ethical regulations regarding animal care and use. Cells were plated into 10 mm dishes at a density of 0.3 × 10^6^ viable cells/mL in DMEM supplemented with 10% fetal bovine serum (FBS), 50 units/mL penicillin and 50 µg/mL streptomycin. The cultures were incubated at 37°C in a humidified atmosphere of 5% CO_2_/95% air and used at 21 days in vitro. These cells are fundamental for studying normal brain function. They are essential for understanding the healthy nervous system context, which can then be compared to pathological conditions (like glioblastoma interactions). Using rat astrocytes specifically is common for in vitro modeling due to ease of isolation and established protocols, often serving as representative healthy nervous system cells.

A549 (ATCC CCL‐185, 2015), PANC‐1 (ATCC CRL‐1469, 2015), CACO‐2 (ATCC HTB‐37, 2024), SAOS‐2 (ATCC HTB‐85, 2007) and HELA (ATCC CCL‐2, 2012), were selected as representative tumoral cell lines for lungs, pancreas, colorectal, bone and cervical cancer, respectively.

For commercial cell lines, ATCC and ECACC routinely performs quality control testing for mycoplasma (PCR‐based) and other microbial contamination (bacteria, fungi) for all cell lines prior to distribution. Additionally, for all commercial cell lines and primary cultures, regular contamination checks (i.e., mycoplasma PCR and bacterial/fungal cultures) were performed. In all the cases the cells were confirmed to be free of such contamination during the study.

To irradiate LN229 cells, plates containing them were brought to the “*Servei de Radiacions Ionitzants (UAB)*” and exposed to 10 or 20 greys (Gy).

All procedures were performed in a biosafety level 2 laminar flow cabins in sterile conditions. These procedures adhered strictly to the specific approved protocols established by the Ethical and Biosafety Committee of the *Universitat Autònoma de Barcelona*, as well as governmental authorities (protocol numbers: A/ES/20/I‐40 and A/ES/20/86).

### 4.10 Cell Viability

A double staining was used to evaluate the cytotoxic effect of the treatments performed in a 96‐well plate for all the cells (Falcon 353072, Fisher Scientific) and in a 24‐well plate in the case of astrocytes (Falcon 353047, Fisher Scientific). After the desired incubation time of the cells and the 6 mm diameter membrane disc in 50 µl of culture media (15 mm diameter and 500 µl for astrocytes), 50 µl more of culture media with Propidium Iodide (PI, 1 µg/mL) and Hoechst 33342 (H42, 2 µg/mL) were added to each well (500 µl for astrocytes) without removing the membranes (unless otherwise specified). Then, plates were incubated at room temperature protected from light for 30 min. H42 can pass through intact cell membranes, but PI can only enter cells that have suffered membrane damage. Finally, samples were examined under a Nikon ECLIPSE TE2000‐E microscope, equipped with epifluorescence optics for visualizing fluorescent signals and a Hamamatsu ORCA‐ER photographic camera for capturing high‐quality images. Images were collected for each dye and bright field at various locations for each experimental condition. Cell death percentage was quantified as the percentage of cells with damaged membranes (PI‐positive) relative to the total number of cells (H42‐positive).

### 4.11 Clonogenic Assay

LN‐229 cells were seeded at an initial density of 2.5 × 10^5^ cells/well in 9 of 12 wells within a 12‐well plate (Falcon 353003, Fisher Scientific) using a total volume of 1 mL of culture media. After a 24‐h incubation period at 37°C, the media was replaced with 0.75 mL of fresh culture media, and 21 mm diameter discs cut from either the **CATH‐M** or **PYRO‐M** membrane were carefully introduced into each well, with control wells receiving no membrane (Treatment control). Additionally, 1.0 × 10^3^ LN229 cells were seeded in the remaining three empty wells to serve as a long‐period growth control. Membranes and cells were incubated for an additional 24 h. Subsequently, the membranes were removed, each well was washed with PBS, and crystal violet staining was performed in one plate to represent day 0 (T0). In the remaining four plates, after membrane removal and media removal, 1 mL of filtered conditioned medium (50% fresh medium and 50% medium from a confluent LN229 plate) was added to each well. The culture medium was refreshed weekly with fresh medium, and cell growth was monitored until day 20 (T20). At the end of the experiment, all four plates were stained with crystal violet. A total of three replicates were performed for each condition at T0, while twelve replicates were performed for each condition at T20. To perform the crystal violet staining, culture media was removed from the well, followed by a washing step with PBS. Then, 100% methanol at −20°C was added to each well, and the plate was incubated at −20°C for 10 min. Afterwards, the methanol was removed, and crystal violet solution (0.5 g in 100 mL of 25% methanol) was added to the wells, where it remained for 10 min. Finally, the dye was removed, washing the plate generously with distillate water to eliminate the unattached colorant. The plate was then allowed to dry completely before imaging the results. To quantify the amount of dye retained in each well, 1 mL of 15% acetic acid in PBS was added to dissolve the dye, and the absorbance of the 10^−1^ dilution was measured at 595 nm using a Varioskan LUX microplate reader (Thermo Scientific).

### 4.12 Local Cytotoxic Effect

Initially, 3 × 10^6^ LN229 cells were homogenously seeded in a 100 mm Petri dish in a final volume of 10 mL of culture media. After 24 h, a piece of CATH‐M or PYRO‐M membrane was cut to cover only half of the plate. Then, membranes were introduced in the plates containing LN229, fixed their location with needles and incubated for 24 h. Finally, the membranes were carefully removed, and cells were stained with crystal violet following the previously described protocol.

### 4.13 Glioblastoma Spheroids

From a LN229 cell suspension, 3 × 10^6^ cells were homogenously seeded in a 100 mm non‐treated Petri dish in a final volume of 12 mL of culture media, changing it every 5 days. After reaching a desired spheroid size, the plate was gently hit to detach them. Then, the suspension containing the spheroids was carefully recollected, letting them precipitate for 5 min and removing unnecessary supernatant. Previously obtained LN229 spheroids with an approximated size of 80–100 µm were used. In four 12‐well plate, 1 mL of culture media was added to each well, as well as **CATH‐M** or **PYRO‐M** membrane, a circular 21 mm glass cover slide (SEM Control), or nothing (Control), having in each plate N = 3 for each condition. Subsequently, 20–40 spheroids/well were seeded and kept incubating for 1 h and 1, 3 and 7 days (each time corresponding to a specific 12‐plate). Bright field images were obtained in each incubation time, followed by a PFA 2% fixation for 1 h. **CATH‐M** and **PYRO‐M** membranes, as well as glass cover slides, containing fixed spheroids were observed by SEM.

### 4.14 Cell Migration

To determine the influence of the membranes in the migration of the cells, 8 µm pore diameter inserts were used (Falcon 353097, Fisher Scientific). The corelation between migration, H_2_O_2_ production and survivability was assessed. First, for migration, a 24 well plate (Falcon 353047, Fisher Scientific) was filled with 800 µl of culture media, then no discs (control), 5 discs of 1, 2, 4 or 6 mm diameter of **CATH‐M**, or 5 discs of 6 mm diameter of **PYRO‐M** were added and remained incubating at 37° for 6 h. Subsequently, the insert was introduced in the well (containing or not membranes) and seeding 200 µl of culture media with 4 × 10^4^ cells over the inserts. After incubating for 16 h at 37°C, the cells that migrated to the lower face of the insert were quantified. To achieve that, cells were fixed with PFA at 4% final for 1 h, followed by the removal of the attached cells in the upper part of the insert (non‐migrated cells) with a cotton swab. Afterwards, inserts were generously washed, stained with H42 in PBS and observed by fluorescence microscopy the remaining cells in the lower face of the insert (migrated cells). Three independent tests, with N = 3 each one, were performed. The H_2_O_2_ production in the lower chamber was measured after the 16 h of incubation with the inserts and the membranes using the kit of OxiVision Green hydrogen peroxide sensor (see next section). Finally, to determine the survivability, after the 16 h of incubation with the inserts and the membranes, culture media with H42 and PI was added to both sides of the insert, incubated at room temperature for 30 min to then determine the cell death percentage off all the cells in the inserts, migrated plus non‐migrated (N = 3). Right after, cells were fixed with PFA 2% in PBS for 1 h. Then, the insert's membranes containing the cells were removed, prepared and observed by SEM both the upper and lower face.

### 4.15 Cell Adhesion

In a 96 well plate, 100 µl/well of culture media were added, inserting a **CATH‐M** or **PYRO‐M** membrane, or not (control). Subsequently, 100 µl with 4 × 10^4^ LN229 cells were seeded in each well. After 3 h incubation at 37°C, media was carefully aspirated and 200 µl of 1/5 of Trypsin in PBS were added and subsequently incubated for 30 min. Then, the plate was consistently hit to detach the affected cells, moving the supernatant to a new well (detached cells) and the membranes to another one (still attached cells). Besides, 100 µl of culture media with H42 (2 µg/mL final) were added to both cases to neutralize the Trypsin and to visualize the cells by fluorescence (N = 4).

### 4.16 Mitochondrial Function

The alteration of the oxygen consumption rate (OCR) after exposing **CATH‐M** membrane to LN229 cell line was performed with the Seahorse XF Cell Mito Stress Test Kit (Agilent). First, 1.3 × 10^4^ cells were seeded in a final volume of 100 µl of culture media in 6 wells of an 8‐well seahorse plate (XF HS miniplate 103725‐100), while 100 µl without cells in the remaining 2 wells (blank), followed by overnight incubation at 37°C. Then, media was changed for 50 µl of fresh culture media and a 4 mm **CATH‐M** membrane disc was introduced in each of the wells already containing cells, or not (control). After 2 and 8 h incubation at 37°C, membranes were removed and media was exchanged for 180 µl of XF DMEM medium (Agilent), enriched with Pyruvate (1 mM), Glutamine (2 mM) and Glucose (10 mM), followed by 45 min incubation at 37°C in a non‐CO_2_ incubator. Then, the assay started and oligomycin 1.5 mM, FCCP 4 mM and rotenone/antimycin A 0.5 mM at final concentrations were used for each cycle (Seahorse XF HS Mini Analyzer, Agilent). For each incubation time, N = 3 was obtained.

### 4.17 Antioxidant Protection Assays

The antioxidant NAC was used to demonstrate and regulate the oxidative effect induce by **CATH‐M** membrane. First, 2 × 10^4^ LN229 cells/well were seeded in a 96 well plate, followed by a 24 h incubation at 37°C. Then, media was changed for 50 µl of fresh culture media, containing 1, 5, 10 or 20 mM of NAC, or not (control). Besides, a **CATH‐M** 6 mm disc membrane was added to each well or not in base of the condition (N = 5). Finally, after 24 h incubation at 37°C, cell death percentage was quantified.

Furthermore, the H_2_O_2_ tolerance of the LN229 was also tested following a similar procedure. In this case, media was changed for 50 µl of fresh culture media containing 0, 0.1, 0.25, 0.5, 1 or 2 mM H_2_O_2_, as well as only culture media with a 6 mm disc **CATH‐M** or **PYRO‐M** membrane (N = 5). Therefore, after 24 h incubation at 37°C, cell death percentage was also quantified.

### 4.18 ROS Determination

The reactive oxygen species (ROS) production of the **CATH‐M** membrane was assessed with the MAK143 Fluorometric ROS KIT. Initially, 2 × 10^4^ LN229 cells/well were seeded in a 96 well plate, followed by a 24 h incubation at 37°C. Then, media was changed for 50 µl of fresh culture media (or other solvent) and a 6 mm **CATH‐M** or **PYRO‐M** disc was introduced in the well, or not (control). In case of measuring in absence of cells, membranes were introduced directly in 50 µl the specific solvent. After the desired incubation time at 37°C, the fluorescence intensity inside the wells was measured after 30 min of incubation with the kit (Varioskan LUX, Thermo Scientific and λ_excitation_ = 490 nm / λ_emission_ = 525 nm for excitation and emission wavelength, respectively) in at least 3 repetitions. The selective determination of ROS was performed with three kits, OxiVision Green hydrogen peroxide sensor (Kit A), MitoROS OH580 (Kit B) and MitoROS 580 (Kit C), in order to detect hydrogen peroxide (H_2_O_2_), hydroxyl radicals (^⋅^OH) and superoxide anions (^⋅^O_2_^−^), respectively (AAT Bioquest, US, California). Kit A: after preparing the working‐solution at 20 µM on 20 mM Hepes buffer in PBS, 50 ul were added to each well; Kit B: after obtaining a 2X working‐solution of the assay buffer, 50 µl were added to each condition; Kit C: after achieving a 2X working‐solution in 20 mM Hepes buffer in PBS, 50 µl were added to each well. All the kits were prepared simultaneously and incubated for 30 min at room temperature and protected from light after adding them. Right after, KIT A was read at λ_excitation_ = 490 nm/ λ_emission_ = 525 nm, while Kits B and C at λ_excitation_ = 510 nm/λ_emission_ = 580 nm. For each Kit/condition N = 5 was performed.

### 4.19 Lipid Peroxidation

To observe the effect of ROS in the plasmatic membrane, the Image‐iT Lipid Peroxidation Kit (ThermoFisher Scientific) was selected. Upon oxidation in live cells, fluorescence shifts from red (reduced dye λ_excitation_ = 581 nm/ λ_emission_ = 590 nm) to green (oxidized dye is λ_excitation_ = 488 nm/ λ_emission_ = 510 nm). First, 200 µl of culture media are added in each well of a u‐slide 8 well plate (Ibidi 80807), followed by 300 µl of culture media containing 7 × 10^4^ LN229 cells. After being incubated overnight, media was changed for 250 µl of culture media with 15 µM Image‐iT sensor, followed by additional 30 min incubating at 37°C. Then, media was removed and changed for 200 µl of fresh one, adding to them two 8 mm discs of **CATH‐M** or **PYRO‐M** membrane, or not (control). The plate remained incubating for additional 3 h at 37°C. Finally, three washes with PBS were performed, adding in the last one H42 (nuclei staining), and samples were observed by confocal microscopy (Leica SP5). Images were 3D rendered with Blender 4.2.

### 4.20 Bacteria Growth Conditions

*Escherichia coli* (*E. coli*, MG 1655) and Methicillin‐resistant *Staphylococcus aureus* (MRSA, CECT 9951) were selected as representative gram‐negative and gram‐positive bacteria, respectively. The selection of these strains was mainly due to their impact in current human health and relevance in research stated by the WHO and UN and their implication in brain surgery's infection. Initially, an aliquot from the bacteria was streaked on a 100 mm Petri dish containing Miller's Luria‐Bertani (LB) with agar and incubated for 24 h at 37°C (Forma Series II Water‐Jacketed CO_2_ Incubator) under a saturating humidity atmosphere composed of 95% air and 5% CO_2_. For each experiment, a single colony was selected and grown in 10 mL of Miller's LB for 24 h at 37°C. Bacteria cultures were resuspended in NaCl saline solution at 0.9% and the optical density (OD_600_) of the suspension was adjusted (Fisherbrand Cell Density Meter Model 40) to 0.2 for *E. coli*, and 0.3 for MRSA. In every case, 100 mm Petri dish plates with Miller's LB with agar were used for bacteria growth and antibacterial tests. All procedures were performed under biosafety level 2 laminar flow cabins and sterile conditions. These procedures adhered strictly to the specific approved protocols established by the Ethical and Biosafety Committee of the *Universitat Autònoma de Barcelona*, as well as governmental authorities (protocol numbers: A/ES/18/I‐12 and HR676‐21).

### 4.21 Antibacterial Properties in Suspension

After obtaining the respective OD600 for each bacteria strain, 50 µl of suspension was added per well in a 96 well plates (Falcon 353072, Fisher Scientific), introducing inside of them a disc of 6 mm of **CATH‐M**, **PYRO‐M**, or nothing as control, followed by incubation at 37° for 24 h. Subsequently, the plate was sonicated for 1 min (Elmasonic S 30H) and serial dilutions were performed. Plates were seeded following the already described methodology for single plate‐serial dilution spotting (SP‐SDS) [55] and kept incubating at 37°C for 24 h. Finally, viable colony‐forming units (CFU) were counted afterwards, with a total number of repetitions of N = 12 for each condition. To determine ROS generation in base of bacteria/membrane selected, after the 24 h incubation, 50 µl of MAK143 kit was added to each well and incubated at room temperature for 30 min to finally read the fluorescence intensity (N = 6) (Varioskan LUX, Thermo Scientific and λ_excitation_ = 490 nm/λ_emission_ = 525 nm).

### 4.22 Protein Arrays

LN229 cells (3 × 10^6^) were distributed in a 100 mm Petri dish containing 10 mL of culture media. Following a 24‐h incubation at 37°C, the medium was replaced with 12 mL of fresh culture media, and an 8 cm diameter disc of **CATH‐M** was introduced into the dish, or the dish remained as a control. After an additional 8–10 h, the membrane was carefully removed, and the medium was collected. Cells were trypsinised, pelleted, and washed with PBS. Subsequently, lysates or supernatants from each condition were analysed using five distinct microarrays: the Proteome Profiler Human XL Cytokine, Chemokine, Protease, Ubiquitin, and Phospho‐Immunoreceptor Array Kits (R&D Systems).

### 4.23 Proteomic Analysis by Liquid Chromatography with Mass Spectrometry (LC‐MS/MS)

LN229 cells (3 × 10^6^) were homogenously seeded in a 100 mm Petri dish containing 10 mL of culture media. After 24 h incubation at 37°C, media was changed for 12 mL of fresh culture media and an 8 cm diameter disc of **CATH‐M** or **PYRO‐M** was introduced in the Petri dish, except for the control. After an additional 8–10 h, the membranes were carefully removed, and the medium was discarded. Cells were washed once with PBS and lysed using a lysis buffer (1% NP‐40, 0.1% Sodium deoxycholate, 150 mM NaCl, 1 mM EDTA, 50 mM Tris pH 7.5) to obtain a protein suspension. Samples were subsequently processed by the CRG/UPF Proteomics Unit (Barcelona, Spain).

Post‐analysis for protein identification and quantification were performed using MaxQuant software, version 2.6.1.0 [56, 57]. The LTQ Orbitrap was operated with default parameters, enabling label‐free quantification. The UniProt reference proteome (release 2024_02) was used, limited to reviewed entries. Statistical analysis for quantification was conducted using the Differential Enrichment Proteomics (DEP) package from R‐Bioconductor [58, 59]. A filter was applied to retain proteins identified in at least two out of three replicates for at least one condition. Data normalisation was conducted using variance stabilizing transformation (vsn) from the vsn‐R package. For missing values deemed ‘Missing Not At Random’, data imputation was performed by random draws from a Gaussian distribution centred around a minimal value. Principal component analysis (PCA) was conducted on the 500 most variable proteins between groups. Differential expression analysis was performed for three comparisons: “**CATH‐M** vs control”, “**PYRO‐M** vs control”, and “**CATH‐M** vs **PYRO‐M**”, based on protein‐wise linear models and empirical Bayes statistics using the limma package. False Discovery Rates (FDRs) were estimated using the “fdrtool” R package. A Gene set enrichment analysis was conducted using the g:GOSt platform, with the g:SCS algorithm as the default method for computing multiple testing correction for p‐values gained from GO and pathway enrichment analysis. Only proteins with a p‐value lower than 0.05 from the “**CATH‐M** vs **PYRO‐M**” comparison, previously filtered for the commons expressed in both control comparisons (“**CATH‐M** vs control”, “**PYRO‐M** vs control”), were included in the enrichment.

### 4.24 Statistical Analysis

The SP‐SDS logarithmic reduction test analysis was conducted as described in previous studies.^20^ Cell viability or death was expressed as the mean ± standard deviation for each condition (percentage), with a minimum of N = 5, unless otherwise specified. Cell counting was performed using Fiji software. For crystal violet measurements, each repetition was divided into 6 replicates and read. The obtained signal was adjusted by subtracting the mean blank value and expressed relative to the “Treatment Control” as the mean (of the repetitions) ± standard deviation.

Cell migration was expressed as the mean ± standard deviation from 3 independent experiments, each containing 3 replicates, relative to the control. Adhesion strength was indicated as the mean ± standard deviation of detached cells from 4 replicates, expressed as fold change relative to the control.

OCR and ECAR signals were presented as the mean ± standard deviation of 3 replicates for each condition, with the blank automatically subtracted. In the ROS generation tests, the obtained signal was represented as the mean ± standard deviation of at least 3 repetitions for each condition.

For specific ROS species, the results from each of the 3 kits were analyzed separately. The mean of the blank was subtracted from the mean of the 5 repetitions for each condition, and the data were expressed as fold change relative to the control.

Cells were counted using ImageJ 1.54f software, and data were represented using OriginPro software.

## Conflicts of Interest

The authors declare the following competing interests: J.B.‐C., J.B., D.R.‐M., S.S.‐G., and V.J.Y. are inventors on a patent application related to the pallet technology described in this work.

## CRediT Authorship Contribution Statement

José Bolaños‐Cardet: Data curation, Formal analysis, Investigation, Visualization, Validation, Writing – original draft. Sara Pugliese: Data curation, Writing – review and editing. Jordi Bruna: Visualization, Validation, Writing – review and editing. Daniel Ruiz‐Molina: Funding acquisition, Resources, Writing – review and editing. Salvio Suárez‐García: Conceptualization, Methodology, Supervision, Validation, Visualization, Writing – original draft. Victor J. Yuste: Conceptualization, Methodology, Supervision, Validation, Visualization, Resources, Funding acquisition.

## Supporting information




**Supporting File 1**: advs73697‐sup‐0001‐SuppMat.docx.

**Supporting File**: advs73697‐sup‐0002_VideoS1.mp4.

**Supporting File**: advs73697‐sup‐0003_VideoS2.mp4.

**Supporting File**: advs73697‐sup‐0004_VideoS3.mp4.

**Supporting File**: advs73697‐sup‐0005_VideoS4.mp4.

**Supporting File**: advs73697‐sup‐0006_VideoS5.mp4.

## Data Availability

The data that support the findings of this study are available from the corresponding author upon reasonable request.
