# Supplementary material for: A Mussel‐Inspired Bioadhesive Patch to Selectively Kill Glioblastoma Cells
Source: Adv Sci (Weinh). 2026 Jan 27;13(22):e10658. doi: 10.1002/advs.202510658 (PMC13088311; doi:10.1002/advs.202510658)
Supplement: Supplementary file 1 — Supporting File 1: advs73697‐sup‐0001‐SuppMat.docx. [file ADVS-13-e10658-s001.docx]

Supporting Information

A Mussel-Inspired Bioadhesive Patch to Selectively Kill Glioblastoma Cells

*Jose Bolaños-Cardet, Sara Pugliese, Jordi Bruna, Daniel Ruiz-Molina,* Salvio Suárez-García,* Victor J. Yuste**

*Corresponding authors: [dani.ruiz@molina](mailto:dani.ruiz@molina); [salvio.suarez@icn2.cat](mailto:salvio.suarez@icn2.cat); [victor.yuste@uab.cat](mailto:victor.yuste@uab.cat)

**Table of Contents**

[S1. CATH-M and PYRO-M membranes synthesis and manipulation 3](#_Toc205144927)

[S2. FTIR analysis of the synthesized membranes 4](#_Toc205144928)

[S3. X-ray photoelectron spectroscopy curve-fitting of the synthesized membranes. 5](#_Toc205144929)

[Table S1. Elemental analysis. 6](#_Toc205144930)

[S4. Morphology of the membranes. 7](#_Toc205144931)

[S5. Long-term degradation test. 8](#_Toc205144932)

[S6. Morphological changes after degradation test. 9](#_Toc205144933)

[S7. Chemical changes after degradation test. 10](#_Toc205144934)

[S8. Antibacterial properties. 11](#_Toc205144935)

[S9. CATH-M membrane manipulability in a real environment. 12](#_Toc205144936)

[S10. *Ex vivo* adhesion on brain. 13](#_Toc205144937)

[S11. Mechanical and adhesion tests. 14](#_Toc205144938)

[S12. Glioblastoma cells cytotoxic screening. 15](#_Toc205144939)

[S13. Effect against different tumor cells lines and healthy cells. 16](#_Toc205144940)

[S14. CATH-H time course cytotoxicity. 17](#_Toc205144941)

[S15. Clonogenic test. 18](#_Toc205144942)

[S16. Local effect. 19](#_Toc205144943)

[S17. Comparison with standard-of-care treatments. 20](#_Toc205144944)

[Table S2. Inhibitors and inducers details. 21](#_Toc205144945)

[S18. CATH-H Caspase-independency cell death. 22](#_Toc205144946)

[S19. CATH-M and necroptosis inhibitor. 23](#_Toc205144947)

[S20. CATH-M and autophagy inhibitors. 24](#_Toc205144948)

[S21 CATH-M and parthanatos inhibitors. 25](#_Toc205144949)

[S22. CATH-M and macromolecular synthesis inhibitors. 26](#_Toc205144950)

[S23. CATH-M and ferroptosis inhibitors. 27](#_Toc205144951)

[S24. CATH-M and oxidative damage inhibitor. 28](#_Toc205144952)

[S25. LN229 morphology over the membranes. 29](#_Toc205144953)

[S26. LN229 cell death over the membranes. 30](#_Toc205144954)

[S27. Primary cortical astrocytes morphology over the membranes. 31](#_Toc205144955)

[S28. Primary cortical astrocytes viability over the membranes. 32](#_Toc205144956)

[S29. Spheroids early culture by scanning electron microscopy (SEM). 33](#_Toc205144957)

[S30. Spheroids early culture by optical microscopy. 34](#_Toc205144958)

[S31. Spheroids progression. 35](#_Toc205144959)

[S32. Inserts test scheme. 36](#_Toc205144960)

[S33. Influence in the LN229 cell migration. 37](#_Toc205144961)

[S34. Migrated and non-migrated cells visualization. 38](#_Toc205144962)

[S35. Membranes strong cell adhesion. 39](#_Toc205144963)

[S36. Alterations in the LN229 internal structures. 40](#_Toc205144964)

[S37. Seahorse test. 41](#_Toc205144965)

[S38. LN229 cell death under oxidative stress. 42](#_Toc205144966)

[S39. CATH-M reactive oxygen species (ROS) production properties. 43](#_Toc205144967)

[S40. Lipid peroxidation. 44](#_Toc205144968)

[S41. LN229-lysate protein microarray. 45](#_Toc205144969)

**Video S1.** Synthesis of the **CATH-M** and **PYRO-M** membranes.

**Video S2**. Manipulation of the **CATH-M** and **PYRO-M** membranes.

**Video S3.** **CATH-M** insertion in a brain cavity. Application and removal of an 8 mm **CATH-M** disc in an *ex vivo* model (pig brain), demonstrating the manipulability and flexibility.

**Video S4.** Representative bright field and Hoechst 33342 (H42) composition of the LN229 cells that remained in the **CATH-M** and **PYRO-M** membranes after being trypsinized. The cells are homogeneously distributed along the shape of the membranes.

**Video S5.** Lipid peroxidation. 3D visualization of the lipid peroxidation results for the control, **CATH-M** and **PYRO-M**. Blue = nuclei, RED = dye reduced-state, Green= dye oxidised-state.

# **S1. CATH-M and PYRO-M membranes synthesis and manipulation**


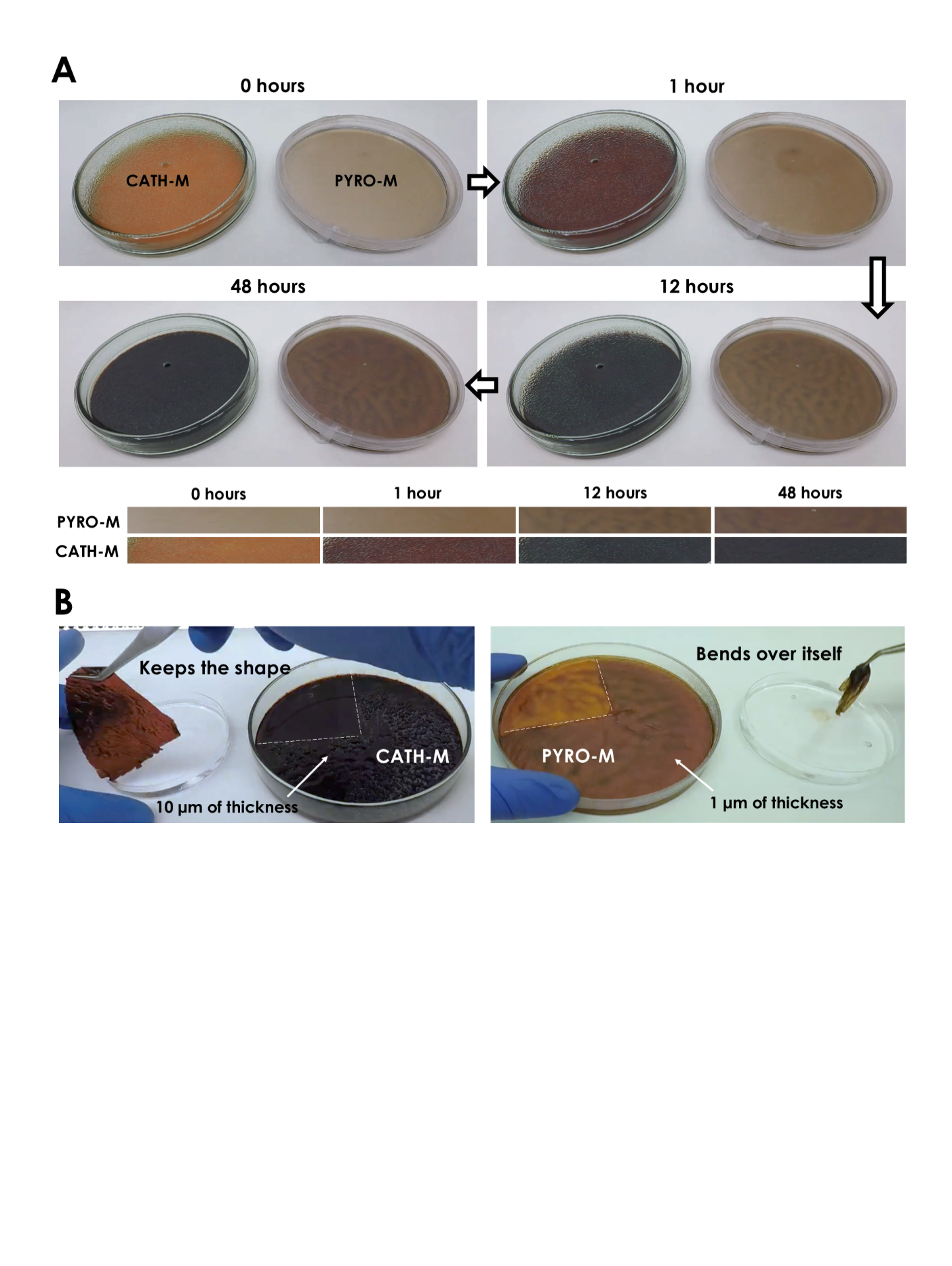


**Figure S1. CATH-M and PYRO-M membranes synthesis and manipulation. A)** 48-hours time lapse of the synthesis, where a constant darkening of the solutions can be observed due the oxidation in base of the time, leading to the formation of both membranes. **B)** Pieces **CATH-M** and **PYRO-M** when removed from a liquid environment. While **CATH-M** retains its shape outside the medium, remaining flat, **PYRO-M** collapses onto itself.

# **S2. FTIR analysis of the synthesized membranes**


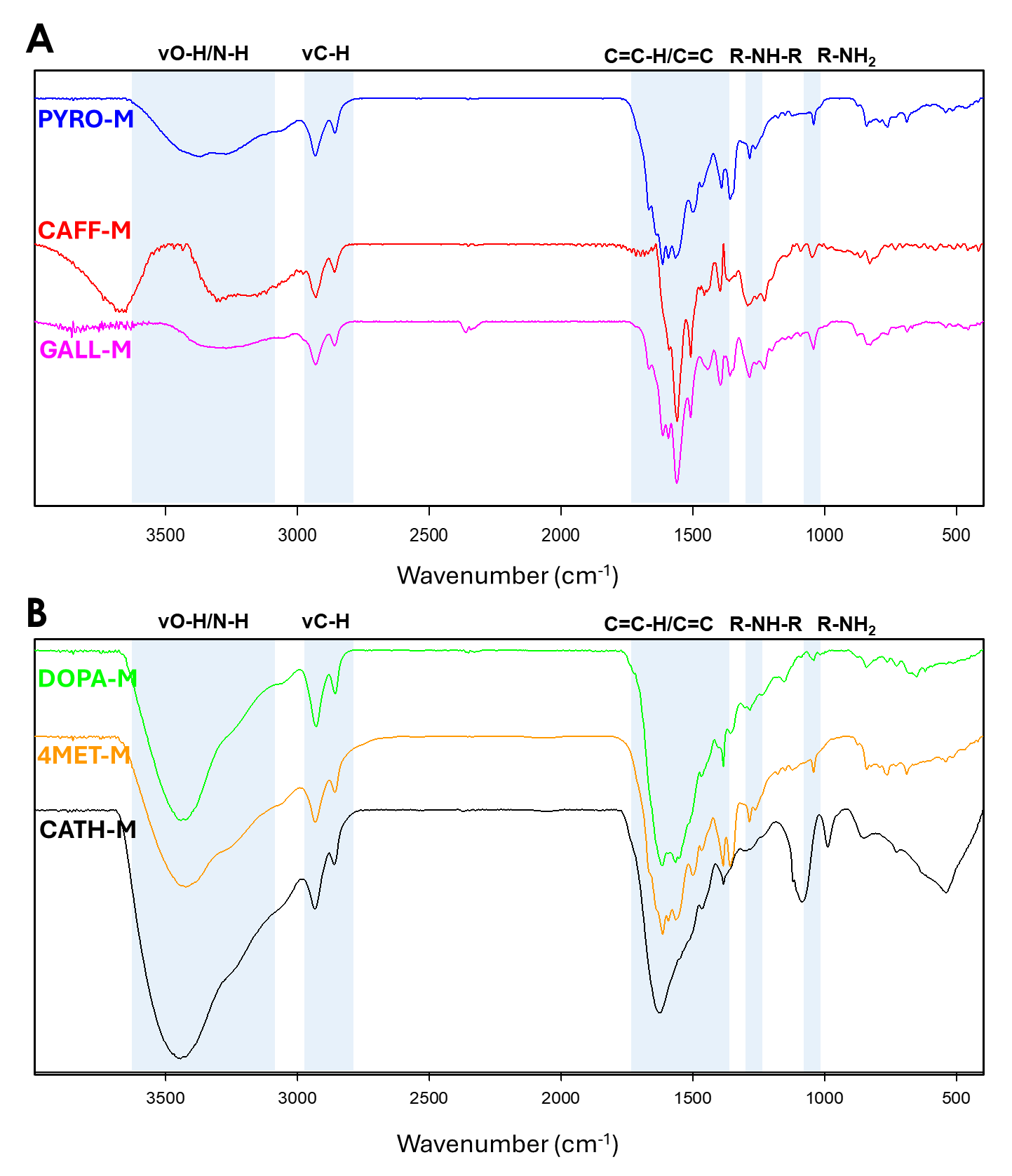


**Figure S2. FTIR analysis of the synthesised membranes.** FTIR spectra of **A)** **PYRO-M**, **CAFF-M** and **GALL-M** and **B)** **DOPA-M**, **4MET-M** and **CATH-M**.

# **S3. X-ray photoelectron spectroscopy curve-fitting of the synthesized membranes.**

**
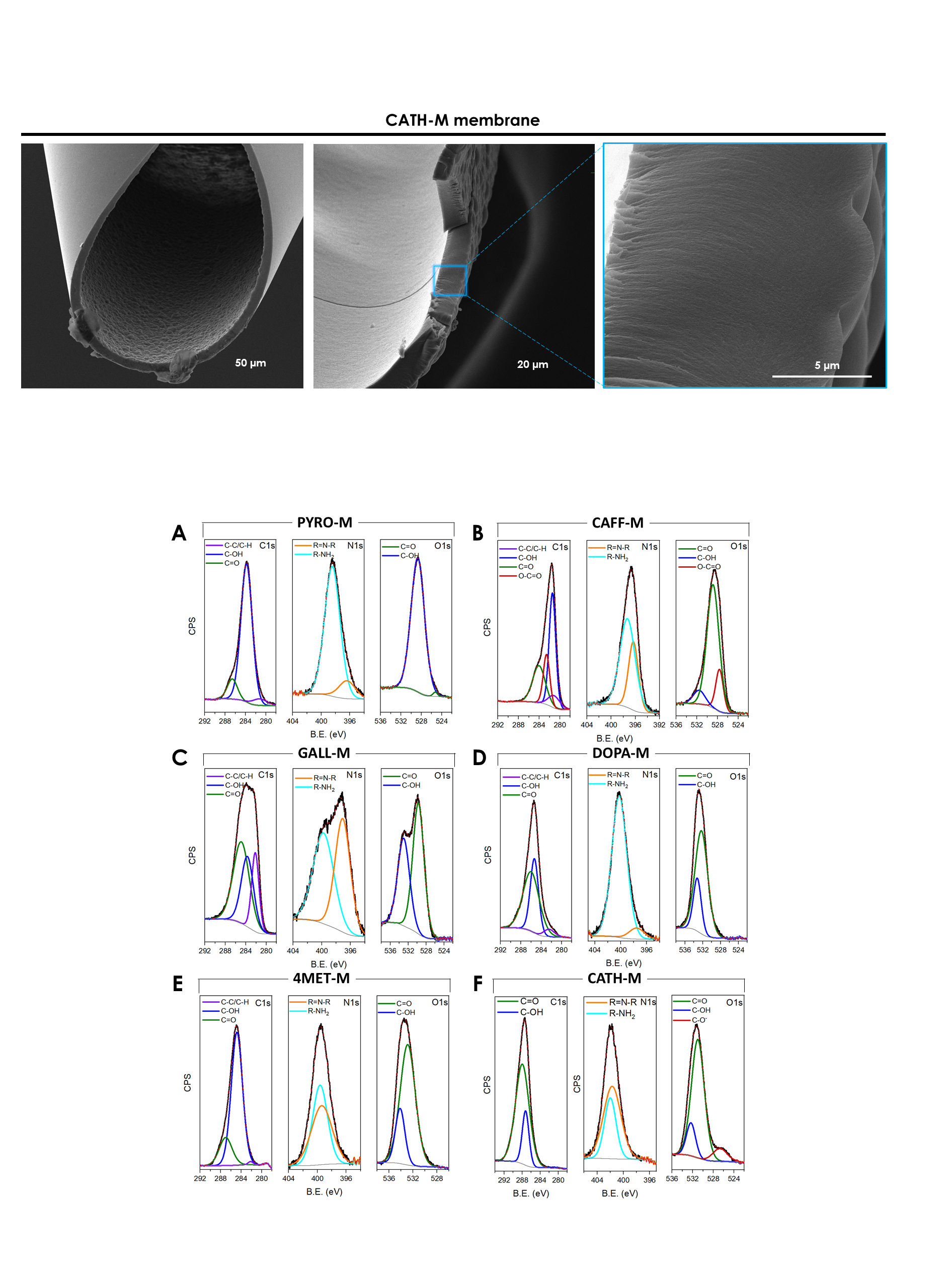
**

**Figure S3. X-ray photoelectron spectroscopy curve-fitting of the synthesised membranes.** Curve-fitting results for C1s, N1s and O1s high-resolution XPS spectra for **A) PYRO-M**, **B) CAFF-M**, **C) GALL-M**, **D) DOPA-M**, **E) 4MET-M** and **F) CATH-M**. CPS: Counts per second.

# **Table S1.** [**Elemental analysis.**](#_Toc179016516)

**Table S1. Elemental analysis.** Catechol:Amine ratio during the preparation of the membrane and the observed in the resulting polymer (with their corresponding empiric formulas).

| **Membrane** | **Catechol : Amine theoretical ratio** | **Catechol : Amine experimental ratio** | **Empiric formula** | | | |
| --- | --- | --- | --- | --- | --- | --- |
|  |  |  | **C** | **H** | **O** | **N** |
| PYRO-M | 1 : 1.5 | 1 : 0.81 ± 0.03 | 6.09 ± 0.17 | 9.26 ± 0.28 | 1.23 ± 0.04 | 1 ± 0 .03 |
| DOPA-M | 1 : 2.5 | 1 : 0.42 ± 0.01 | 5.38 ± 0.15 | 7.7 ± 0.04 | 1.1 ± 0.02 | 1 ± 0.02 |
| CAFF-M | 1 : 2 | 1 : 1.24 ± 0.02 | 7 ± 0.05 | 10.26 ± 0.14 | 1.62 ± 0.02 | 1 ± 0.01 |
| GALL-M | 1 : 2 | 1 : 0.87 ± 0.02 | 6.37 ± 0.11 | 9.77 ± 0.06 | 1.73 ± 0.05 | 1 ± 0.01 |
| 4MET-M | 1 : 1.5 | 1 : 0.62 ± 0.13 | 8.72 ± 0.07 | 12.21 ± 0.04 | 1.71 ± 0.42 | 1 ± 0.01 |
| CATH-M | 1 : 2.5 | 1 : 0.87 ± 0.03 | 9.71 ± 0.18 | 15.07 ± 0.17 | 3.45 ± 0.12 | 1 ± 0.02 |

# **S4. Morphology of the membranes.**


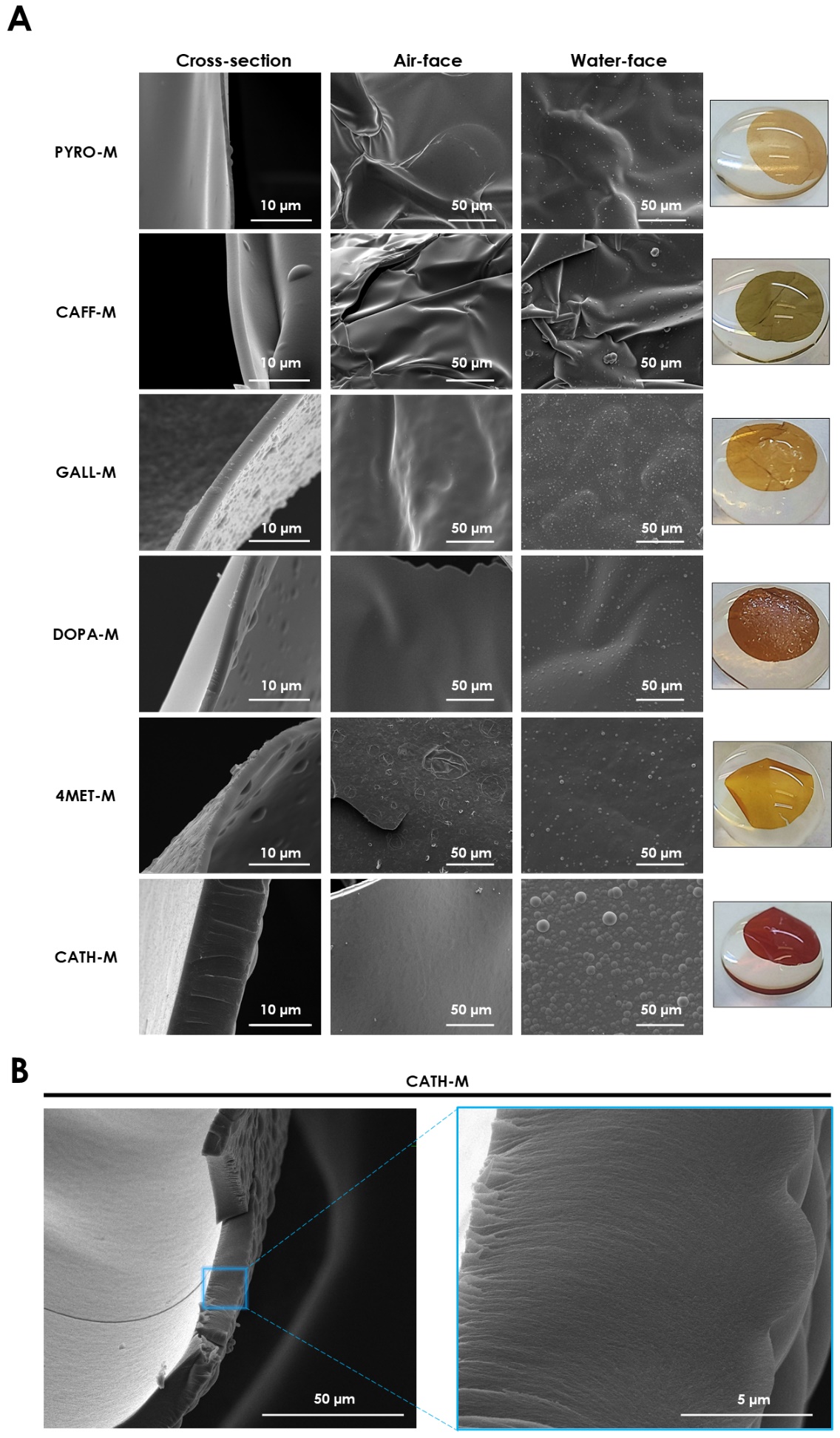


**Figure S4. Morphology of the membranes**. **A)** Scanning electron microscopy (SEM) images comparing the cross-section, air-face, water-face, and visual aspect inside water of the six different membranes. **B)** Close view of the **CATH-M** cross-section, where no porous structures are observed.

# **S5. Long-term degradation test.**


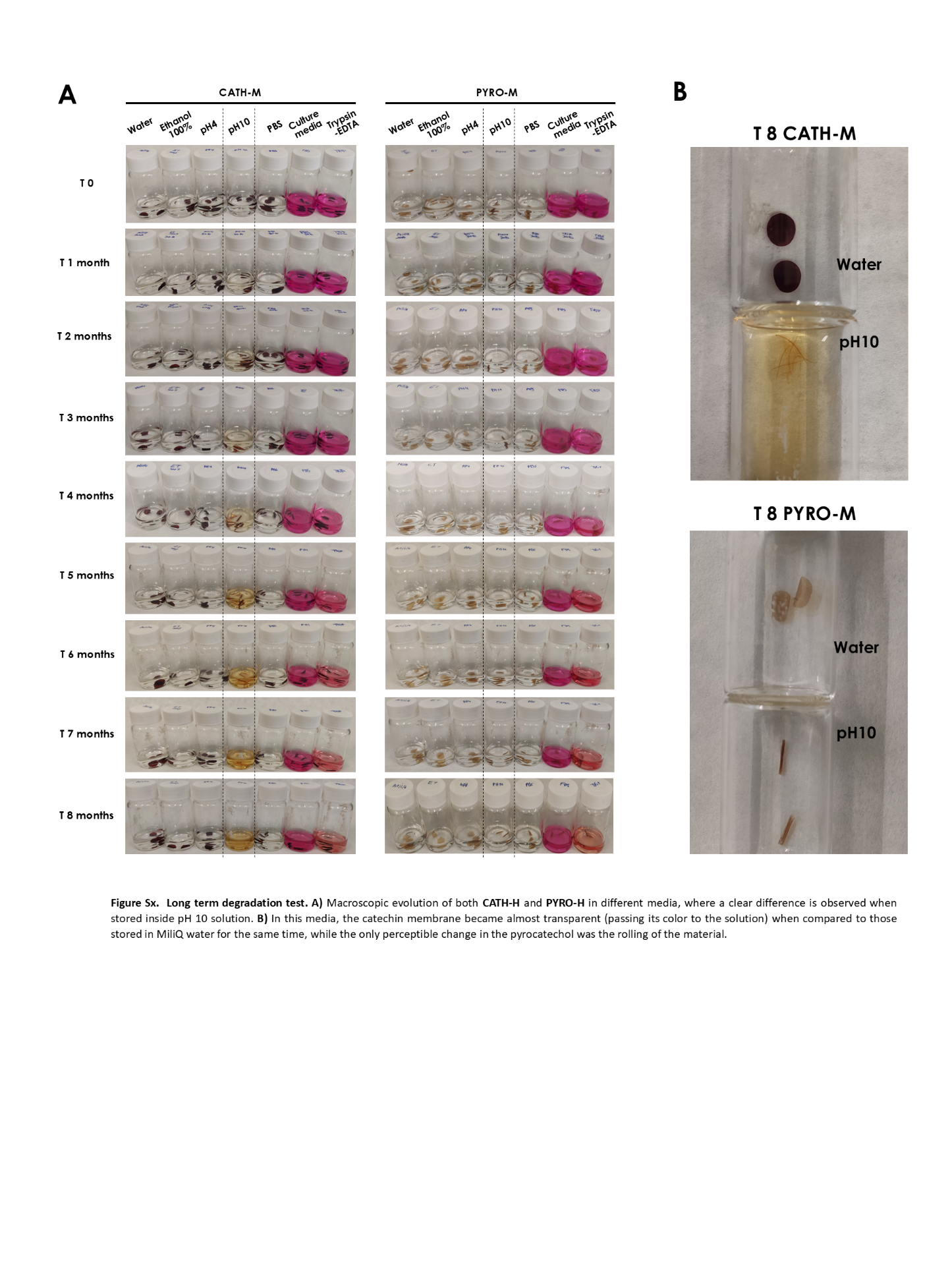


**Figure S5. Long-term degradation test. A)** Macroscopic evolution of both **CATH-M** and **PYRO-M** in different media, where a clear difference is observed when stored inside pH 10 solution. **B)** In this media, the catechin membrane became almost transparent (passing its colour to the solution) when compared to those stored in water for the same time, whereas the only perceptible change in the pyrocatechol was the rolling of the material.

# **S6. Morphological changes after degradation test.**


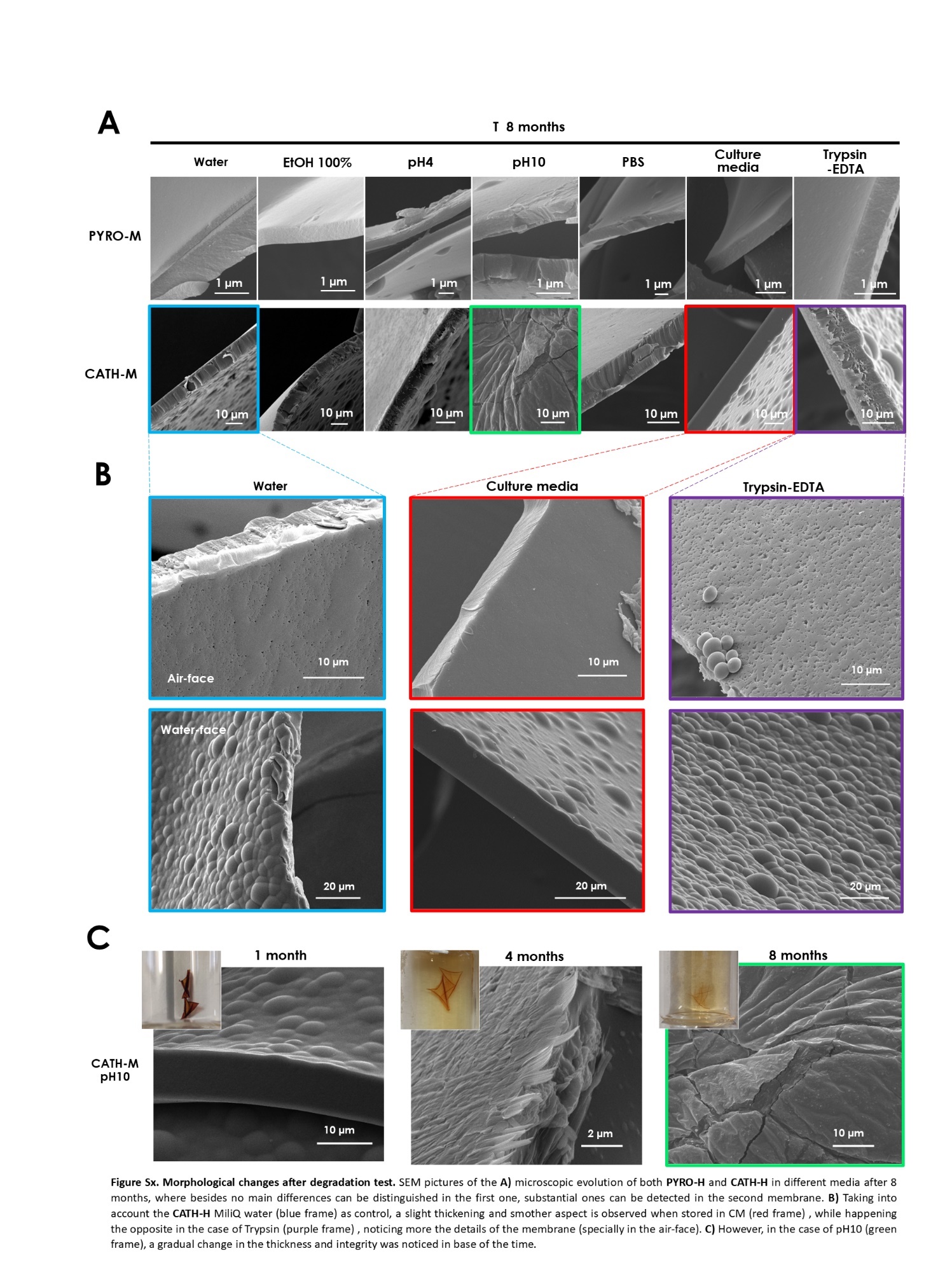


**Figure S6. Morphological changes after degradation test.** SEM images of the **A)** microscopic evolution of both **PYRO-M** and **CATH-M** in different media after eight months. No big differences can be distinguished in the first one, whereas substantial changes can be detected in the second membrane. **B)** Taking into account the **CATH-M** in water (blue frame) as a control, a slight thickening and smother appearance is observed when stored in culture media (red frame). Conversely, in the case of Trypsin-EDTA (purple frame), more details of the membrane (especially in the air-facing side) can be observed. **C)** In the case of pH 10 (green frame), a gradual change in the thickness and integrity was noticed with the time.

# **S7. Chemical changes after degradation test.**


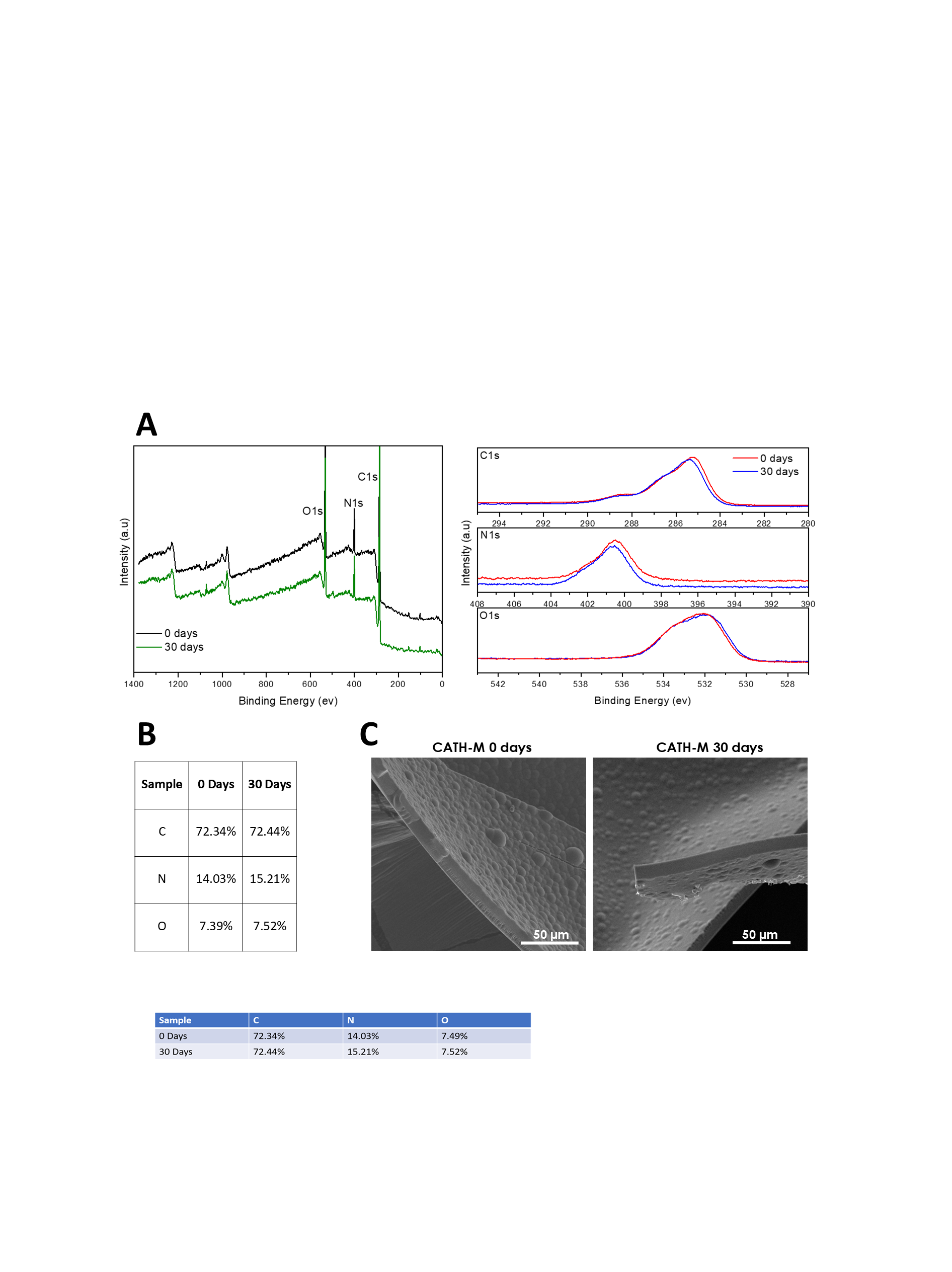


**Figure S7. Chemical stability and morphology over time.** A **CATH-M** was specifically analysed at two distinct time points (initial synthesis (Day 0) and after 30 days of storage in humid conditions) by different techniques: **A)** Variations in its XPS spectra. **B)** Elemental analysis. **C)** SEM images.

# **S8. Antibacterial properties.**


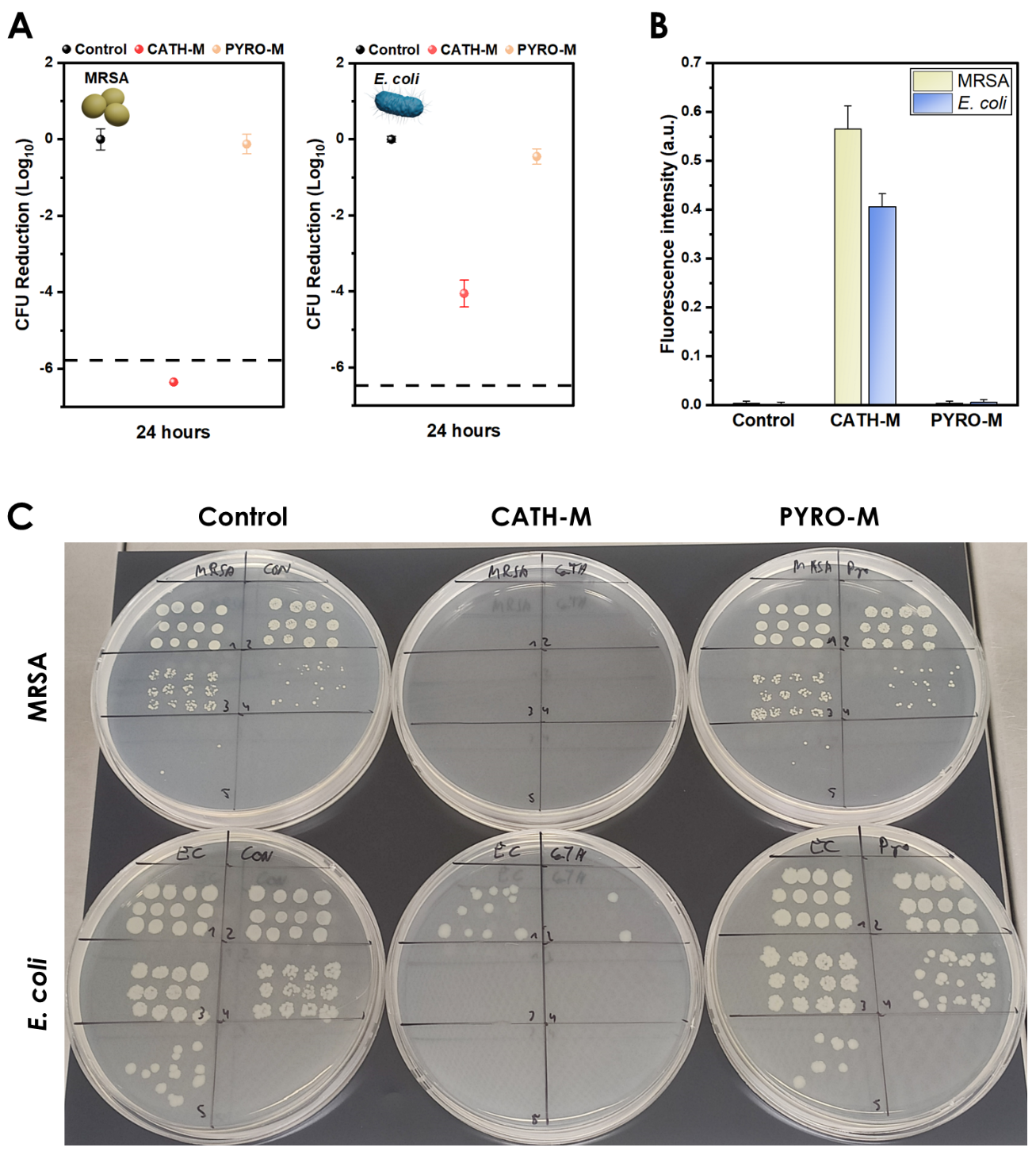


**Figure S8. Antibacterial properties. A)** Colony forming units (CFU) logarithmic reduction of MRSA and *E. coli* after being exposed to **CATH-M** or to a **PYRO-M** for 24 h and **B)** the reactive oxygen species (ROS) generated in the bacteria suspension in which the membranes were introduced after also 24 h. **C)** Representative single plate-serial dilution spotting (SP-SDS) plates for both bacteria and membranes. Error bars represent the standard deviation of a data set relative to the mean.

# **S9. CATH-M membrane manipulability in a real environment.**


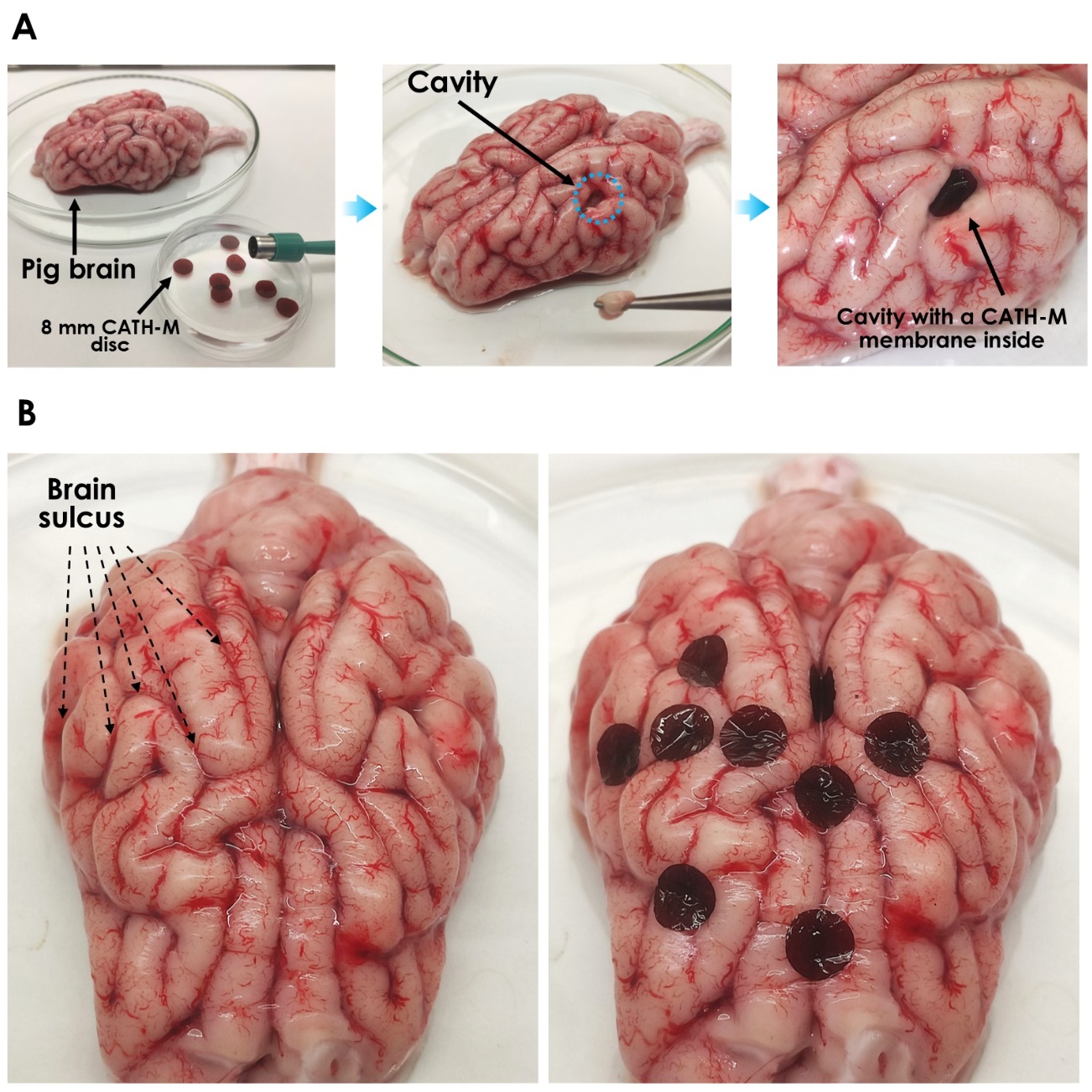


**Figure S9. CATH-M membrane manipulability in a real environment.** **A)** Representation of a **CATH-M** application in a glioblastoma-resection cavity using an *ex vivo* model (pig brain). **B)** Comparison of a pig brain without (left) and with (right) several membrane discs adhered to distinct parts of its surface, being able to bend and adapt to the different shapes of the brain, especially in the sulcus.

# **S10. *Ex vivo* adhesion on brain.**


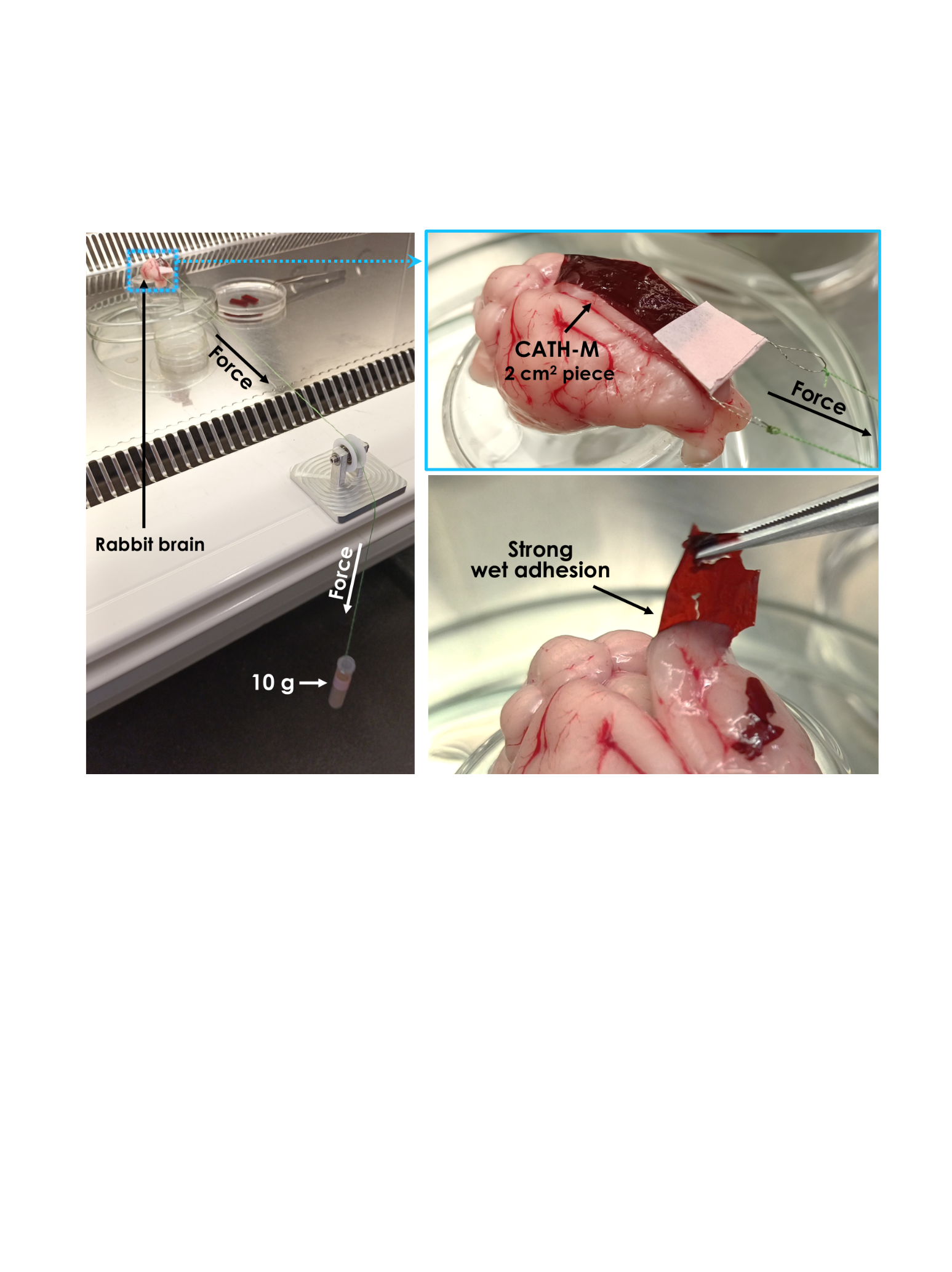


**Figure S10. CATH-M *ex vivo* adhesion.** Setup established to test the shear force that a 2 cm^2^ piece of **CATH-M** can withstand when adhered to a rabbit brain, demonstrating resistance up to 10 g. It can also be observed how the membrane gently pulls the tissue when attempts are made to remove it.

# **S11. Mechanical and adhesion tests.**


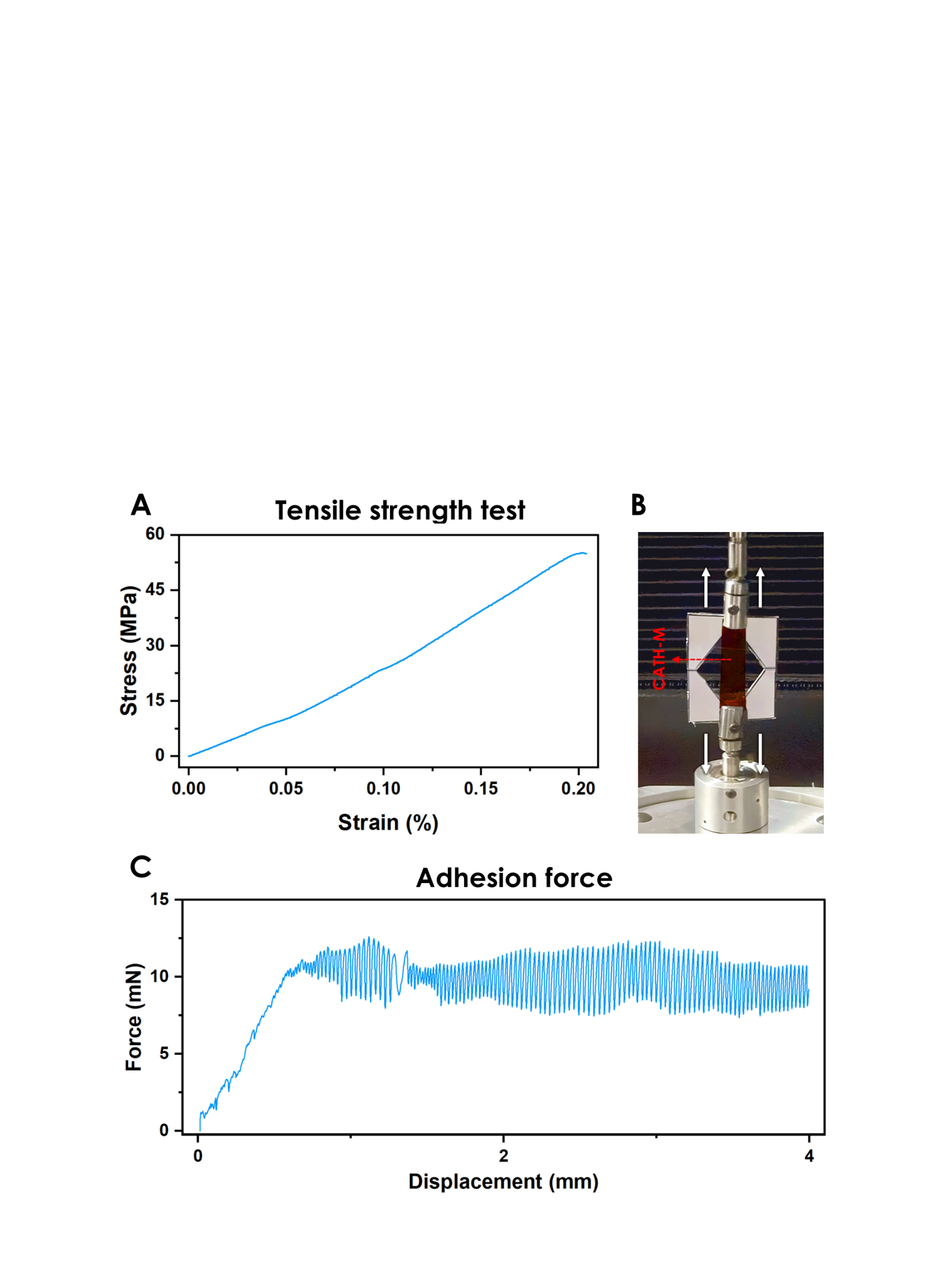


**Figure S11. Quantitative mechanical and adhesion properties of CTAH-M.** **A)** Tensile strength characterization of a **CATH-M** membrane. The stress–strain curve reveals material failure at a stress of 57 MPa and a strain of 0.2%, following a transition from elastic to plastic deformation. **B)** Setup of the uniaxial tensile test. **C)** Adhesion force profile obtained from a uniaxial shear test between the catechin membrane and a biological substrate. A peak adhesion force of 12.6 mN was observed at 0.7 mm displacement, followed by a steady-state sliding regime around 10 mN, indicating stable interfacial bonding and ductile-like sliding behaviour under shear loading.

# **S12. Glioblastoma cells cytotoxic screening.**


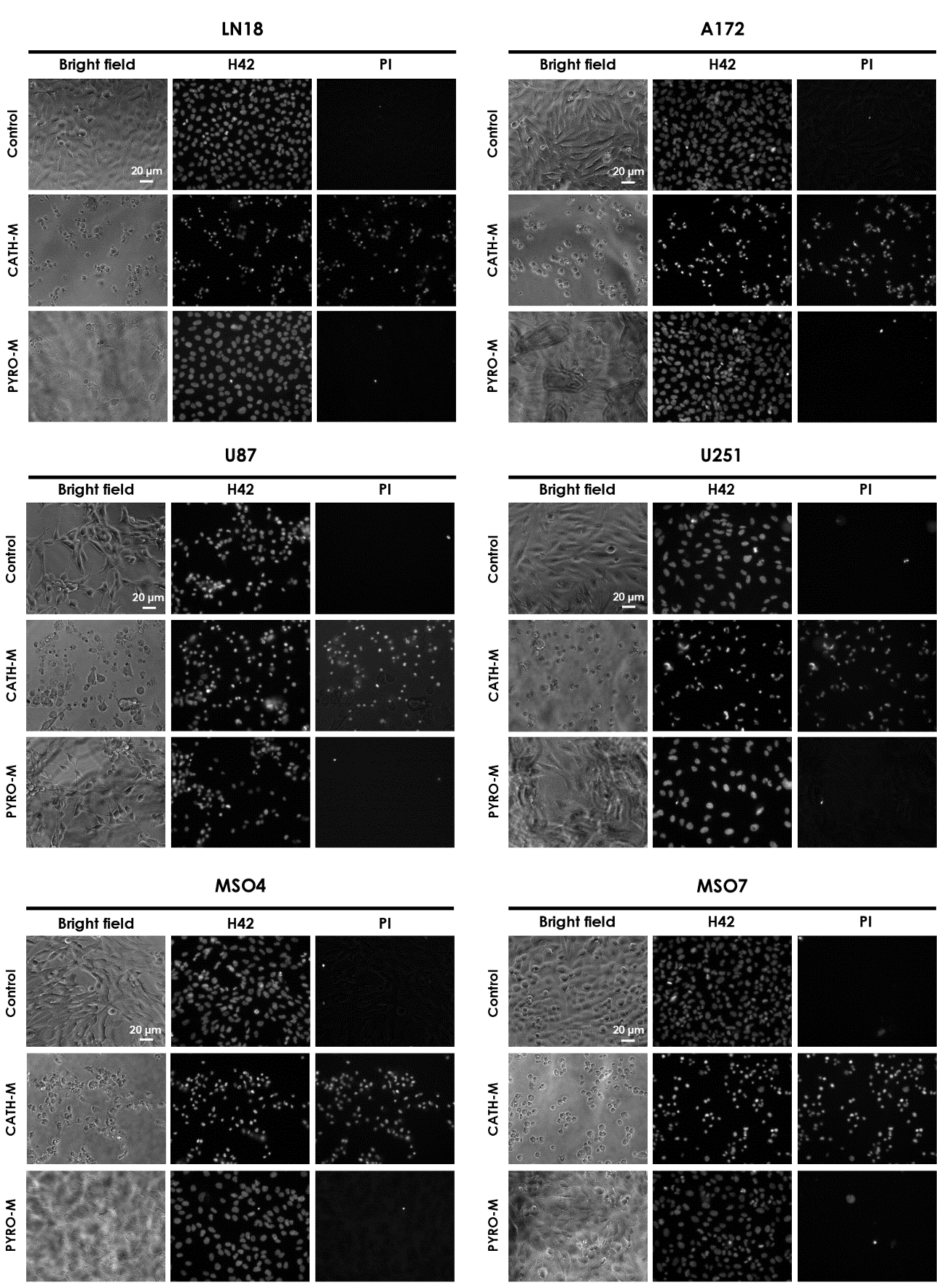


**Figure S12. Glioblastoma cells cytotoxic screening**. Representative bright field, Hoechst 33342 (H42) and propidium iodide (PI) images of the four additional glioblastoma cell lines (LN18, A172, U87 and U251) and the two glioblastoma primary cultures (MSO4 and MSO7) after being treated with the **CATH-M** and **PYRO-M** membranes, where a clear difference in the PI of **CATH-M** can be observed in all the cases.

# **S13. Effect against different tumor cells lines and healthy cells.**


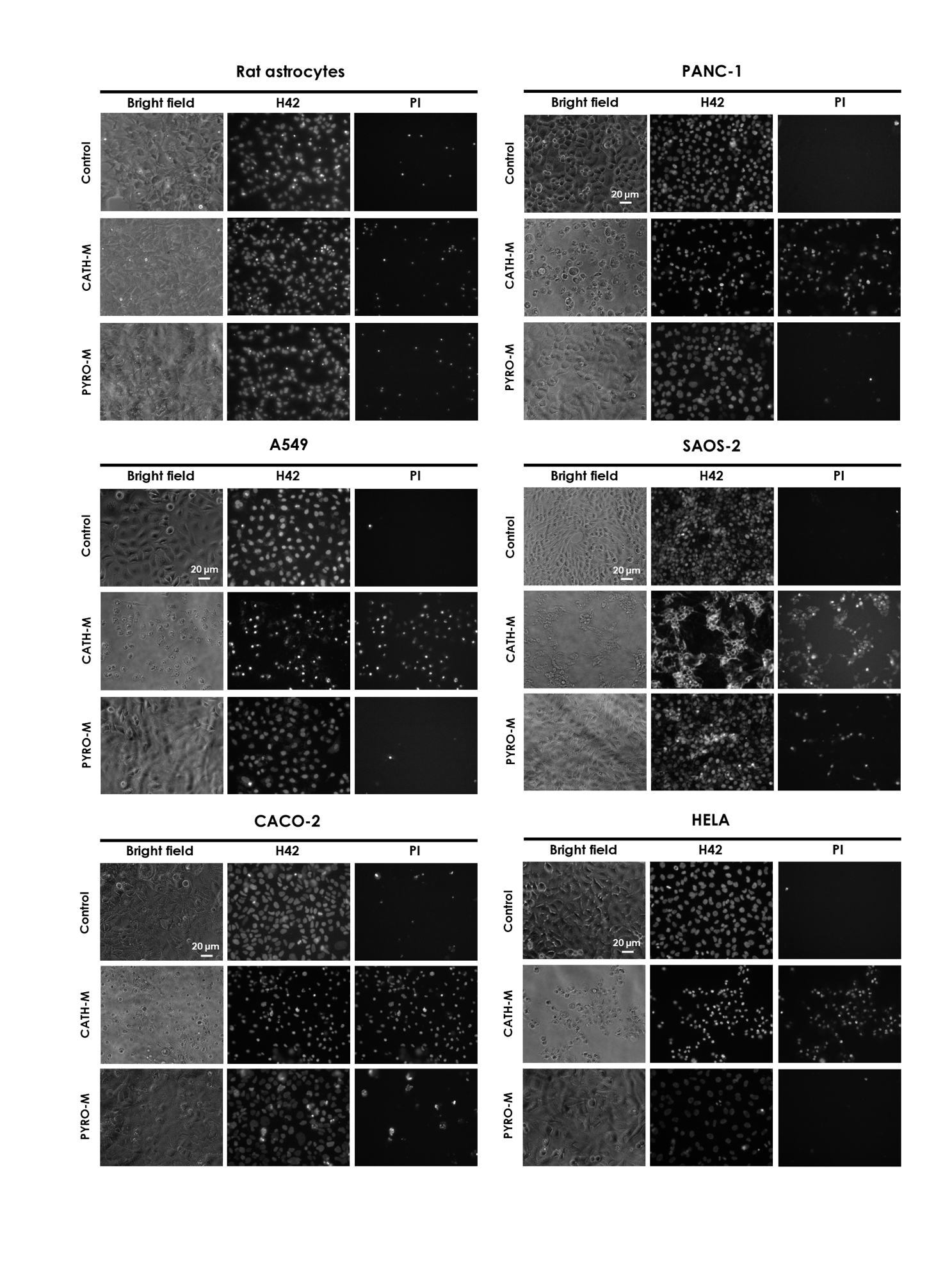


**Figure S13. Effect against astrocytes and different tumor cells lines**. Representative bright field, Hoechst 33342 (H42) and propidium iodide (PI) images of rat astrocytes (primary culture) and five tumor cell lines from different organs (A549, PANC-1, CACO-2, SAOS-2 and HELA) after being treated with the **CATH-M** and **PYRO-M** membranes, where a clear difference in the PI of **CATH-M** can be observed in all the cases of cancer cell lines.

# **S14. CATH-H time course cytotoxicity.**


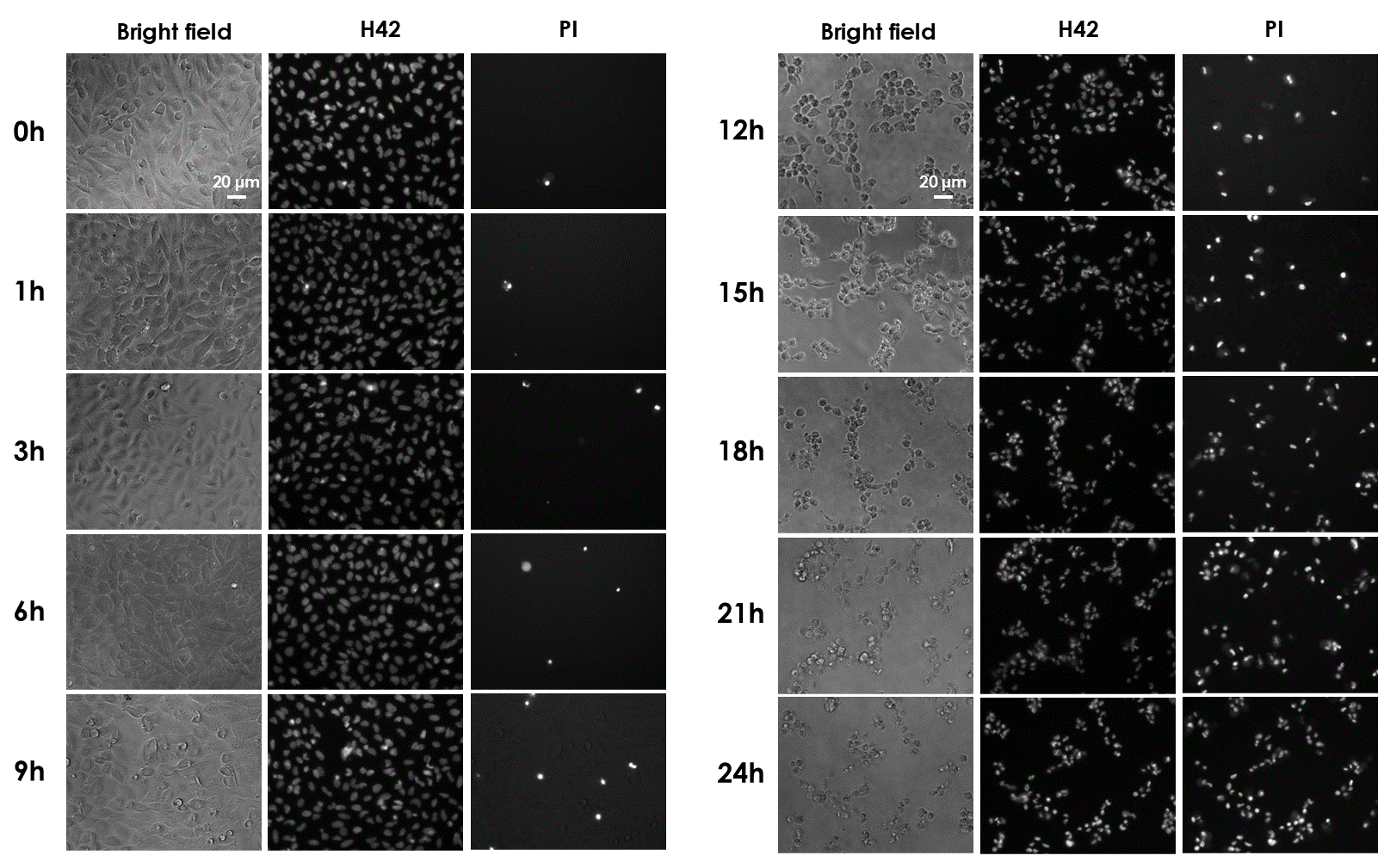


**Figure S14. CATH-H time course cytotoxicity**. Representative bright field, H42 and PI images of the LN229 cells after gradually raising the exposure time to the **CATH-M** from 0 to 24 h, where an increasing signal was observed in the PI in base of the time.

# **S15. Clonogenic test.**


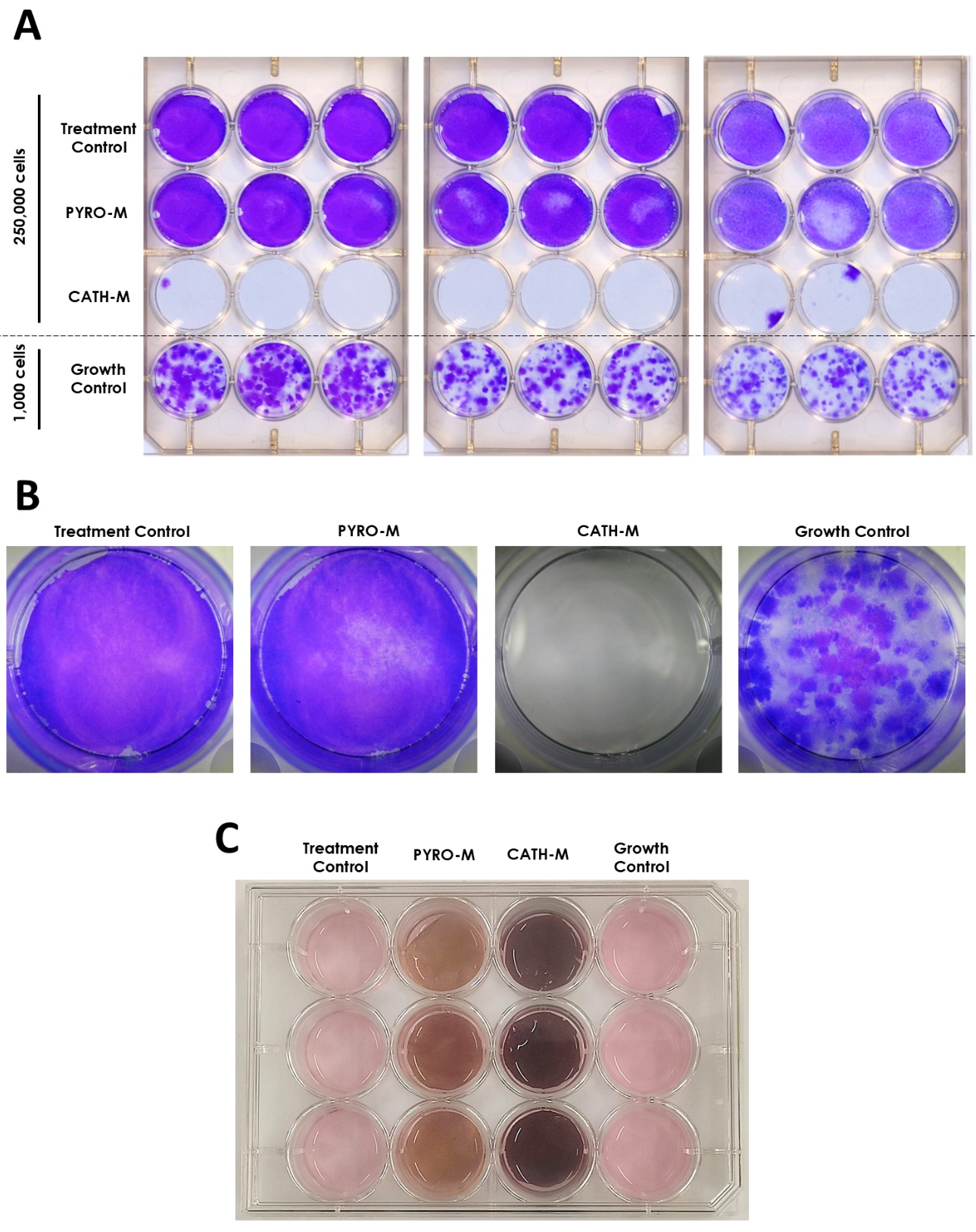


**Figure S15. Clonogenic test. A)** 3 of the four plates used in the clonogenic test, where the homogeneity of the results can be confirmed. **B)** Representative close images of a repetition from each condition, highlighting that in 9 of 12, **CATH-M** was completely empty. **C**) Visual example of the test setup, in which a disc with a similar dimension of the well’s diameter was cut and used as treatment.

# **S16. Local effect.**


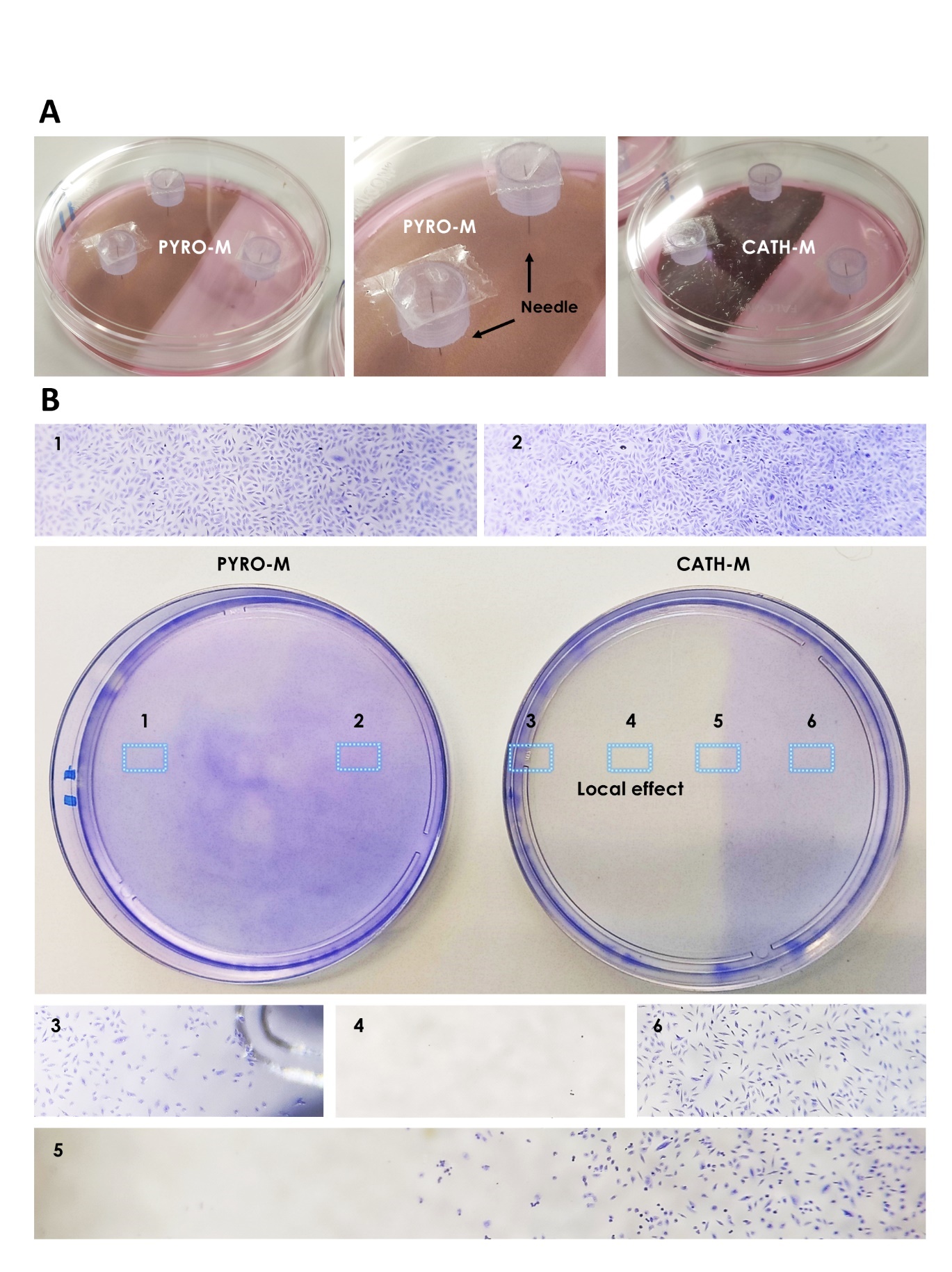


**Figure S16. CATH-M local effect. A)** LN229 cells were exposed to **PYRO-M** and **CATH-M** membranes placed on only half of each culture plate, fixed in position using needles. **B)** After 24 h, crystal violet staining revealed that **CATH-M** exerted a localized cytotoxic effect, eliminating the cells directly under the membrane, while the other half of the plate remained viable. In contrast, **PYRO-M** did not exhibit any cytotoxic effect either in close contact (under) or adjacent to the membrane. These results confirm the localized cytotoxicity of the **CATH-M** membrane.

# **S17. Comparison with standard-of-care treatments.**


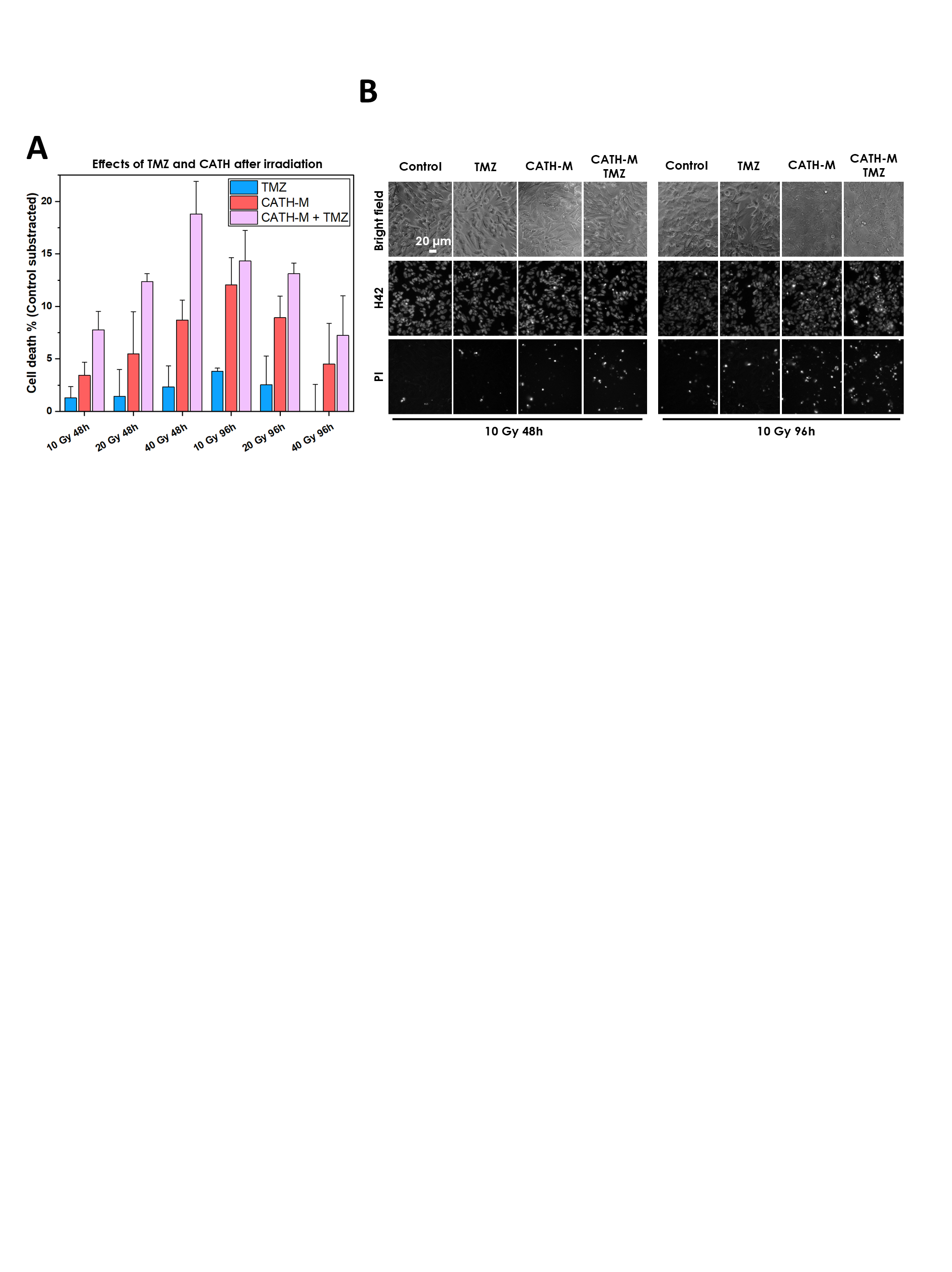


**Figure S17. CATH-M improves glioblastoma standard-of-care treatments *in vitro*.** Representative bright field, H42 and PI images of the LN229 exposed to the lowest Gy and in presence or absence of temozolomide (TMZ) and the **CATH-M** membrane for 48h and 96h.

# **Table S2. Inhibitors and inducers details.**

**Table S2. Inhibitors and inducers details**

| **Section** | **Name** | **Abbreviation** | **Concentration** | **Function** |
| --- | --- | --- | --- | --- |
| **Apoptosis** | Q-VD-OPh | QVD | 50 µM | Broad-spectrum caspase inhibitor, prevents apoptosis |
|  | Staurosporine | STAURO | 1 µM | Potent kinase inhibitor, induces apoptosis via caspase activation |
| **Necroptosis** | Necrostatin-1 | NEC1 | 50 µM | Inhibits necroptosis by blocking RIPK1 kinase activity |
| **Autophagy** | 3-Methyladenine | 3MA | 10 mM | Inhibits autophagy by blocking PI3K activity |
|  | Bafilomycin | BAFI | 100 nM | Blocks autophagy by inhibiting V-ATPase in lysosomes |
|  | Hydroxychloroquine | HYCL | 20 µM | Inhibits autophagy by preventing lysosomal acidification |
| **Parthanatos** | Rucaparib | RUCA | 10 µM | PARP inhibitor, prevents DNA repair in cancer cells |
|  | Olaparib | OLAP | 10 µM | PARP inhibitor, induces synthetic lethality in BRCA-mutant cells |
|  | PJ43 | PJ34 | 10 µM | PARP inhibitor, promotes apoptosis and DNA repair disruption |
| **Macromolecular synthesis** | Cycloheximide | CHX | 10 ug/ml | Inhibits protein synthesis by blocking translational elongation |
|  | Actinomycin D | ACTD | 10 nM | Inhibits RNA synthesis, triggers apoptosis |
| **Ferroptosis** | Deferoxamine | DEF | 100 µM | Iron chelator, prevents oxidative damage and ferroptosis |
|  | Ferrostatin-1 | FER1 | 10 µM | Inhibits ferroptosis, prevents iron-dependent lipid peroxidation |
|  | Erastine | ERA | 10 µM | Induces ferroptosis by inhibiting the cystine/glutamate antiporter |
| **Oxidative damage** | N-acetylcysteine | NAC | 5 mM | Antioxidant, replenishes glutathione and reduces oxidative stress |

# **S18. CATH-H Caspase-independency cell death.**


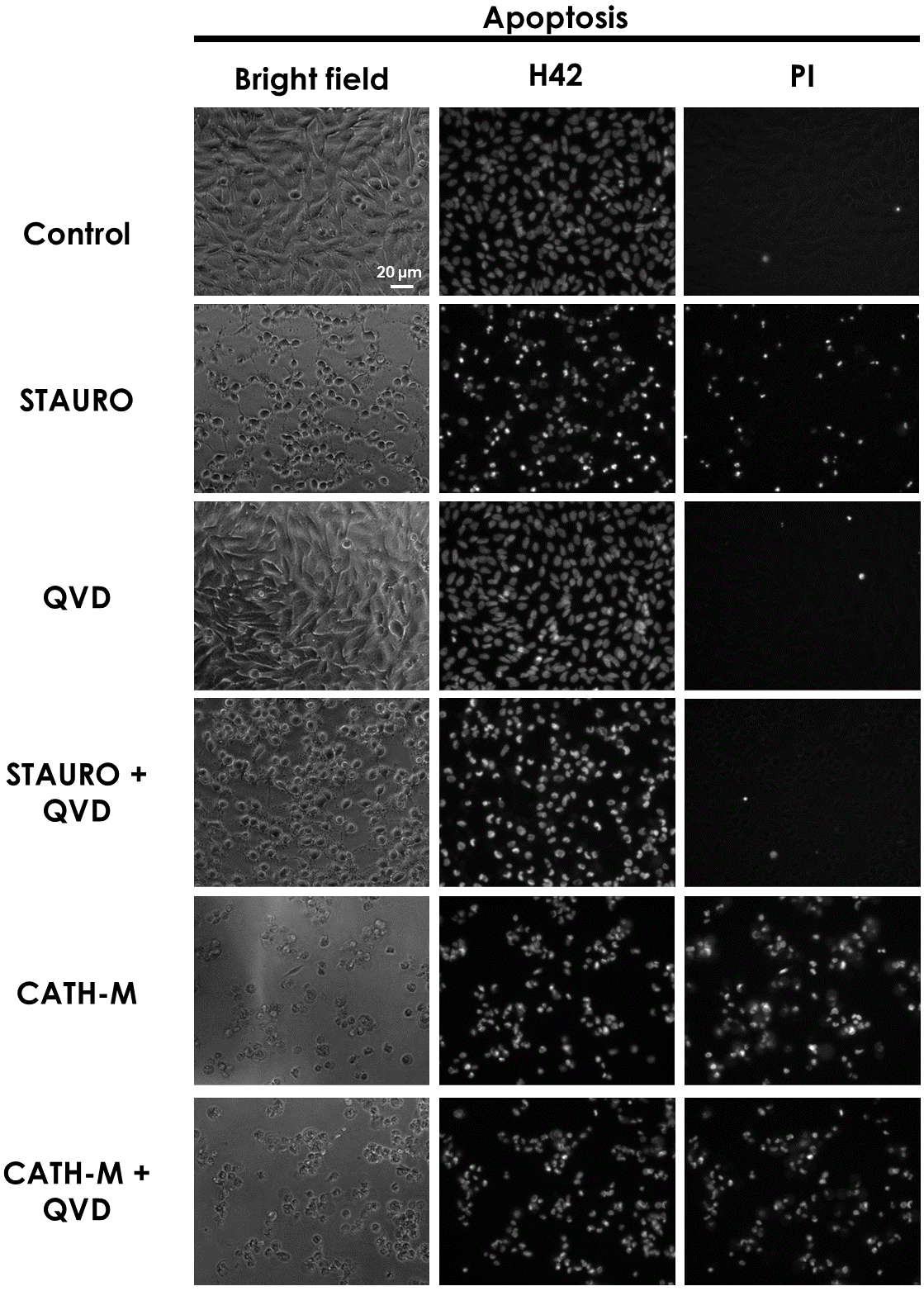


**Figure S18. CATH-H Caspase-independency cell death.** Representative bright field, H42 and PI images of the LN229 cells after using the pan-caspase inhibitor QVD simultaneously with staurosporine (apoptosis inducer), suggesting that, since QVD cannot protect the cells from the **CATH-M** cell death, this process should be caspase-independent.

# **S19. CATH-M and necroptosis inhibitor.**


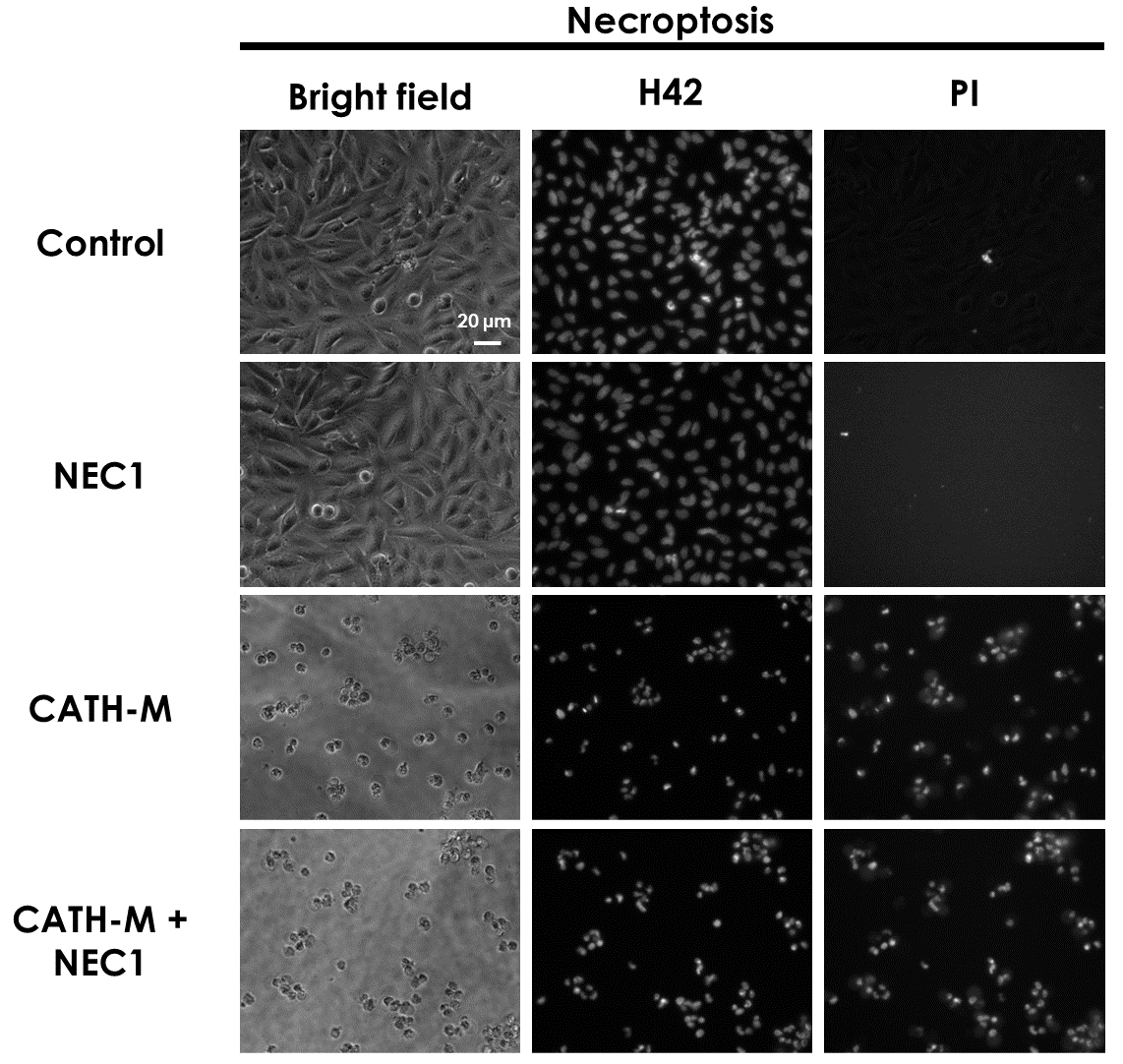


**Figure S19. CATH-M and necroptosis inhibitor.** Representative bright field, H42 and PI images of the LN229 cells after using necrostatin-1.

# **S20. CATH-M and autophagy inhibitors.**


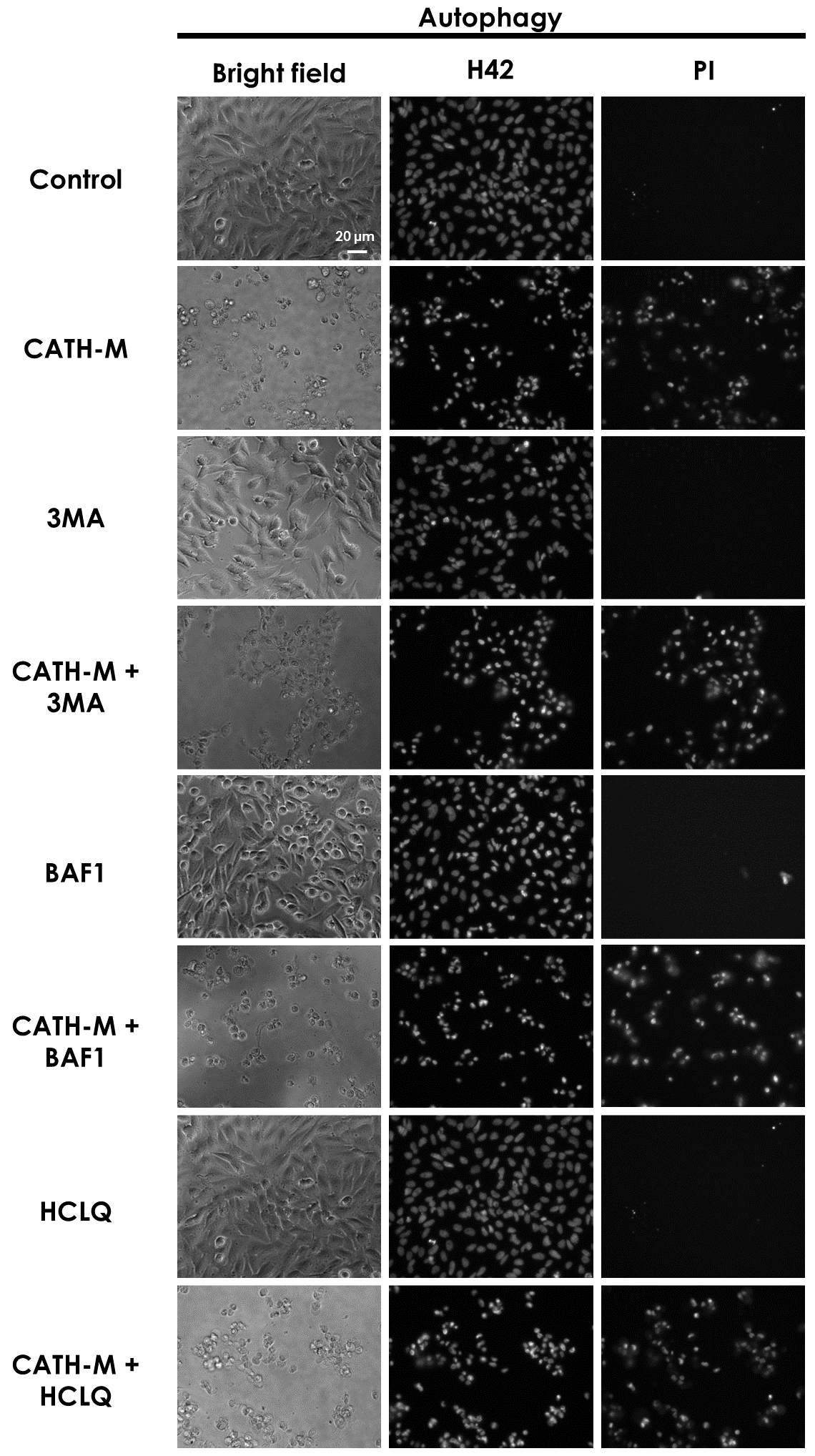


**Figure S20. CATH-M and autophagy inhibitors.** Representative bright field, H42 and PI images of the LN229 cells after using the inhibitors 3-methyladenine, bafilomycin and hydroxychloroquine.

# **S21 CATH-M and parthanatos inhibitors.**


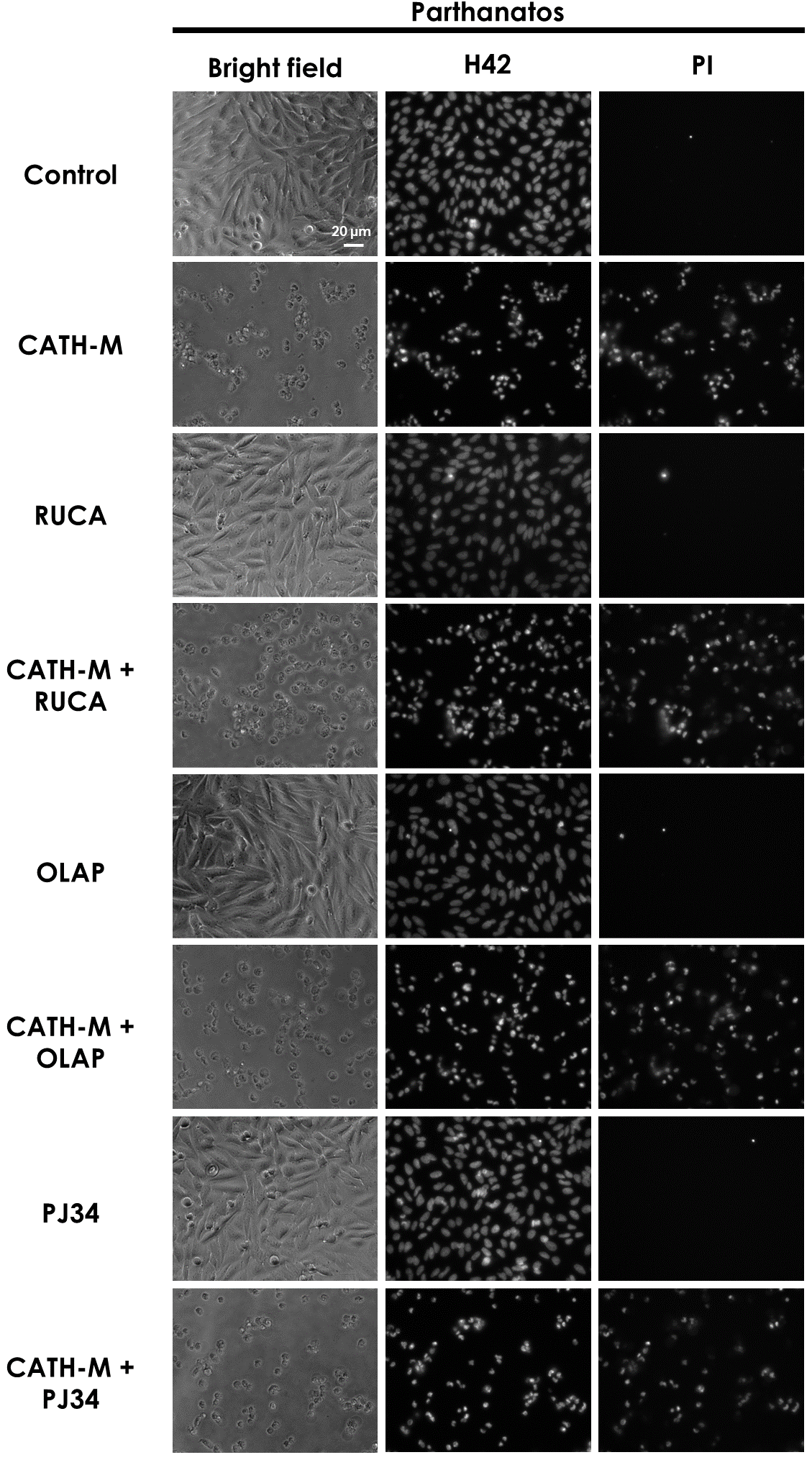


**Figure S21. CATH-M and parthanatos inhibitors.** Representative bright field, H42 and PI images of the LN229 cells after using rucaparib, olaparib and PJ34.

# **S22. CATH-M and macromolecular synthesis inhibitors.**


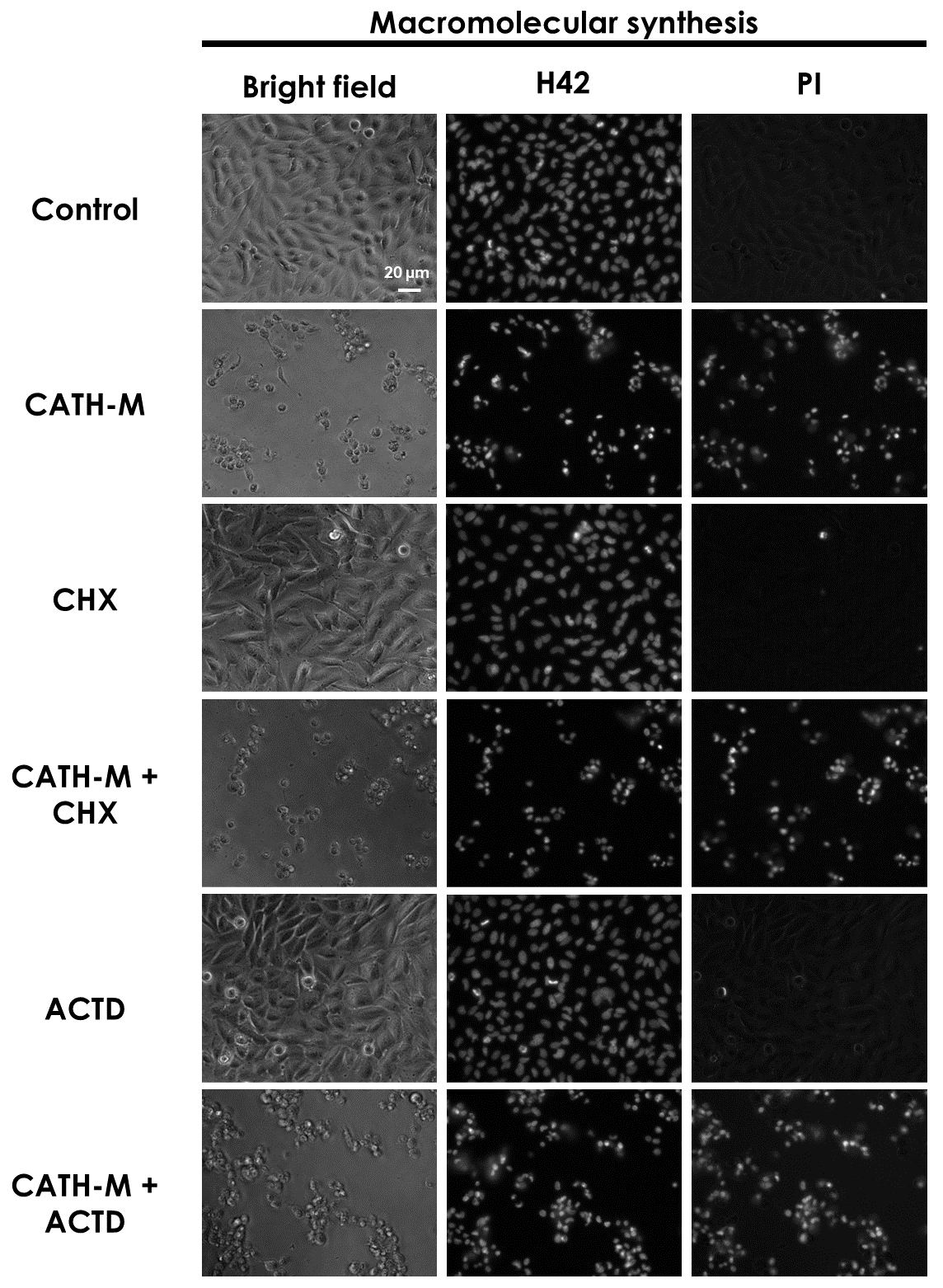


**Figure S22. CATH-M and macromolecular synthesis inhibitors.** Representative bright field, H42 and PI images of the LN229 cells after using cycloheximide and actinomycin D.

# **S23. CATH-M and ferroptosis inhibitors.**


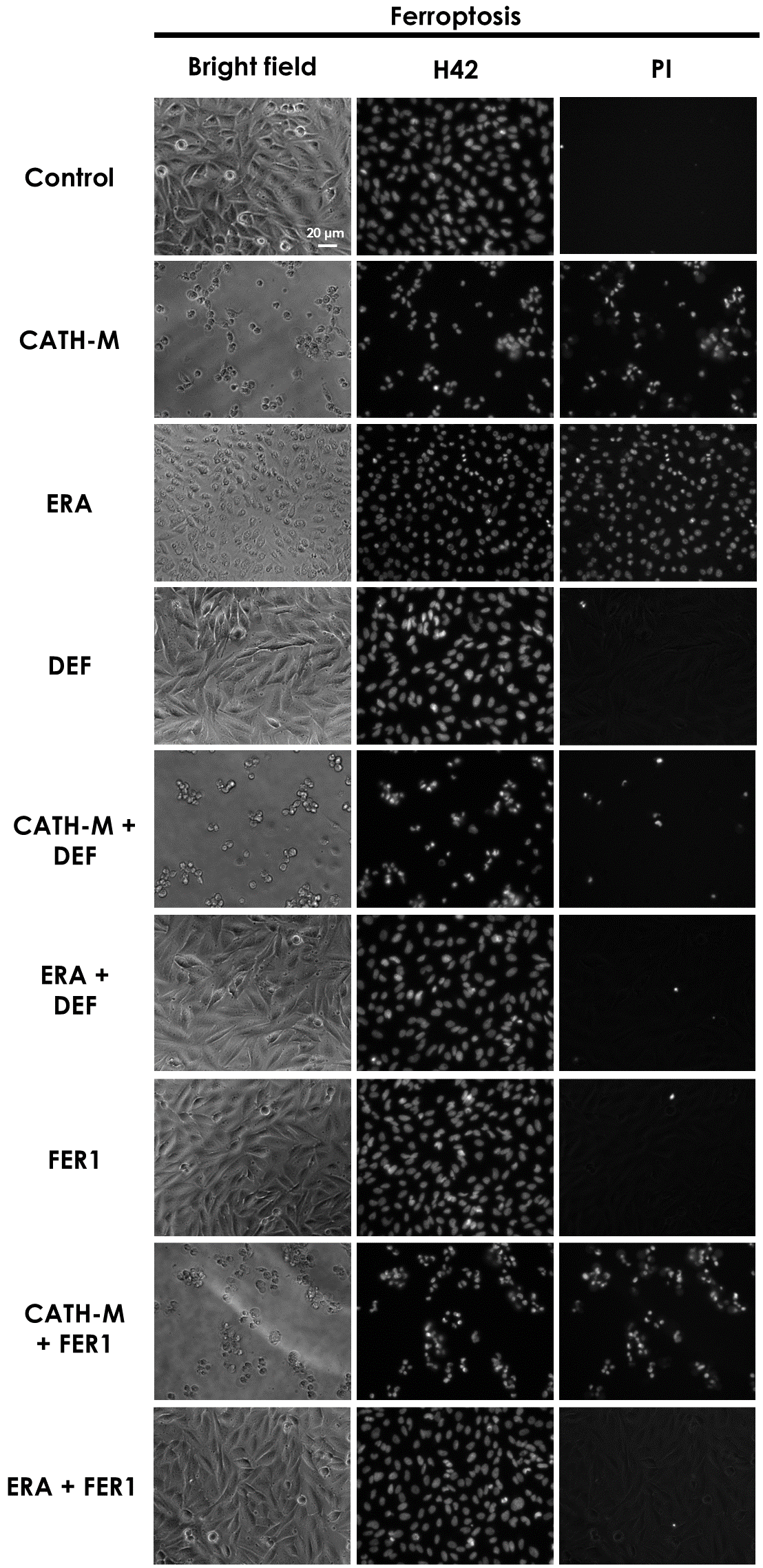


**Figure S23. CATH-M and ferroptosis inhibitors.** Representative bright field, H42 and PI images of the LN229 cells after using the inhibitors deferoxamine and ferrostatin-1, as well as the ferroptosis inducer erastine.

# **S24. CATH-M and oxidative damage inhibitor.**


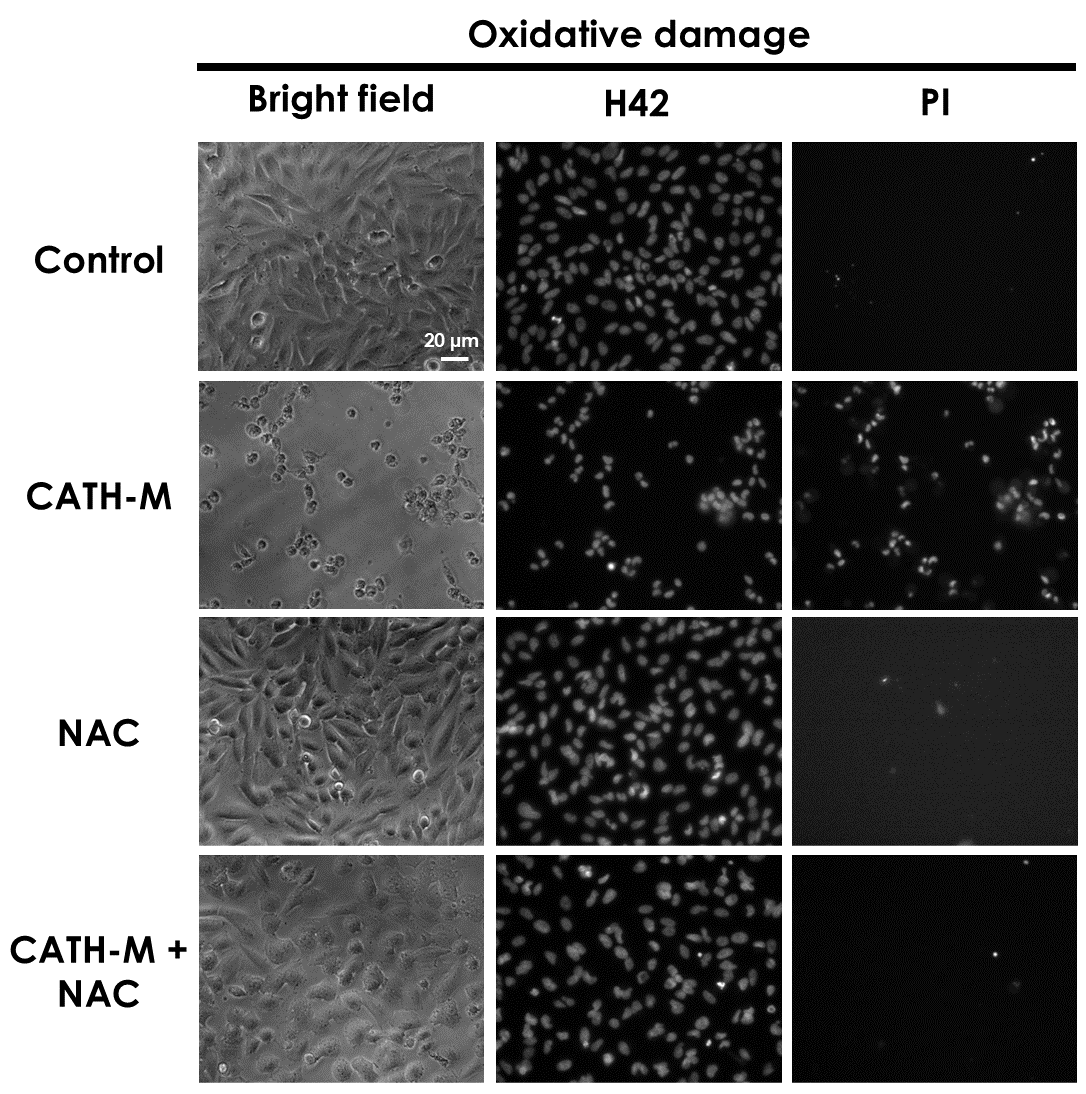


**Figure S24. CATH-M and oxidative damage inhibitor.** Representative bright field, H42 and PI images of the LN229 cells after using n-acetylcysteine.

# **S25. LN229 morphology over the membranes.**


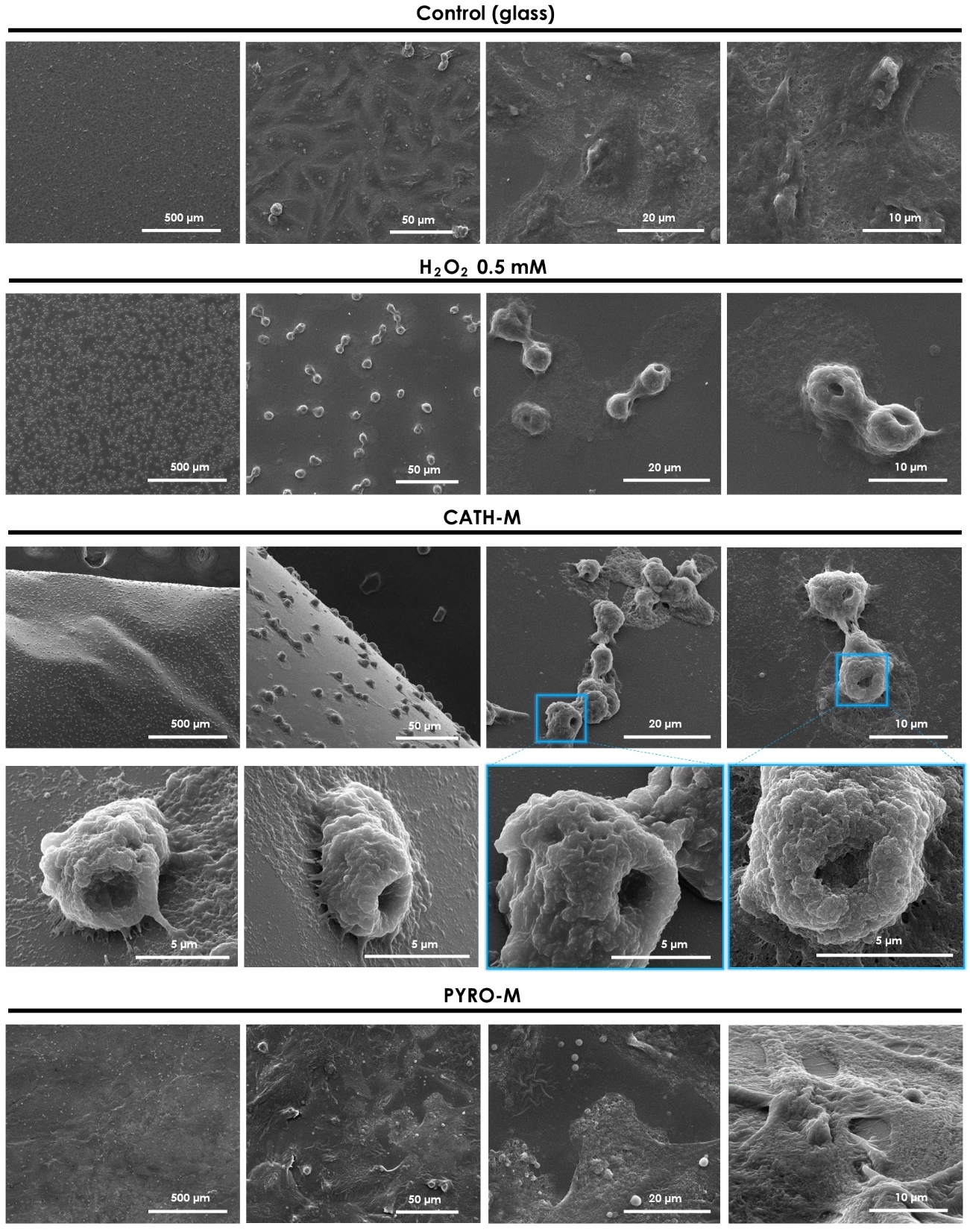


**Figure S25. LN229 morphology over the membranes.** Representative scanning electron microscopy (SEM) micrographs of the LN229 cells cultured over glass (control), as well as exposed to 0.5 mM H₂O₂, or over **CATH-** **M / PYRO-M** for 24 h**.**

# **S26. LN229 cell death over the membranes.**


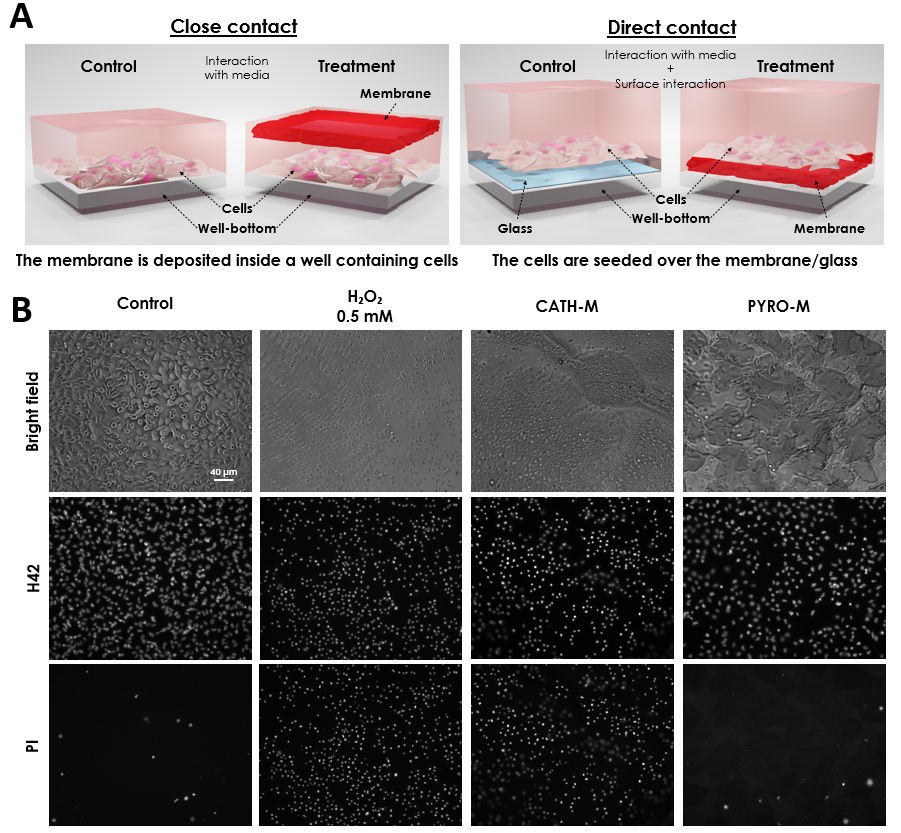


**Figure S26. LN229 cell death over the membranes. A)** Comparison between the two methodologies used to test the membrane. The close contact (left) is characterized for testing only the radical production of the membranes (interaction with media) close to the cells, but without being physically in contact with them. The membranes are tested on an already stablished monolayer. On the contrary, the direct contact (right) is characterized for using the membrane as substrate for the cell seeding (and a glass coverslip as control). In this case, not only the interaction with media is tested (which would be even stronger due the proximity of the cells to the membrane), but also the interaction of the cells with the surface of the membrane. No specific face was selected for the close contact treatment or direct contact (seeding) in the case of **PYRO-M**. For **CATH-M**, the water-face was oriented to the cells in the case of the close contact treatment, whereas the cells (in direct contact) were seeded over the air-face. Therefore, the air/water faces of the **CATH-M** membrane were always oriented as up/down, respectively. **B)** Direct contact representative Bright field, H42 and PI images of the LN229 cells cultured over glass (control), as well as exposed to 0.5 mM H₂O₂, or over **CATH-M** / **PYRO-M** for 24 h.

# **S27. Primary cortical astrocytes morphology over the membranes.**


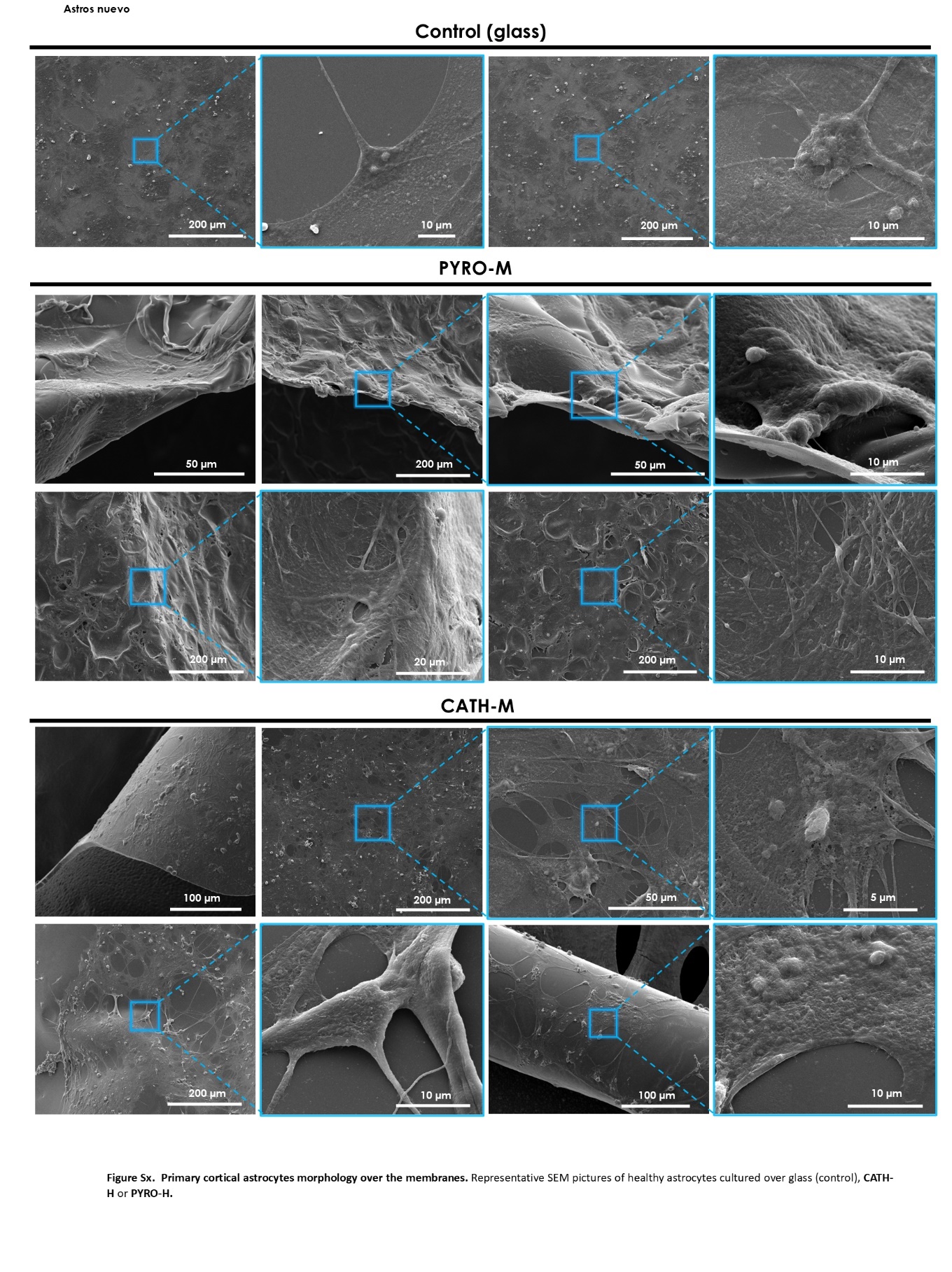


**Figure S27. Primary cortical astrocytes morphology on the membranes.** Representative SEM images of healthy astrocytes cultured for 24 h on glass (control), **PYRO-M** and **CATH-M.** No differences in the cell morphology between the control and the membranes were observed.

# **S28. Primary cortical astrocytes viability over the membranes.**


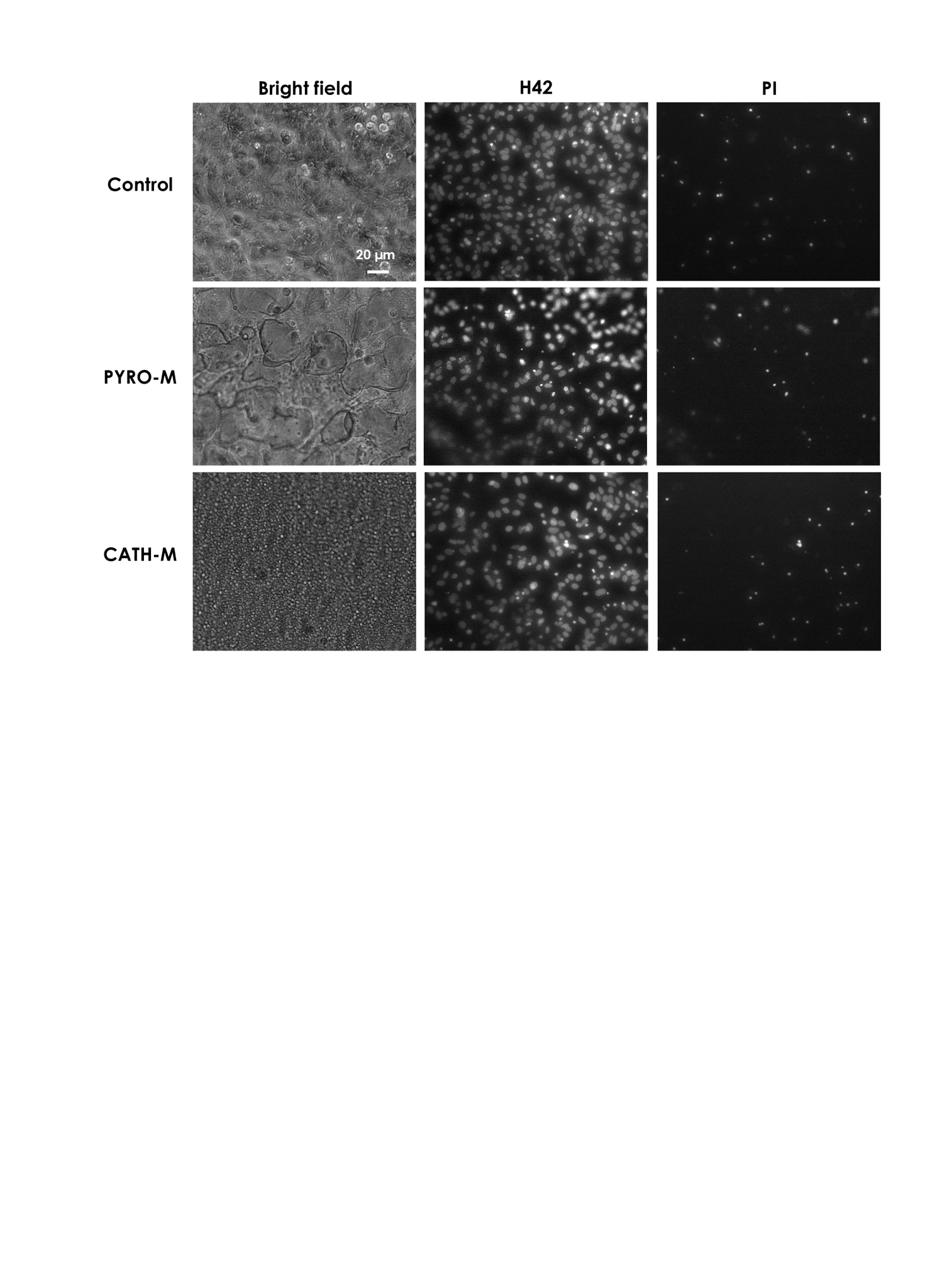


**Figure S28. Astrocytes cell death over the membranes.** Direct contact representative bright field, H42 and PI images of the healthy astrocytes cells cultured over glass (control), **PYRO-M** and **CATH-M** for 24 h.

# **S29. Spheroids early culture by scanning electron microscopy (SEM).**


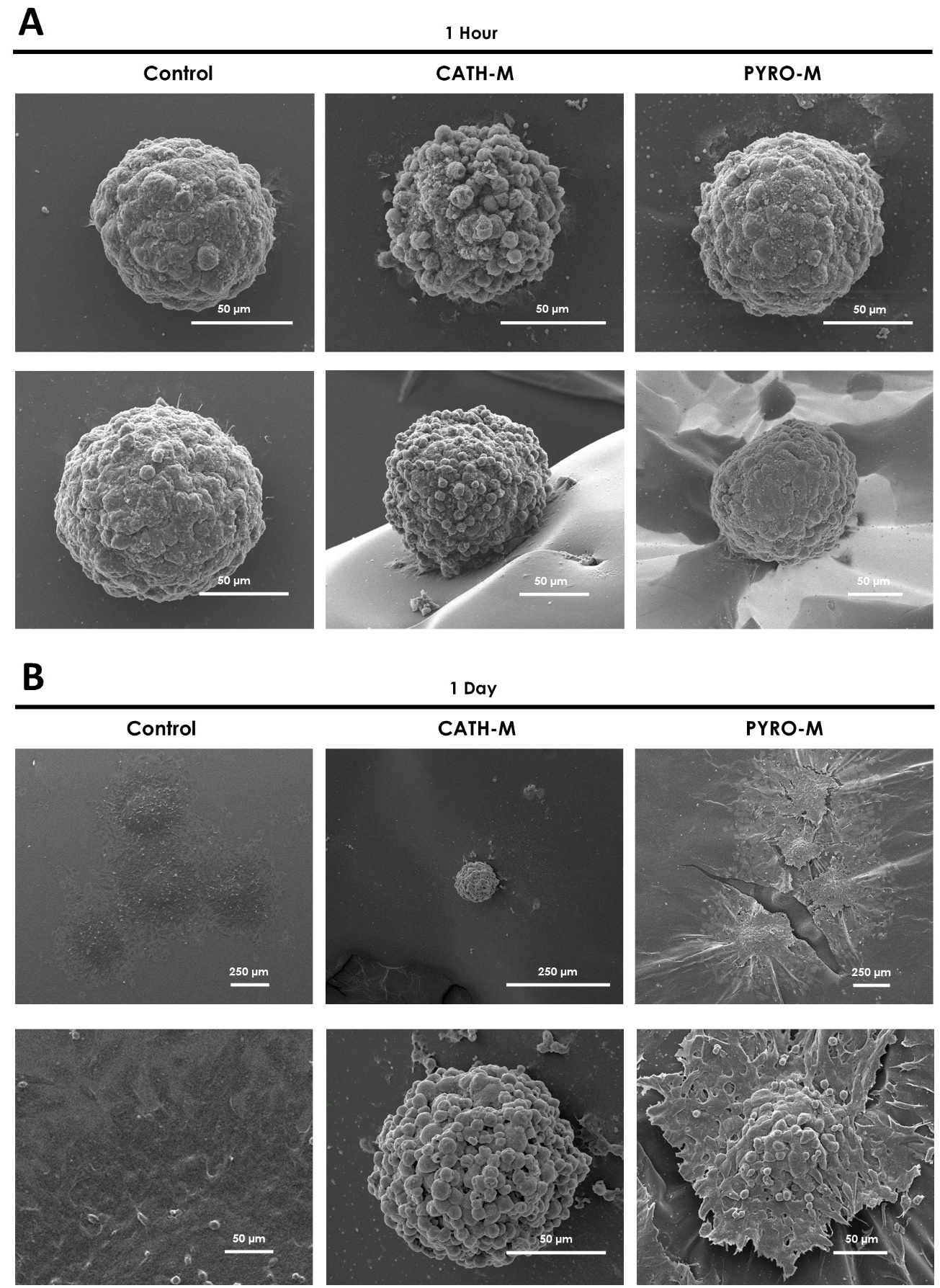


**Figure S29. Spheroids early culture by scanning electron microscopy (SEM).** Representative SEM images of LN229 spheroids cultured on glass (control), **CATH-M** for **PYRO-M** membrane for **A)** 1 h and **B)** 1 day.

# **S30. Spheroids early culture by optical microscopy.**


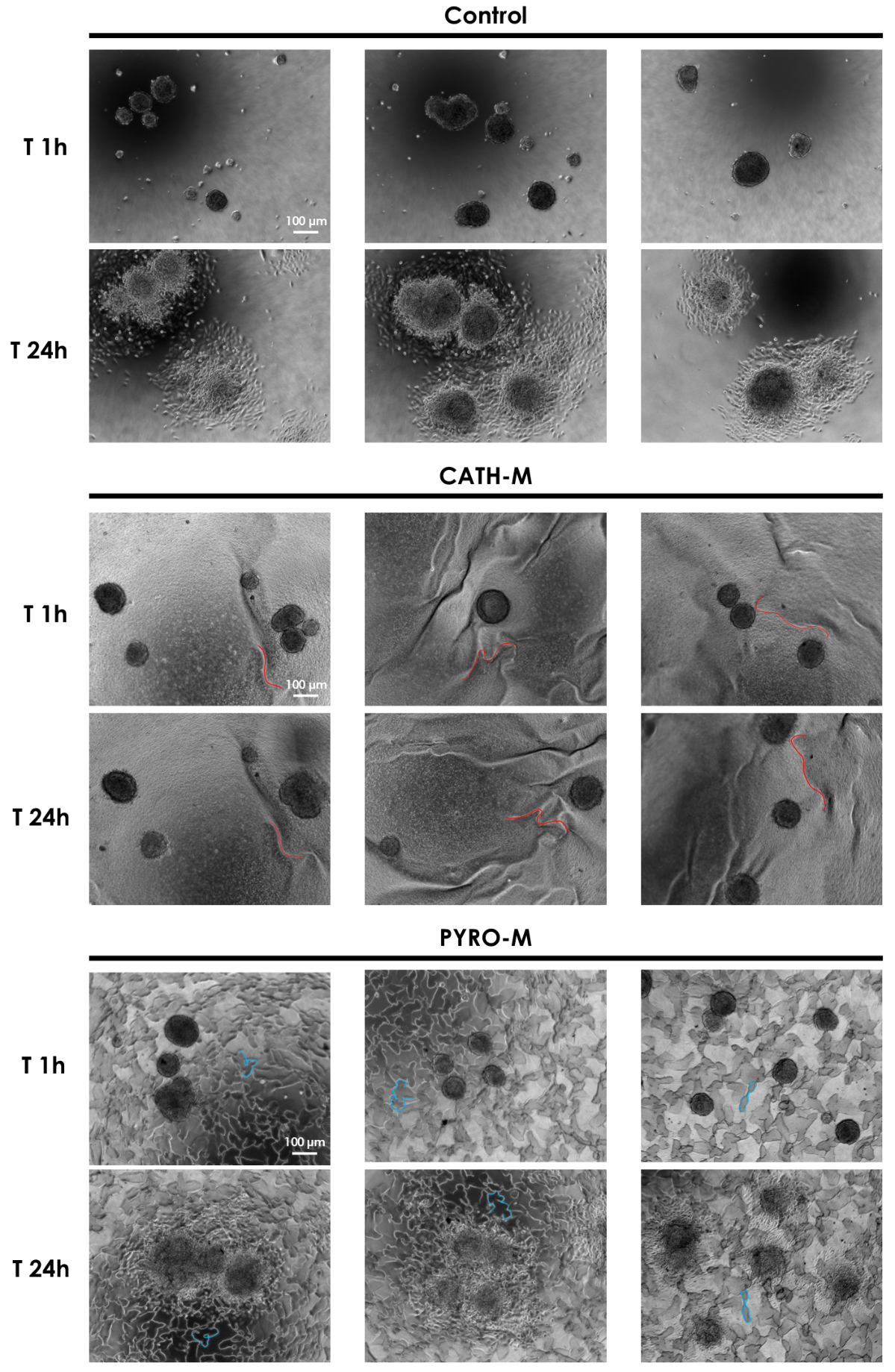


**Figure S30. Spheroids early culture by optical microscopy.** Representative bright field images of LN229 spheroids cultured on glass (control), **CATH-M** or **PYRO-M**, and their evolution after 24 h, where a significant cell migration was observed in the control and the **PYRO-M**, whereas not being perceptible in the case of the **CATH-M**. Since the membranes can rotate in the media, a specific membrane shape has been coloured (red for the **CATH-M** and blue for the **PYRO-M**) for better comparison between incubation times.

# **S31. Spheroids progression.**


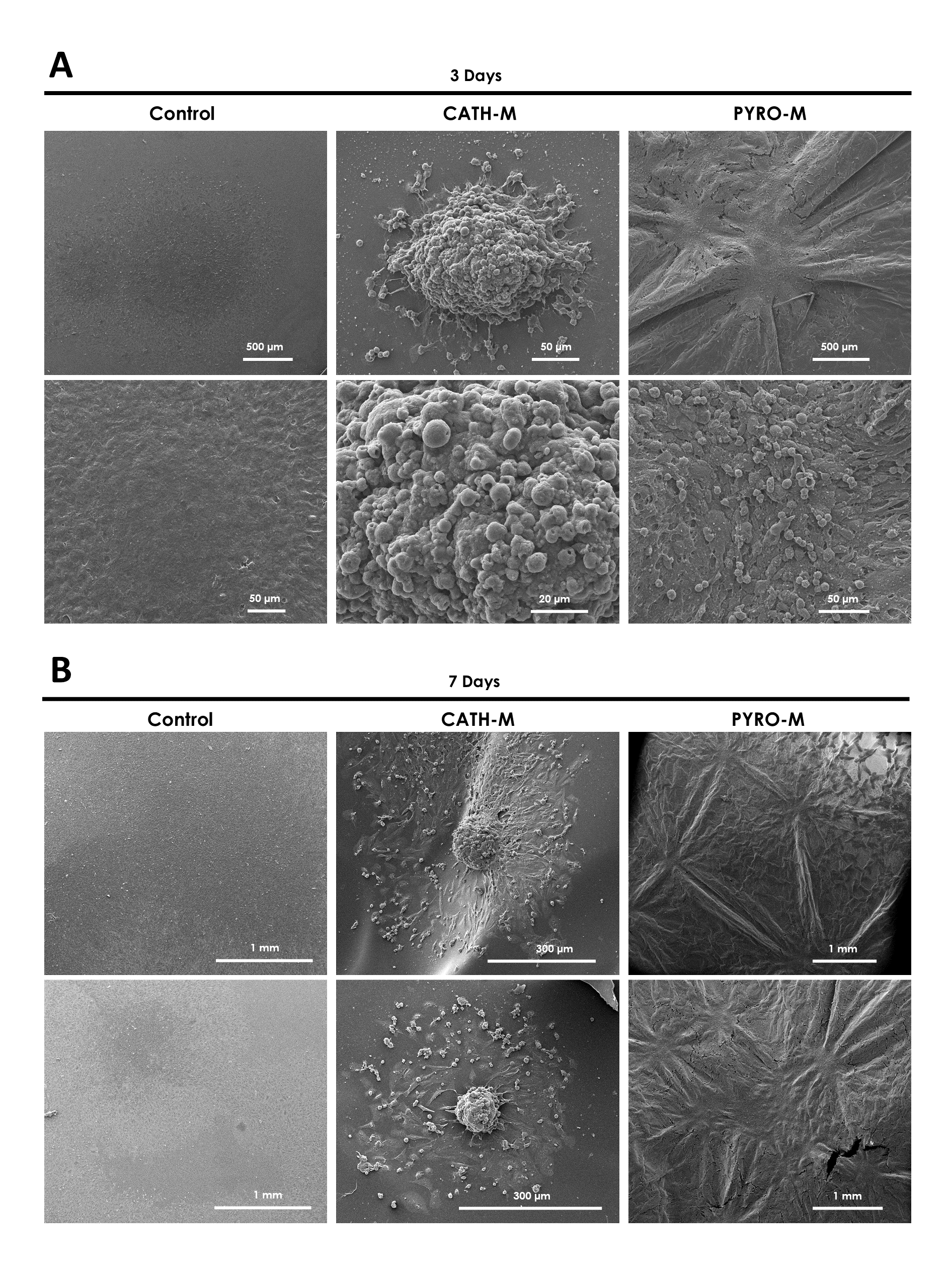


**Figure S31. Spheroids progression.** Representative SEM images of LN229 spheroids cultured on glass (control), **CATH-M** or **PYRO-M** membrane for **A)** 3 days and **B)** 7 days.

# **S32. Inserts test scheme.**


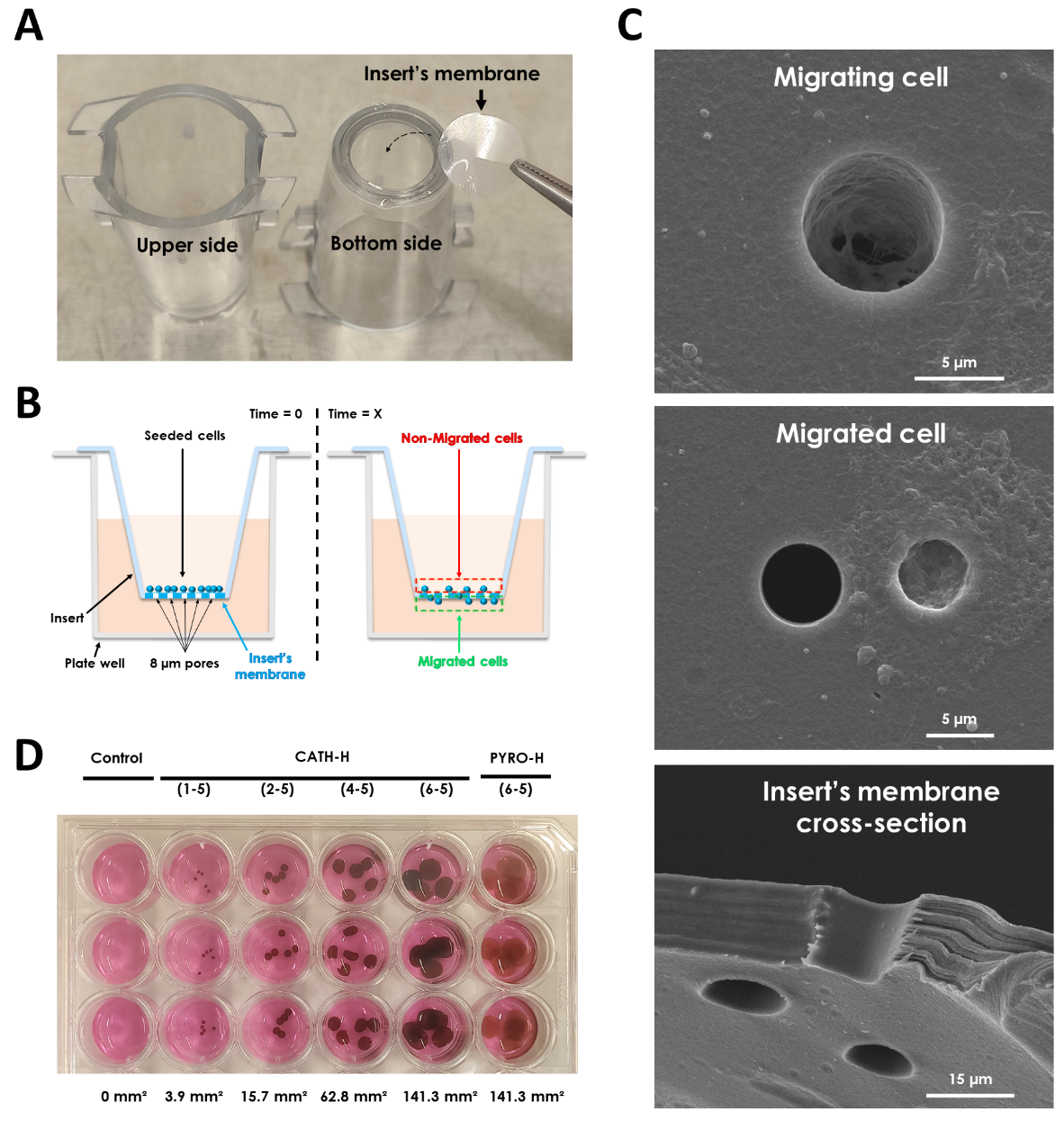


**Figure S32. Inserts test scheme. A)** Image of the selected inserts, as well as the membrane (cut) that contains the pores through the cells migrate. **B)** Schematic representation of how the inserts work. **C)** Examples of a migrating/migrated cells in the inserts, as well as the structure of its pores. **D)** Amount of material of each membrane used in the migration test, which is represented as “(disc diameter in mm - number of discs).”

# **S33. Influence in the LN229 cell migration.**


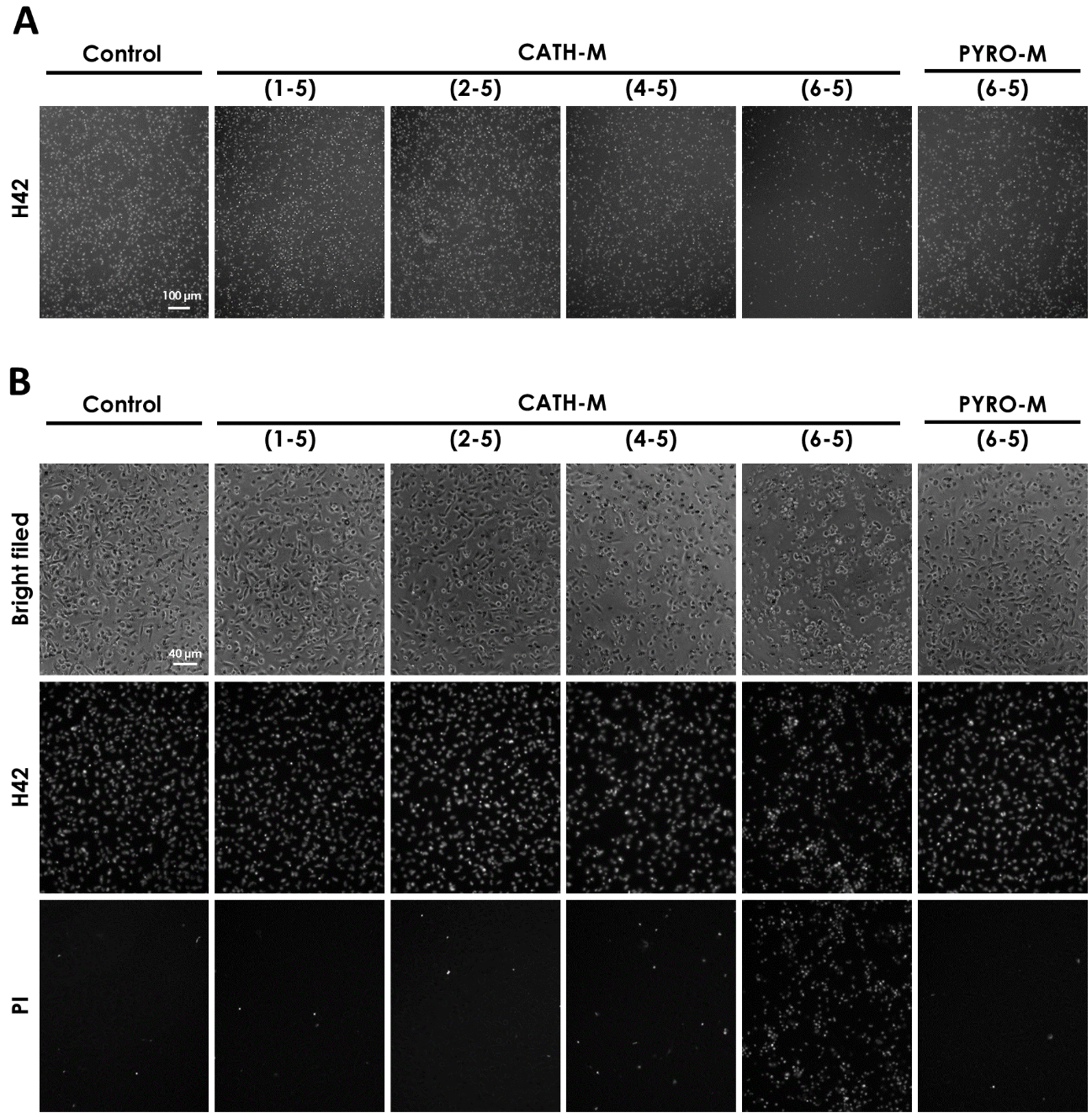


**Figure S33. Influence in the LN229 cell migration. A)** Representative H42 images of the LN229 cells that migrated to the lower face of the insert after being 16 h with increasing amounts of **CATH-M** membrane and a fixed one of **PYRO-M**. Can be clearly observed that the **CATH-M** is inhibiting the migration. **B)** Representative bright field, H42 and PI images of a similar experiment, where the cell death of the migrated and non-migrated cell (simultaneously) has been assessed, which indicates a substantial cytotoxicity in the case **CATH-M** (6-5). Amount of material of **CATH-M** used in the migration test is represented as “(disc diameter in mm - number of discs).”

# **S34. Migrated and non-migrated cells visualization.**


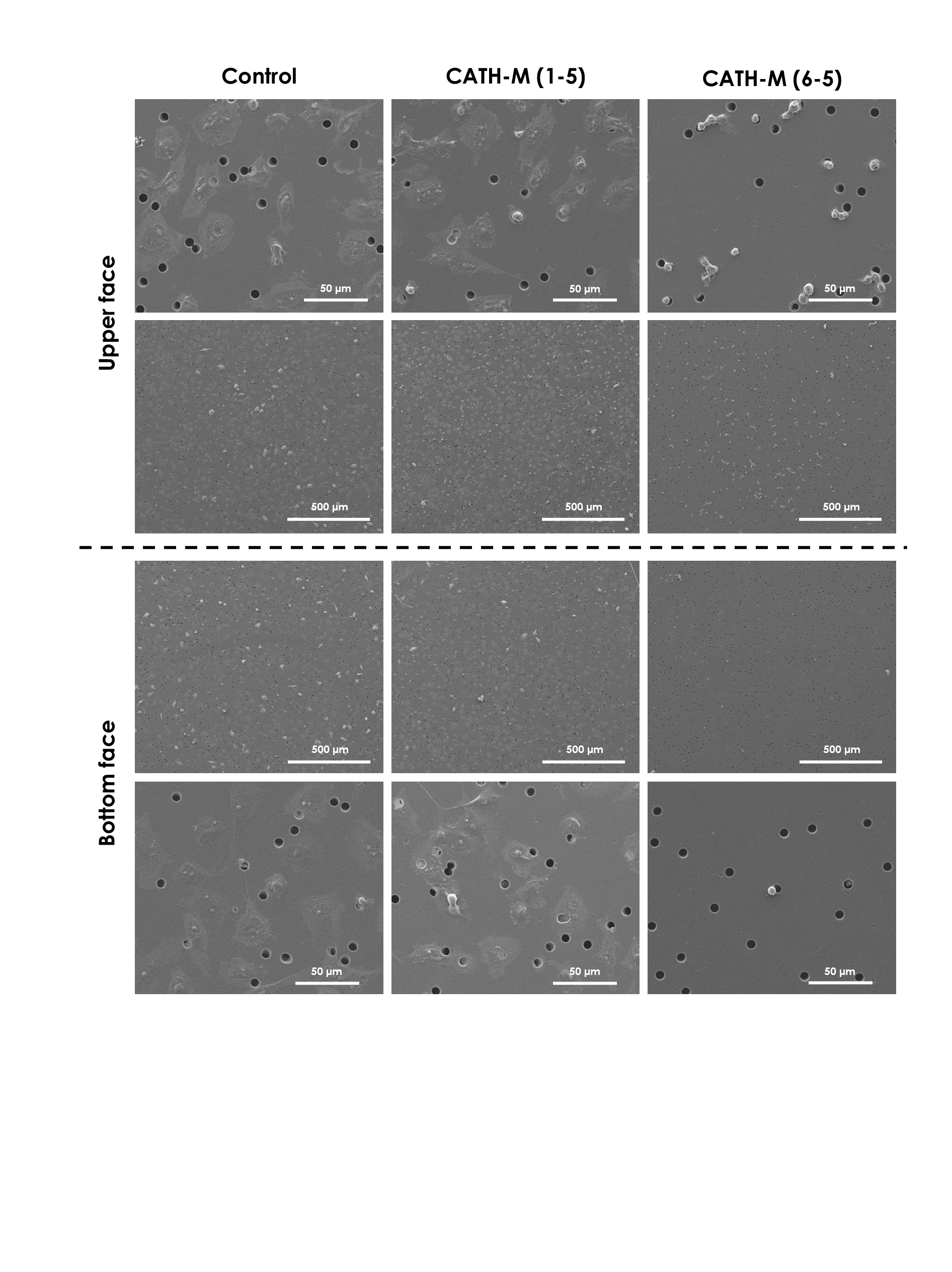


**Figure S34. Migrated and non-migrated cells visualization.** Representative SEM images of the cells remaining on each side of the membrane of the insert. The results are showing for the control and **CATH-M** with the lowest and highest amount of material. Amount of material of **CATH-M** used in the migration test is represented as “(disc diameter in mm - number of discs).”

# **S35. Membranes strong cell adhesion.**


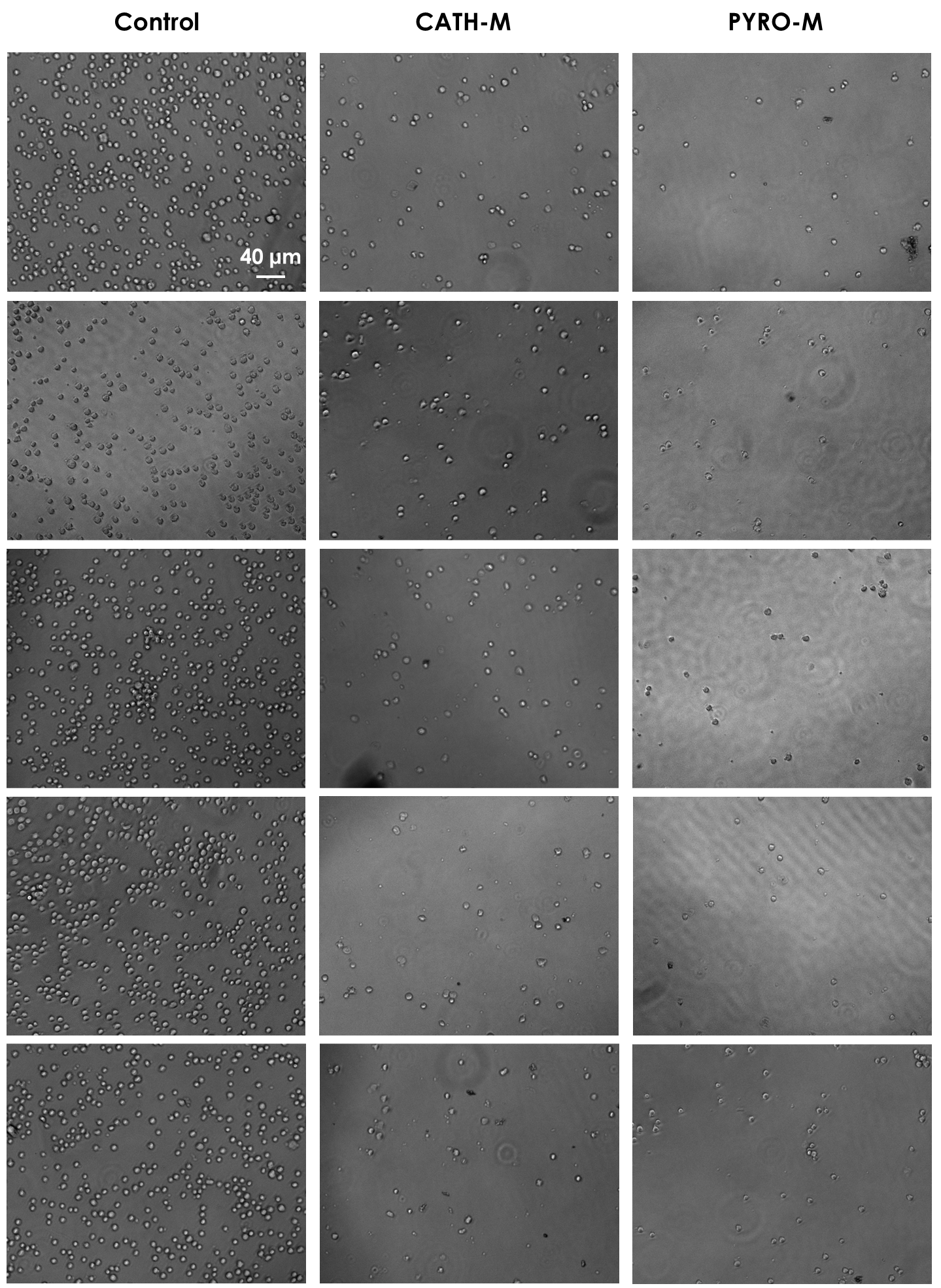


**Figure S35. Membranes strong cell adhesion.** Representative bright field images of the LN229 cells that were detached after the cells adhered to the bottom of a treated 96-well plate (control), to a **CATH-M** or to a **PYRO-M** membrane were trypsinized, suggesting that the cell adhesion over the membranes should be much stronger than the control.

# **S36. Alterations in the LN229 internal structures.**

**Figure S36.** **Alterations in the LN229 internal structures.** Representative transmission electron microscopy (TEM) micrographs of the LN229 after being exposed to **CATH-M**. Each image row shows a different cell, ordered in function of their damage stage, from early (up) to late (down). Green and red arrows indicate mitochondria and plasmatic membrane damage, respectively.

# **S37. Seahorse test.**


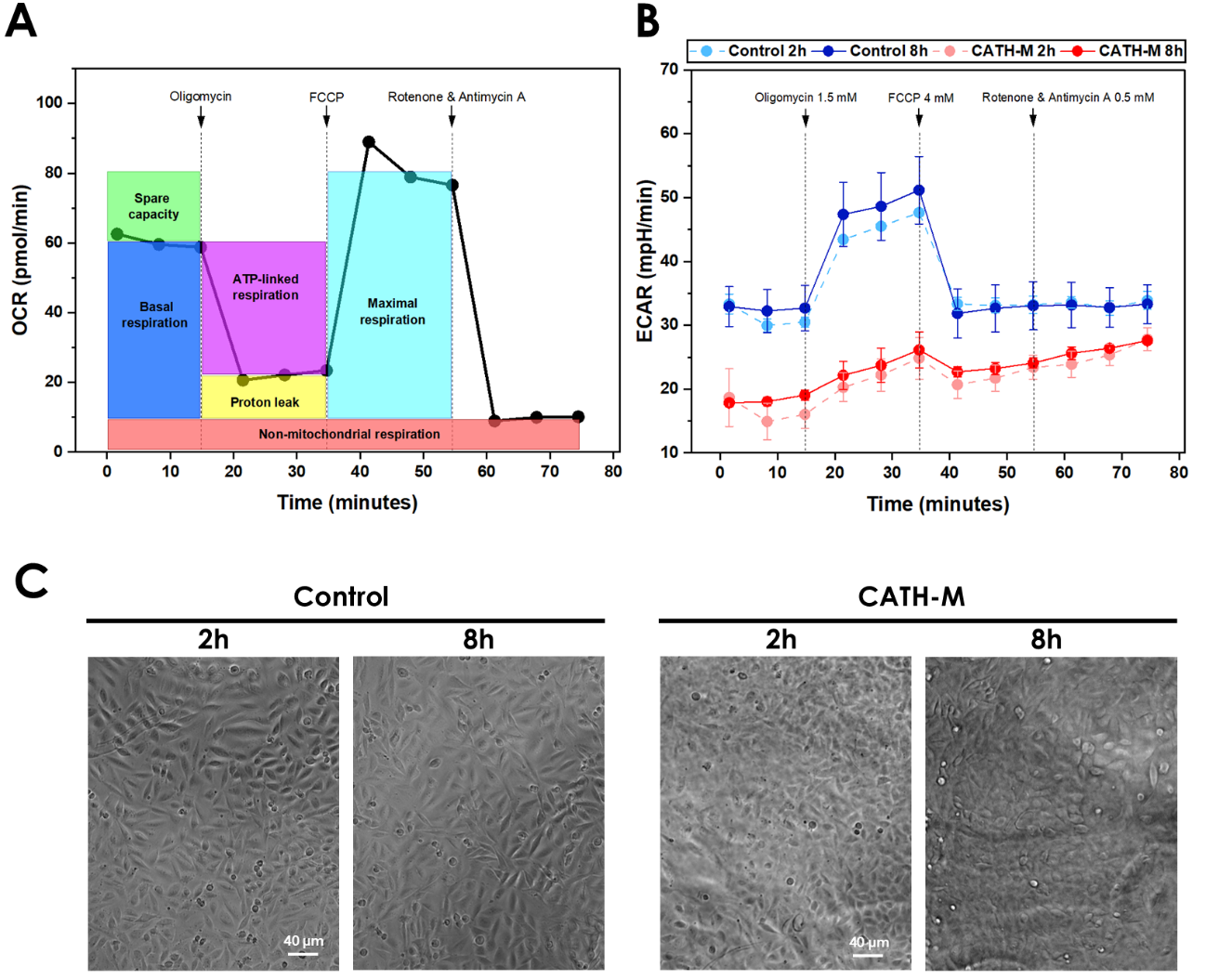


**Figure S37. Seahorse test. A)** Visual scheme of the individual parameters obtained in function of the Oxygen Consumption Rate (OCR) profile, using as example the control at 8 h. **B)** Extracellular Acidification Rate (ECAR) profile obtained simultaneously as the OCR, showing a clear decrease in the acidification of the **CATH-M** treated cells, which could be related with a lower glycolytic activity. **C)** Representative bright field LN229 cells images right before reading their metabolic profile. Error bars represent the standard deviation of a data set relative to the mean.

# **S38. LN229 cell death under oxidative stress.**


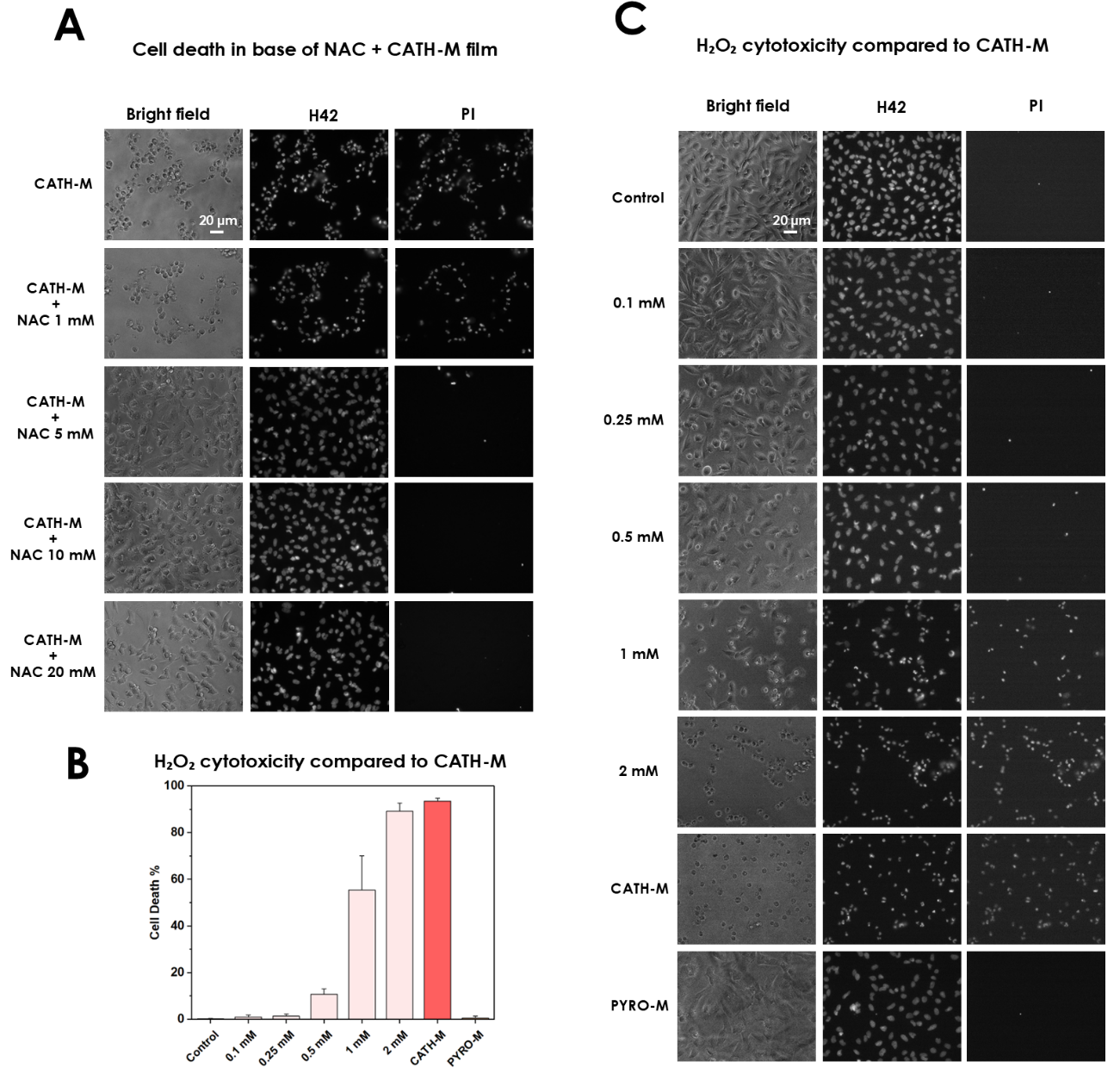


**Figure S38. LN229 cell death under oxidative stress. A)** Representative bright field, H42 and PI images of LN229 treated simultaneously with **CATH-M** and increasing molarities of the antioxidant n-acetylcysteine (NAC), being able to revert the cytotoxicity induced by **CATH-M**. **B)** Cell death percentage of the LN229 treated with increasing concentrations of hydrogen peroxide, while comparing it to **CATH-M**. **C)** Representative bright field, H42 and PI images of the experiment. Error bars represent the standard deviation of a data set relative to the mean.

# **S39. CATH-M reactive oxygen species (ROS) production properties.**


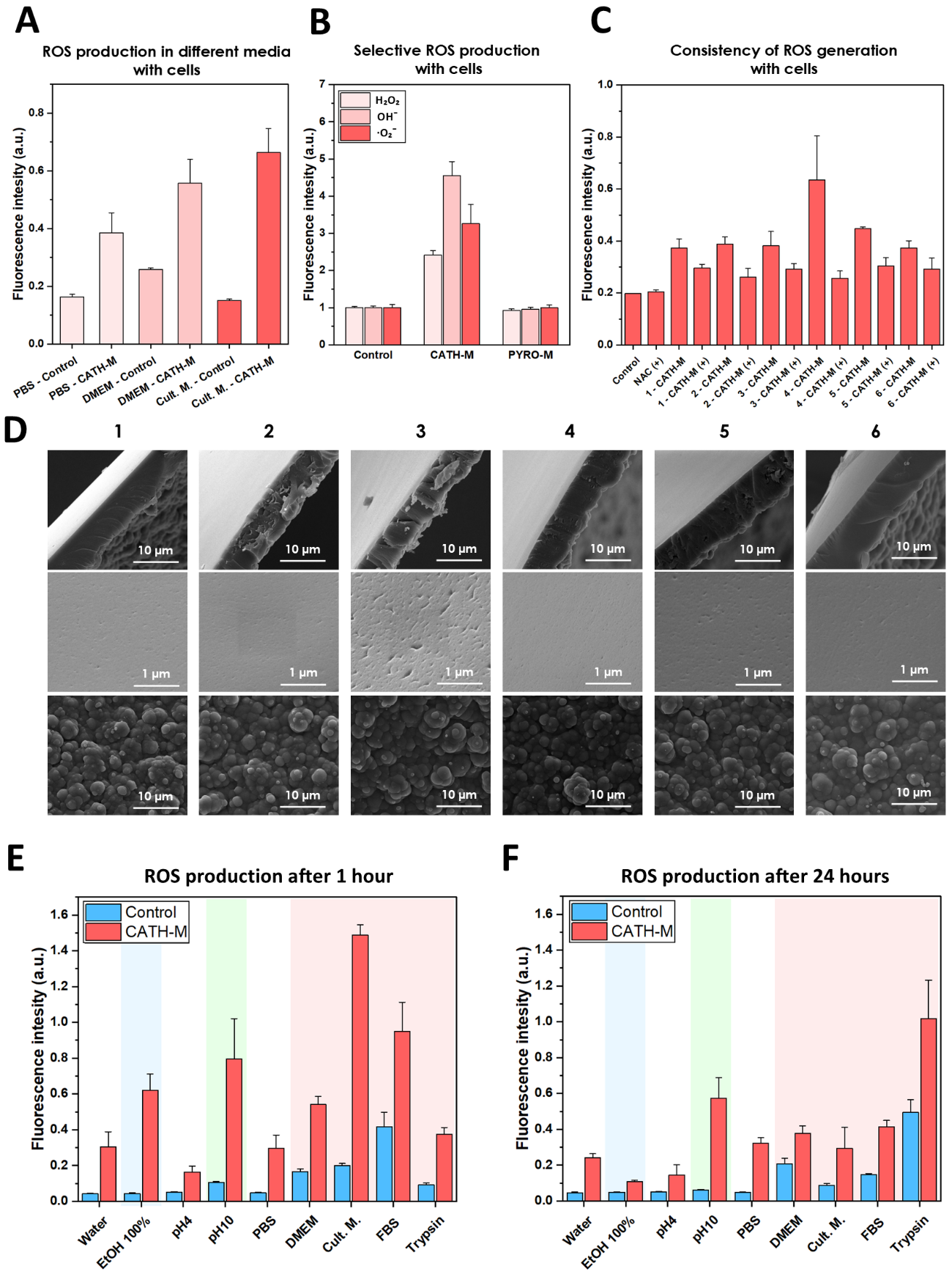


**Figure S39. CATH-M reactive oxygen species (ROS) production properties. A)** Changes in the ROS production of **CATH-M** between PBS, DMEM and culture media in presence of LN229 cells. **B)** Selective reactive oxygen species induced by **CATH-M** and **PYRO-M** membrane in presence of LN229 cells. **C)** Consistency in **CATH-M** ROS generation and NAC inactivation, represented as “(+)” between different synthesis in presence of LN229 cells, and **D)** the comparison by SEM of the 6 membranes used. **E)** **CATH-M** ROS production on different media after 1 h and **F)** 24 h of incubation. Error bars represent the standard deviation of a data set relative to the mean.

# **S40. Lipid peroxidation.**


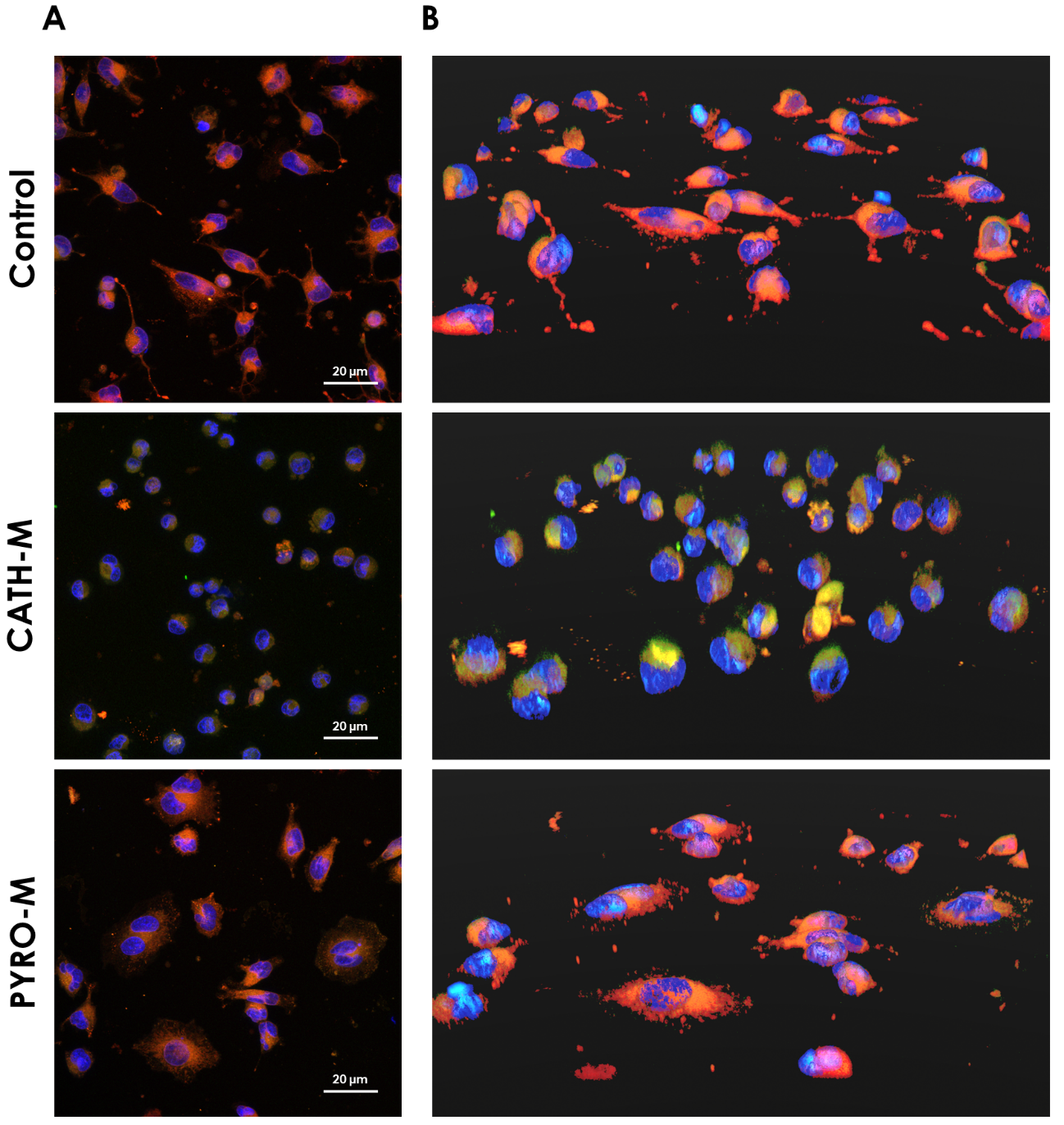


**Figure S40. Lipid peroxidation.** Confocal images (z-stacking) showing the lipid peroxidation results of the control, **CATH-M** and **PYRO-M**. Images in the left and right column are the same but in **A)** 2D and **B)** 3D view, respectively. Blue = nuclei, RED = dye reduced-state, Green= dye oxidized-state.

# **S41. LN229-lysate protein microarray.**


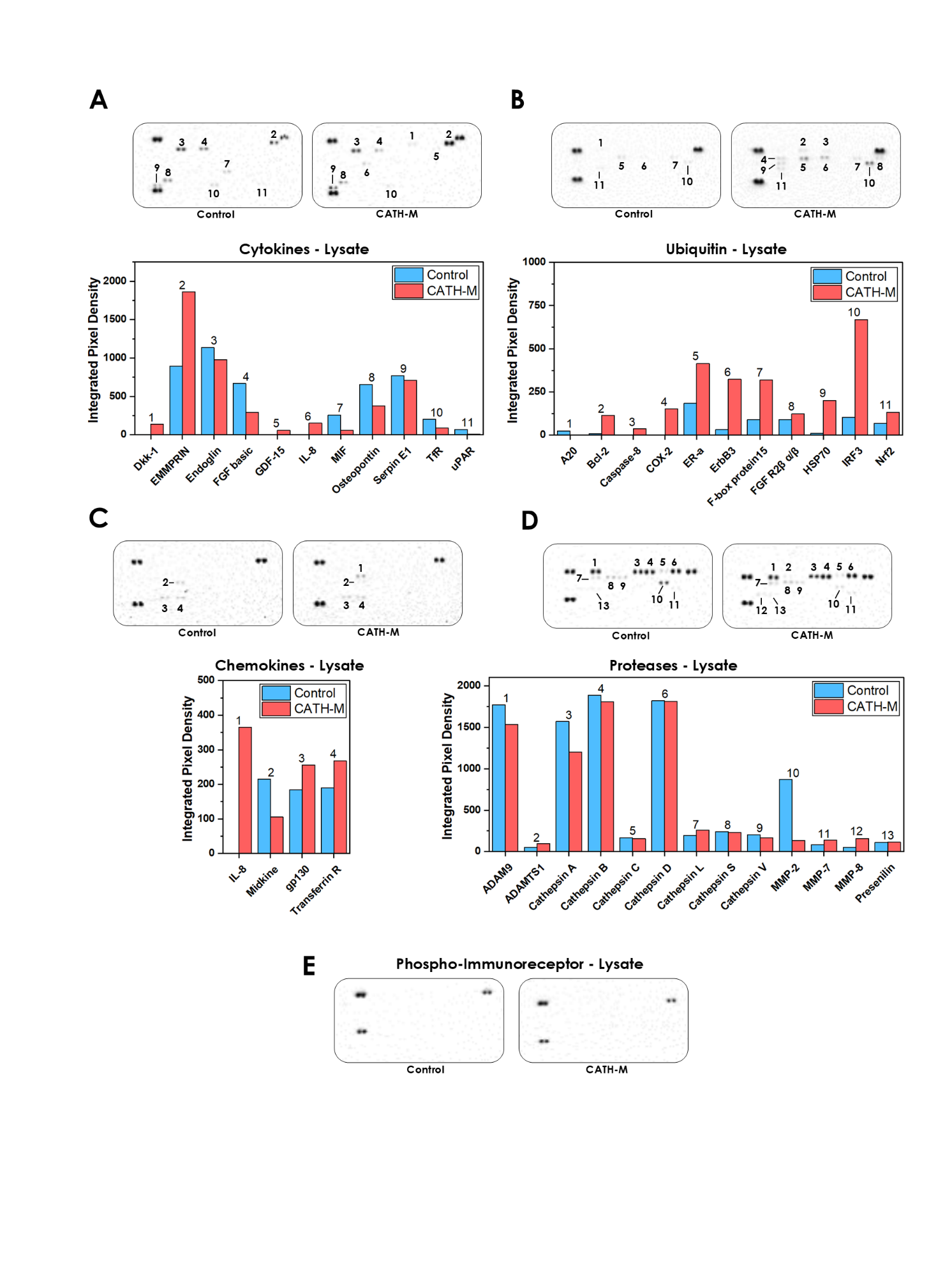


**Figure S41. LN229-lysate protein microarray.** Comparison of the signal obtained from the LN229-lysate arrays in the control and the **CATH-M** exposure and the quantification of every spot pair: **A)** cytokines, **B)** ubiquitin, **C)** chemokines, **D)** proteases, **E)** phospho-immunoreceptors (no signal obtained). Numbers above the columns represents the mean signal obtained in each spot pair of the array for both conditions.
